# Requirements Analysis and Specification for a Molecular Tumor Board Platform Based on cBioPortal

**DOI:** 10.3390/diagnostics10020093

**Published:** 2020-02-10

**Authors:** Philipp Buechner, Marc Hinderer, Philipp Unberath, Patrick Metzger, Martin Boeker, Till Acker, Florian Haller, Elisabeth Mack, Daniel Nowak, Claudia Paret, Denny Schanze, Nikolas von Bubnoff, Sebastian Wagner, Hauke Busch, Melanie Boerries, Jan Christoph

**Affiliations:** 1 Department of Medical Informatics, Friedrich-Alexander-University Erlangen-Nürnberg (FAU), 91058 Erlangen-Tennenlohe, Germany; philipp.buechner@fau.de (P.B.); marc.hinderer@fau.de (M.H.); philipp.unberath@fau.de (P.U.); 2 Institute of Medical Bioinformatics and Systems Medicine, Faculty of Medicine and Medical Center-University of Freiburg, 79110 Freiburg, Germany; patrick.metzger@mol-med.uni-freiburg.de; 3 Faculty of Biology, University of Freiburg, 79104 Freiburg, Germany; 4 Institute of Medical Biometry and Statistics, Faculty of Medicine and Medical Center, University of Freiburg, 79104 Freiburg, Germany; martin.boeker@imbi.uni-freiburg.de; 5 Institute of Neuropathology, Justus-Liebig-University Giessen, 35392 Giessen, Germany; till.acker@patho.med.uni-giessen.de; 6 Institute of Pathology, University Hospital Erlangen, 91054 Erlangen, Germany; florian.haller@uk-erlangen.de; 7 Department of Hematology, Oncology and Immunology, Philipps-University Marburg, and University Hospital Gießen and Marburg, Marburg, Germany Baldingerstraße, 35043 Marburg, Germany; elisabeth.mack@staff.uni-marburg.de; 8 Department of Hematology and Oncology, Medical Faculty Mannheim, Heidelberg University, 68167 Mannheim, Germany; daniel.nowak@medma.uni-heidelberg.de; 9 Heinrich-Lanz-Center for Digital Health, Medical Faculty Mannheim, Heidelberg University, 68167 Mannheim, Germany; 10 Pediatric Hematology/Oncology, Children’s Hospital, University Medical Center of the Johannes Gutenberg-University Mainz, 55131 Mainz, Germany; paretc@uni-mainz.de; 11 University Cancer Center (UCT) of the University Medical Center of the Johannes Gutenberg-University Mainz, 55131 Mainz, Germany; 12 Institute of Human Genetics, University Hospital Magdeburg, Faculty of Medicine, Otto-von-Guericke University, 39120 Magdeburg, Germany; denny.schanze@med.ovgu.de; 13 Department of Hematology and Oncology, Medical Center, University of Schleswig-Holstein, Campus Lübeck, 23538 Lübeck, Germany; nikolas.bubnoff@uniklinik-freiburg.de; 14 German Cancer Consortium (DKTK), partner site Freiburg, 79106 Freiburg, Germany; 15 Department of Hematology, Oncology and Stem Cell Transplantation, Medical Center, Faculty of Medicine, University of Freiburg, 79106 Freiburg, Germany; 16 Department of Medicine 2, Hematology/Oncology, Goethe University Hospital, 60590 Frankfurt am Main, Germany; swagner@med.uni-frankfurt.de; 17 Institute of Experimental Dermatology and Institute of Cardiogenetics, University of Lübeck, 23562 Lübeck, Germany; hauke.busch@uni-luebeck.de; 18 Comprehensive Cancer Center Freiburg (CCCF), Faculty of Medicine and Medical Center – University of Freiburg, 79106 Freiburg, Germany; 19 German Cancer Consortium (DKTK), partner site Freiburg; and German Cancer Research Center (DKFZ), 69120 Heidelberg, Germany

**Keywords:** decision making, computer-assisted, decision support systems, clinical, precision medicine, computational biology, molecular tumor board, cBioPortal, requirements analysis, neoplasms

## Abstract

Clinicians in molecular tumor boards (MTB) are confronted with a growing amount of genetic high-throughput sequencing data. Today, at German university hospitals, these data are usually handled in complex spreadsheets from which clinicians have to obtain the necessary information. The aim of this work was to gather a comprehensive list of requirements to be met by cBioPortal to support processes in MTBs according to clinical needs. Therefore, oncology experts at nine German university hospitals were surveyed in two rounds of interviews. To generate an interview guideline a scoping review was conducted. For visual support in the second round, screenshot mockups illustrating the requirements from the first round were created. Requirements that cBioPortal already meets were skipped during the second round. In the end, 24 requirements with sometimes several conceivable options were identified and 54 screenshot mockups were created. Some of the identified requirements have already been suggested to the community by other users or are currently being implemented in cBioPortal. This shows, that the results are in line with the needs expressed by various disciplines. According to our findings, cBioPortal has the potential to significantly improve the processes and analyses of an MTB after the implementation of the identified requirements.

## 1. Introduction

Advances in next-generation sequencing (NGS) technology and the resulting decrease of costs hold large promises for personalized medicine, currently revolutionizing cancer diagnostics in particular. The sequencing of whole tumor exomes, genomes and transcriptomes of patients allows physicians to make individual molecular-guided decisions. However, the complex nature of cancer and its large interindividual heterogeneity require an interdisciplinary board composed of medical and scientific experts to review and interpret the equally complex analysis results. Recent data suggest that such molecular tumor boards (MTBs) have the potential to improve therapy and care for patients that have run out of guideline-based treatment options or have rare tumors [1,2].

Several German medical centers have already started to implement MTBs in their clinical environment, all working with various types of omics data from, e.g., NGS and other technologies [3,4]. To handle the many results from a large amount of omics data there is a high need for a standardized toolset that supports clinicians in analyzing and interpreting these data and creating high-quality presentations of complex multi-dimensional data effectively. Moreover, it requires both the integration of clinical with molecular and genomic data and the visualization of joint analysis results. However, experiences with the implementation and establishment of information technology (IT) and bioinformatics support for MTBs are still rare in Germany and probably world-wide, thus in need of improvement and optimization [5].

To address this issue, the MIRACUM consortium (Medical Informatics in Research and Care in University Medicine) has established the Use Case 3 which focuses on the provision of IT and bioinformatics support for translation and visualization of data analyzed in MTBs. As part of this use case, we will establish a generic, open-source framework that supports the analysis, interpretation and visualization of both clinical and omics data [6]. Data analysis is handled through MIRACUM-Pipe [7] which provides automatic, parameter-controlled processing of omics data with alignment, variant calling, annotation and analysis. The second aspect of the framework will be the data visualization and documentation of the results of the MTB. Both, the pipeline and the visualization, will be provided as separate and open-source components developed in a user-centered design process.

The cBio Cancer Genomics Portal (cBioPortal) was selected as a suitable platform to visualize the generated data supplied by the MIRACUM-Pipe [7] and to support the decision-making processes in an MTB. cBioPortal provides an extensive set of tools for exploring, visualizing and analyzing multi-dimensional and large-scale cancer genomics data sets [8,9]. Within the context of an MTB, cBioPortal may support case preparation, case review, and the documentation and communication of treatment recommendations in the near future. Therefore, it is well suited to replace the current practice at some German university hospitals of managing complex mutation data in huge spreadsheets by providing comprehensible visualizations [10,11]. At the participating clinics, cBioPortal could optimize the processing of up to 200 cases per year with sometimes hundreds of identified but not necessarily relevant mutations, and thus improve the decision making [5].

The integration of cBioPortal into the workflow of MTBs requires adjustments regarding different functionalities and needs (requirements). For instance, to meet some of our requirements the user needs to have write access to the data stored in cBioPortal. Therefore, we must find a proper solution to accomplish this in line with the concepts cBioPortal currently pursues as a (read-only) data warehouse.

The objective of this work is to provide a requirements specification for an IT platform based on cBioPortal that supports processes in molecular tumor boards to find and document a therapy recommendation. To our knowledge, there is so far no systematic assessment of such requirements from the point of view of MTB participants in different hospitals, even though there are already existing tools that integrate numerous data sources and even support the documentation process in a uniform MTB tool [12,13]. Our work could serve as a blueprint for the development of further tools based on cBioPortal for MTBs in Germany and worldwide.

## 2. Materials and Methods

To identify the requirements, we conducted a qualitative research to assess a set of potential requirements in two consecutive rounds of interviews. The second round of interviews became necessary because the first round was developed iteratively and therefore not all participants had the chance to comment on all mentioned potential requirements.

**Figure 1:**
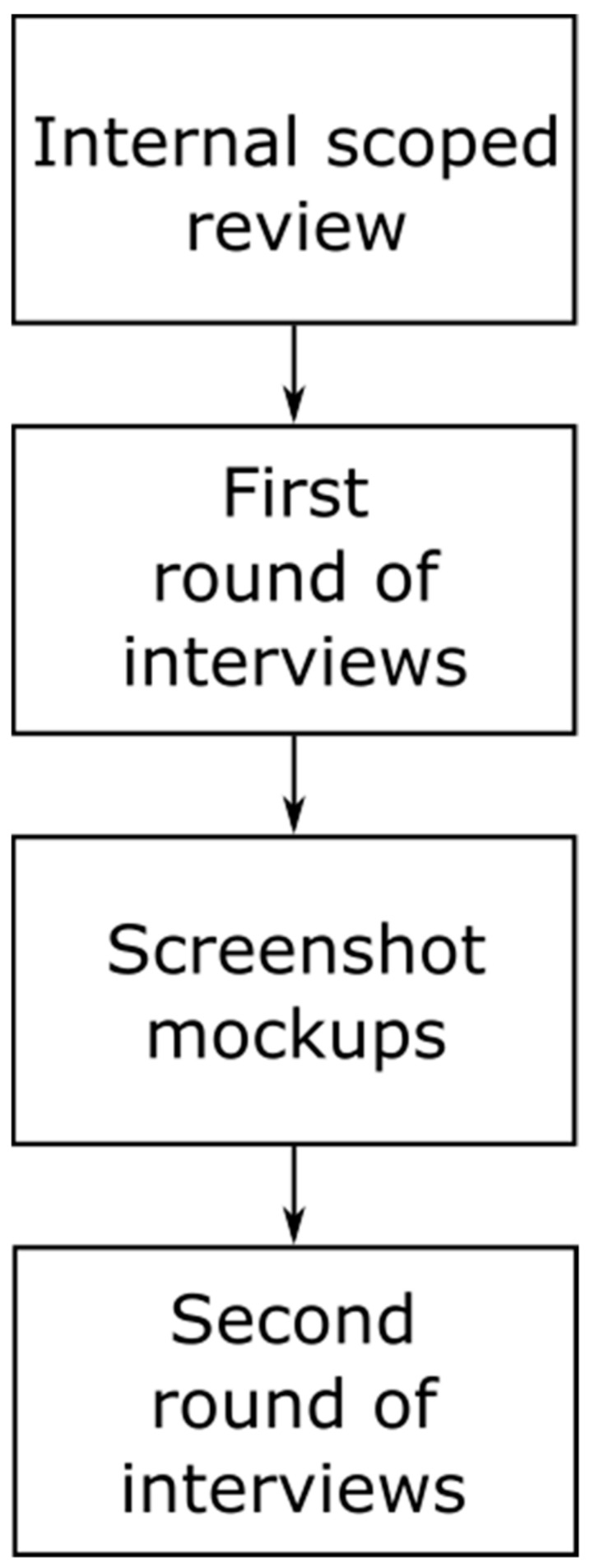
Outline of the process of requirements analysis.

In preparation, we reviewed the literature published between 1997 and 2017 (scoped review) for existing systems, tools and knowledgebases that support molecular tumor boards (see Figure 1), resulting in an interview guideline for the first round of interviews.

Based on almost all assessments from the first round of interviews, for the second round, we created screenshot mockups for better understanding and visualization of possible implementations in cBioPortal.

### 2.1. Details about Scoping Review

We conducted a review of literature for potential MTB tools following the Preferred Reporting Items for Systematic Reviews and Meta-Analysis (PRISMA) guidelines [14,15] as far as appropriate for the requirements analysis. Therefore, we searched MEDLINE and Web of Science (all databases) for articles focusing on MTBs or equivalent clinical decision-making structures published between 1997 and 2017. We captured several features of different potential MTB tools which are either described in the literature or used by physicians at our MIRACUM sites. We used these findings to prepare for the first round of interviews. A detailed description of the methods we used can be found in Supplementary File 1.

### 2.2. First and Second Round of Interviews

Based on this prior knowledge, we conducted one group interview per partner site and per round from a constructivist point of view. We hypothesized that each site would have different views and visions on requirements for supporting a local MTB. Therefore, we took all suggestions regarding the demands of the participants into account.

The interviews were conducted in local focus groups, in which the interviewers served as moderators. This approach allowed discussions between the participants and thus as many requirements and their potential variants as possible could be identified. At each site, all participants were interviewed together in one session. The interviewers had an interdisciplinary background: medicine (Philipp Buechner, Melanie Boerries), medical informatics (Jan Christoph, Marc Hinderer), bioinformatics (Jan Christoph, Melanie Boerries) and biology (Melanie Boerries).

All interviews, except the one at the first author’s local university hospital, took place as web conferences with transmission of voices and screen contents. In addition to the option of easily recording the session, the major argument for this setting was that the participants at the various university hospitals were spread all over Germany.

All interview participants were members of the MIRACUM Use Case 3 and thus known to us. The members responsible for the use case at each site arranged an appointment and invited additional local experts, who all were also members of the use case. All participants had to have experiences with the processes related to an MTB in order to join the interviews.

#### 2.2.1. Structure and Purpose of the First Round of Interviews

Philipp Buechner, Marc Hinderer and Jan Christoph conducted the interviews of the first round together as members of MIRACUM’s Use Case 3 between June 2018 and August 2018. It comprised a short guideline with questions (see Supplementary File 2) we developed from the results of the scoped review. We also demonstrated the main functionalities (see Supplementary Files 3 and 4) of the following potential MTB tools:

- cBioPortal [9],
- OncoKB [16],
- SOPHiA GENETICS [17],
- Clarivate “Key Pathway Advisor” [18],
- Clarivate “MetaCore” [18] and
- “CIViC” (Clinical Interpretations of Variants in Cancer) [19].

We collected all mentioned requirements cBioPortal must meet (including details about potential options for implementation) that were mentioned during the meetings. The interview process was developed iteratively and the information gained was immediately incorporated into the subsequent interviews with other partner sites during this first round. We used the web conference system “Adobe Connect” to conduct, record and subsequently analyze these interview sessions.

#### 2.2.2. Structure and Purpose of the Second Round of Interviews

The second round of interviews was performed by Philipp Buechner, Melanie Boerries and Jan Christoph between November 2018 and December 2018. In order to make optimal use of the limited time during the interviews, Philipp Buechner developed a comprehensive interview guideline describing the requirements and their potential options identified in the first round with text and screenshot mockups (see Supplementary File 5). However, this round did not cover the requirements mentioned in the first round of interviews, that are already implemented in cBioPortal or are generally out of the scope of MIRACUM Use Case 3. To familiarize the participants with the requirements, this guideline—once it was finally validated by Melanie Boerries, Jan Christoph and Philipp Unberath —was handed out to them prior the meetings.

Since some requirements had more than one potential option regarding implementation and visualization, sites were asked to select one during this round of interviews. In case they had different opinions, they were encouraged to find a compromise.

For the final software specification—after all interviews have been conducted and analyzed - we grouped related features into larger meta-categories to account for individual requirements and yet to keep the assessment structured. For example, the term “sample metadata”, comprises six (individual) data features:

- Localization and time of the sampling;
- Type of sampling (e.g., fine-needle aspiration biopsy);
- Distinction between fresh-frozen and formalin-fixed paraffin-embedded samples;
- Scope of sequencing (e.g., gene panel or whole-exome sequencing);
- Name and version of both the used panel and kit;
- Hyperlink to the corresponding product-specific website of the manufacturer.

When calculating the total number of requirements identified by us, we only counted those combined meta categories. Therefore, the above-mentioned example of “sample metadata” counts as one requirement instead of six individual ones.

We used the web conference system “Zoom” to conduct, record and subsequently analyze these interview sessions.

### 2.3. Low-Fidelity Mockup Demonstrator

We created 54 descriptive screenshot mockups for almost all options of the identified requirements from the first round of interviews using the image-editing tool GNU Image Manipulation Program (GIMP), version 2.10.8. These low-fidelity mockups are based on full-screen screenshots of the cBioPortal graphical user interface and have been manipulated to give the realistic appearance of providing the respective functions. To quickly direct the viewer’s focus to the part of the image that represents the demanded function we indirectly highlighted the area by darkening the rest of the image with a black overlay (opacity: 20%).

### 2.4. Consultation with Main Developers of MSKCC

After all interviews have been conducted, we discussed the requirements with the main developers of cBioPortal from the Memorial Sloan Kettering Cancer Center (MSKCC) in New York, USA, in an online audio conference. Prior to that, we had detailed the most important and far-reaching changes in a letter including excerpts from our mockups.

The aim of this was to increase the chances of merging our planned implementations into the main development branch of cBioPortal and to maintain contact with the main developers right from the beginning.

### 2.5. Ethical Approval

This study was ethically approved by the ethics committee of the Friedrich-Alexander-University Erlangen-Nürnberg (FAU) (see Supplementary File 6).

## 3. Results

### 3.1. Overview of Scoped Review

Based on our keyword search we selected 306 unique articles out of which 27 dealt with MTBs and were kept for further review. Next, two papers were discarded because their full texts were not available. From the remaining 25 publications, we excluded another 13 since they did not describe IT support in MTBs, which resulted in a total of twelve articles for our review. For details see Supplementary File 1.

### 3.2. Details about Interviewees

We conducted the first round of interviews with a total of 18 participants at nine different university hospitals to determine all requirements for an MTB software tool based on cBioPortal. Up to four participants were interviewed simultaneously at each site. Representatives of the following disciplines were involved: oncology (10), pathology (4), systems medicine/systems biology (2), bioinformatics (1) and human genetics (1).

The second round of interviews discussed and evaluated the requirements identified in the first round in detail with a total of 16 participants at the same university hospitals as above. Up to three participants were interviewed simultaneously at each site. Representatives of the following disciplines were present: oncology (7), pathology (4), systems medicine/systems biology (2), bioinformatics (1), human genetics (1) and urology (1).

### 3.3. Requirements from the First Round of Interviews and Screenshot Mockups

During the first round of interviews, a total of 49 requirements with up to seven potential options for implementation each were surveyed (see Supplementary File 7). Ten requirements either already implemented in cBioPortal or out of scope of our Use Case were dropped prior to the second round of interviews. This included features to:

- Highlight mutations with existing treatment options;
- Display information about general availability of a specific drug in Germany;
- Point out mutations causing treatment resistance;
- Mark germline mutations;
- Display variant allele frequencies alongside corresponding coverage;
- Integrate the database “Clinical Interpretations of Variants in Cancer” (CIViC) [19];
- Visualize mRNA expression data.

A platform to discuss individual mutations across hospitals, for example in the context of a forum, is outside the scope of MIRACUM’s Use Case 3 and was, therefore, not included in the second round of interviews either.

The choice of the file format to be used for the import of the mutation data into cBioPortal was made independently of the interviews by all use case members (MAF: “mutation annotation format”). In addition, a feature to permanently hide mutations in a specific sample in cBioPortal was denied since this filtering should be done by MIRACUM-Pipe [7] only.

In preparation for the second round of interviews, we created a total of 54 screenshot mockups demonstrating almost all surveyed requirements and their respective options (see Supplementary File 8).

### 3.4. Consolidated Requirements from the Second Round of Interviews

Below we provide a rough overview of the consolidated requirements we surveyed during the second round of interviews. For a list and detailed description of all final requirements and their respective options see Supplementary File 9.

#### 3.4.1. Improving Patient Case Analysis

Since case analysis in personalized medicine relies on various information such as details about the patient or—in case of MTBs—the underlying tumor, cBioPortal should provide those by integrating various information and knowledge sources in a single tool. Therefore, the participants requested, amongst others, clinical patient data to be stored in cBioPortal. Displaying sample metadata and their subsequent analysis was also requested. This includes, for example, the type and location of a biopsy as well as the exact specifications of the sequencer used.

**Figure 2:**
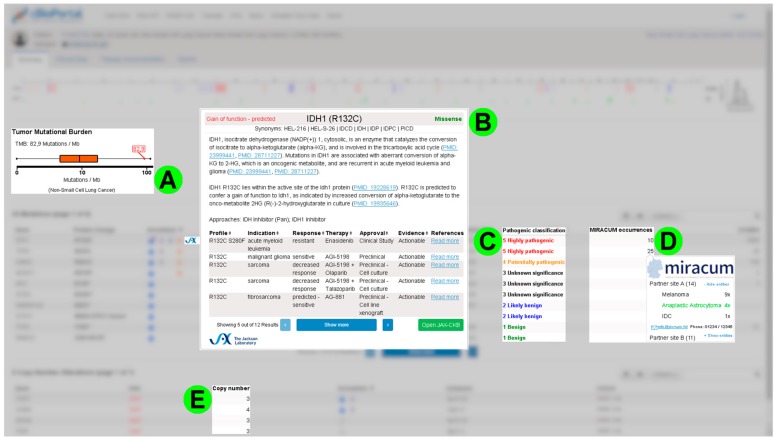
Collage demonstrating some requirements for the case analysis. This collage is a synthesis of different screenshot mockups, which exemplarily illustrates some requirements for individual case analysis: (**A**) A visual representation of the tumor mutational burden relative to the expected range of the corresponding tumor entity using a box plot. (**B**) Integration of data from the JAX Clinical Knowledgebase (JAX-CKB) via annotation and pop-up. (**C**) Classification of pathogenicity as an example of data extension in the mutation table of the patient view. (**D**) Tool for an entity-specific display of how often a certain mutation was found and classified as relevant for the therapy recommendation at the partner sites. If necessary, the contact data may be used to exchange experiences. (**E**) Extension of the table with copy number variation (CNV) data by the exact number of copies. Example data adopted from the public cBioPortal (https://cbioportal.org) and JAX-CKB knowledgebase [20].

Furthermore, cBioPortal seems to lack important (calculated) values for its usage in molecular tumor boards. Ranging from the tumor mutational burden (see Figure 2A) up to values that indicate the pathogenicity of individual mutations (see Figure 2C). Mutations are automatically annotated with the latter by the MIRACUM-Pipe [7] and this information should be displayed in cBioPortal. We also discovered potential improvements for already existing features in cBioPortal, like the visualization of copy number variations (see Figure 2E).

Since the interviewees also use numerous different databases when evaluating a patient case, the integration of additional knowledgebases is highly desired. In this context, the JAX Clinical Knowledgebase [20,21] was mentioned explicitly and considered to be beneficial when integrated (see Figure 2B).

Besides that, the visualization of molecular pathways can be an important tool that links individual mutations to molecular function and pathway in the search for a therapy option.

In order to improve cooperation, there were requests for a central service to (automatically) report a mutation that has led to a therapy recommendation before. If other participating hospitals similarly identify this mutation in one of their samples, it should be highlighted and contact details should be displayed (see Figure 2D) for detailed, personal exchange of expertise.

#### 3.4.2. Supporting the Development and Recording of a Therapy Recommendation

**Figure 3:**
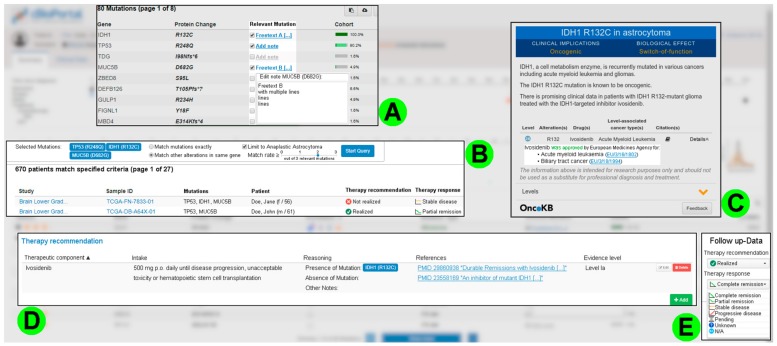
Collage demonstrating some requirements for the development and recording of a therapy recommendation. This collage depicts image sections from the screenshot mockups we created based on the original interface of cBioPortal: (**A**) Checkboxes and text fields in the mutation table of the patient view to mark potentially relevant mutations for the therapy recommendation. (**B**) Search functionality with automatic parameter transfer to find previous patient cases with a mutation pattern similar to that of the current patient. (**C**) Extension of OncoKB’s information to cover the European Medicines Agency’s (EMA) approval status of a given drug. (**D**) Summary of already entered components of the therapy recommendation for the current patient. (**E**) Option to record follow-up data for the current patient. Example data adopted from the public cBioPortal (https://cbioportal.org) and OncoKB [16].

Once the patient’s data has been reviewed, the members of the molecular tumor board determine—if possible—the potentially relevant mutations for a therapy recommendation based on their previous analysis. This selection should be recorded in cBioPortal (see Figure 3A) and serve as the basis of the following features.

This set of relevant mutations is used to search for similar patient cases that have been analyzed in the local hospital before. A search should be comprehensively parameterizable and values from the current patient case (e.g., tumor entity, relevant mutations, etc.) should be automatically applied (see Figure 3B). The interviewees considered the gathering of information related to therapy recommendations for previous patients as the main goal of this functionality. This requirement was complemented by the need to document follow-up data (see Figure 3E). Therefore, details on the progression status of similar patient cases can be reviewed and included in the evaluation of the current case more easily.

Building on this, further information on a possible therapy approach is required. In addition to the already available functions in cBioPortal provided by OncoKB [16], the interviewees requested a way to easily query the approval status of a drug in their respective country (in the case of MIRACUM: Germany) (see Figure 3C).

Since, according to the participants, some therapy components (e.g., a drug) are only available in the context of (pre-) clinical studies, integration of clinical trial databases such as ClinicalTrials.gov [22] were requested. The interviewees expect a more efficient search for suitable studies through the automatic transfer of relevant search parameters, which are taken from the cBioPortal data record.

Once a patient case has been prepared based on its individual data, it will be presented and discussed during a meeting of the MTB in order to jointly develop a therapy recommendation. There was no consensus as to what extent cBioPortal must support such a presentation. Some considered an automatically generated set of slides with individualized content as helpful. Others preferred to use the cBioPortal graphical user interface itself during the presentation with an option to hide irrelevant content. However, some interviewees also considered such assistance to be completely unnecessary.

After a therapy recommendation has been decided upon within the MTB, it must be recorded in detail in cBioPortal. Besides information on the therapy itself (e.g., the name of a drug), the molecular and clinical rationale for the recommendation needs to be documented, too. As a justification, the mutations earlier classified as relevant or the tumor mutational burden (TMB) may be referenced.

To submit the results of the MTB to the client (e.g., the treating physician), the interviewees requested a function to generate a PDF report. Besides the actual therapy recommendation, this report should also contain extracts from the consulted databases and thus also be used to archive the current state of knowledge that led to the decisions made.

#### 3.4.3. Requirements for IT Infrastructure

In order to integrate cBioPortal in the various clinical system landscapes, a standardized application programming interface (API) like FHIR (Fast Healthcare Interoperability Resources [23]) should be used by the respective components of the hospital information system to feed a local cBioPortal instance with various and comprehensive data (e.g., clinical data). In addition, the information, which is created or altered within cBioPortal, should be accessible via this interface for export into the local hospital information system.

The existing user system in cBioPortal must be extended by a comprehensive and flexible user and rights management. Of course, only authenticated and authorized users should be permitted to use the cBioPortal instance for an MTB at all. Furthermore, it should be possible to “freeze” a therapy recommendation made by the molecular tumor board and to allow subsequent changes only for certain users and only in a reproducible way. This implies that all alterations to the data records in cBioPortal can be traced, thus guaranteeing their integrity and authenticity.

In addition, some sites indicated that patient data may be stored in a pseudonymized manner if this becomes necessary for legal reasons (like it is done in the public available cBioPortal instance hosted by the Memorial Sloan Kettering Cancer Center). Of course, in this case, it must be guaranteed that the data can be reassigned to the patient at the end. For this purpose, an identification code assigned to the patient by the hospital information system could be used for pseudonymization.

## 4. Discussion

Due to the huge amount of data produced by NGS technologies, the growing number of clinical studies and released targeted therapies to address treatment of cancer, new tools are required to identify relevant molecular alterations and matching therapy options for individual patients. The present paper outlines which of those are essential and which functionalities exactly have to be provided to support processes in MTBs. To our knowledge, there is no comparable work so far tackling this issue from the point of view of participants in MTBs.

### 4.1. Results and Future Work

We worked out a requirements specification for the software supporting the processes in an MTB based on the open source project cBioPortal, an integrated database, which allows to visualize clinical parameters and molecular findings of individual patients in the context of current knowledge about cancer diseases. It turned out that the screenshot mockups we had created prior to the second round of interviews played an important role in the further requirements analysis. They allowed us to describe the different requirements and their potential options identified in the first round of interviews briefly and succinctly and thus to quickly start a discussion in the second round. They may also serve as blueprints for the future implementation of the requirements as it was the case with the integration of the Genome Aggregation Database (gnomAD [24]), where our mockups served as inspiration for the core cBioPortal development [25].

This conversation with the participants turned out to be the most important part of this work at all, as on the one hand questions that occurred could be resolved immediately and on the other hand, requirements that had not yet been considered by neither the interviewees nor us came to light by intensive discussions. Therefore, we could finally identify 24 requirements that were not yet integrated into cBioPortal at the time of the survey. Some of them have been proposed to the community of cBioPortal with requests for comments (RFC) filed by other users or even have been implemented before the finalization of this paper [25,26,27,28,29,30,31,32]. RFCs are publicly available documents everyone can create with proposals for new features in cBioPortal that often also include descriptions of a potential way to implement these. They are an important step in the development of new functionalities in cBioPortal and help to involve the community in the process of implementation. This demonstrates, how important those requirements—even outside of an MTB context—seem to be for the future of cBioPortal.

Besides that, some of our requirements identified are also met by tools not directly associated with cBioPortal, like MatchMiner [33] published by the Dana–Farber Cancer Institute. This tool, which has recently been prototypically integrated in cBioPortal [34], offers a service to match clinical trials to a patient case. At the moment, all studies have to be cured manually, so to use this feature to prepare MTBs in Germany, the ongoing challenge of an automatic import from databases like ClinicalTrials.gov [22] still remains to be resolved.

For the future we plan to integrate cBioPortal in the hospital information systems (HIS) of our partner sites. This means the cBioPortal instance imports clinical data related to patients that have been registered for the MTB directly from the corresponding electronic health record (EHR). This data comprises at least age, gender and tumor entity and ideally also recent diagnoses and therapy attempts as well as data from cancer registries. The sequencing data is automatically processed by the MIRACUM-Pipe [7] under parameter control, including alignment, variant calling, annotation and analyses, in order to finally be passed directly to the cBioPortal instance.

In addition to the import of existing data into cBioPortal, users also need to add persistent information to patient cases in cBioPortal to mark relevant mutations in a patient’s sample or to finally document the therapy recommendation. Since we are in ongoing contact with the main developers of cBioPortal, who finally determine which new features are merged into their project and provide future maintenance of them, we identified a considerable conflict with this requirement: cBioPortal’s primary focus is to support research in form of a read-only data warehouse. Therefore, the main developers stressed, that having direct write access by the user is currently not intended. A solution for this problem would be to place a hyperlink in cBioPortal to an input mask not hosted in cBioPortal itself, where the user can enter the data (e.g., the therapy recommendation). This form forwards the data to the patient’s EHR in the HIS via a standardized application programming interface (API) like FHIR [23], which is supported by most modern systems of an EHR (e.g., MEONA [35]). In order to make this data available again in cBioPortal, an (automatic) import must take place on a regular basis (e.g., twice daily). Thus, the data warehouse concept of cBioPortal would remain and no user write access in cBioPortal itself is necessary.

We also discussed another problem we came across: the rigid process to import mutation data into cBioPortal. At the moment, there is no way to (dynamically) import additional annotation data on individual mutations without fundamental changes to the backend, which would be necessary for the integration of the demanded scores and similar. The team in Use Case 3 of the MIRACUM consortium responsible for the implementation already took the first steps and submitted a request for comments to the community to address this problem. This request deals with a flexible integration of further mutation data by means of an additional database column with the data stored in JavaScript object notation (JSON) format [36].

In general, it is necessary to store data in a structured manner rather than in free text in order to achieve a high grade of automation during import. For example, the International Classification of Diseases for Oncology (ICD-O) can be used to encode the tumor entity, the German Federal Ministry of Health has stated in its announcement [37]. The use of a standardized ontology, such as that provided by OncoTree [38], can also be useful.

However, not only technical hurdles have to be tackled, but also legal ones. For example, when integrating further databases, particular attention must be paid to licensing as some restrict use to research purposes only. For example, even though the JAX Clinical Knowledgebase (JAX-CKB) was developed “to support clinical decision-making” [21], in the disclaimer of their website they allow usage “only for research and educational purposes” [20]. Use Case 3 of the MIRACUM consortium currently uses cBioPortal in clinical research. However, if it is used outside of a research context in the future, of course, this and also existing laws and regulations must be taken into account during the development, as well. Besides compliance with data protection regulations [39,40] and the Medical Devices Act [41], this is a very broad field.

As for future works, the implementation of the collected requirements must address these problems and find viable ways in close coordination with the main developers and the community around cBioPortal. This is the only way to integrate the features into the project permanently and to ensure their further maintenance and support.

### 4.2. Related Work

We came across two related works taking place in Germany. Halfmann et al. report that they developed a tool that aims to support the preparation process of a molecular tumor board as well as the presentation of a patient case during a meeting [12]. A video published by them demonstrates how different tools, including cBioPortal, can be combined in a single user interface [42]. Among other requirements, they discovered, like we did, that a function “to search for comparable local cases” [12] is demanded by clinical experts.

Fegeler et al. also describe a software solution for the support of molecular tumor boards. In addition to the option of planning and managing the processes in an MTB, they also describe an integrated video conferencing system. They plan to integrate cBioPortal and knowledge databases, to support the development of a therapy recommendation [13].

### 4.3. Limitations

Since we collected the requirements based on the expertise of clinicians who could only spend a certain amount of time for the survey and interviews (timeframe ranged between 40 min and two hours per interview and per round), we concentrated on the requirements which had not yet been implemented in cBioPortal. Therefore, we cannot make any statements about already existing features of the software that are useful for an MTB. As we iteratively improved and extended the interview guideline in the first round, the second round of interviews for consolidation had to be conducted so that in the end all sites had the opportunity to comment on every single requirement.

We also limited the number of sites interviewed to nine university hospitals spread all over Germany. Furthermore, not all disciplines and their representatives involved in a molecular tumor board were interviewed. Although we tried to incorporate as many disciplines as possible, this may not be a representative sample for Germany.

As a by-product of our requirements analysis and in preparation for the interviews, we performed a scoped review to provide an overview of already existing tools and systems that support molecular tumor boards. A limitation of it is its methodological rigor as compared to a full systematic review. We limited our database search to the MEDLINE and Web of Science databases. Therefore, we might have neglected relevant articles neither listed in MEDLINE nor in Web of Science. In addition, only one author performed the review, so this might also reduce the quality of results since misinterpretations cannot be systematically excluded. However, we believe that for our purposes we achieved a high degree of methodological quality throughout this scoping review by following the PRISMA statement [14,15] as far as appropriate for the requirements analysis.

## 5. Conclusions

By interviewing experts at our partner sites, we identified and consolidated for the first time a list of requirements for IT-supported preparation of molecular tumor boards based on cBioPortal. This list comprises a total of 24 requirements that had not yet been implemented during the time of the interviews. For almost all of them and their subordinated features, we have created descriptive screenshot mockups (54 in total) which supported the interview process and may contribute to the further development of cBioPortal. This work provides important information based on the clinical needs that will ultimately support the members of an MTB interpret the complex data for a personalized therapy recommendation.

## Supplementary Materials

Supplementary materials can be found at https://www.mdpi.com/2075-4418/10/2/93/s1.

## Abbreviations

API	Application programming interface
cBioPortal	cBio Cancer Genomics Portal
CIViC	Clinical Interpretation of Variants in Cancer (knowledgebase)
CNV	Copy Number Variation
EHR	Electronic Health Record
EMA	European Medicines Agency
FHIR	Fast Healthcare Interoperability Resources
GIMP	GNU Image Manipulation Program
gnomAD	Genome Aggregation Database
HIS	Hospital Information System
IT	Information technology
JAX-CKB	Jackson Laboratory Clinical Knowledgebase
JSON	JavaScript Object Notation
MIRACUM	Medical Informatics in Research and Care in University Medicine
MSKCC	Memorial Sloan Kettering Cancer Center
MTB	Molecular Tumor Board
NGS	Next-generation sequencing
PRISMA	Preferred Reporting Items for Systematic Reviews and Meta-Analysis
RFC	Request for Comments
TMB	Tumor Mutational Burden

## References

[1] Parker B.A., Schwaederlé M., Scur M.D., Boles S.G., Helsten T., Subramanian R., Schwab R.B., Kurzrock R. (2015). Breast Cancer Experience of the Molecular Tumor Board at the University of California, San Diego Moores Cancer Center. J. Oncol. Pract..

[2] Bryce A.H., Egan J.B., Borad M.J., Stewart A.K., Nowakowski G.S., Chanan-Khan A., Patnaik M.M., Ansell S.M., Banck M.S., Robinson S.I. (2017). Experience with precision genomics and tumor board, indicates frequent target identification, but barriers to delivery. Oncotarget.

[3] Hoefflin R., Geißler A.-L., Fritsch R., Claus R., Wehrle J., Metzger P., Reiser M., Mehmed L., Fauth L., Heiland D.H. (2018). Personalized Clinical Decision Making Through Implementation of a Molecular Tumor Board: A German Single-Center Experience. JCO Precis. Oncol..

[4] Perera-Bel J., Hutter B., Heining C., Bleckmann A., Fröhlich M., Fröhling S., Glimm H., Brors B., Beißbarth T. (2018). From somatic variants towards precision oncology: Evidence-driven reporting of treatment options in molecular tumor boards. Genome Med..

[5] Hinderer M., Boerries M., Haller F., Wagner S., Sollfrank S., Acker T., Prokosch H.-U., Christoph J. (2017). Supporting Molecular Tumor Boards in Molecular-Guided Decision-Making - The Current Status of Five German University Hospitals. Stud. Health Technol. Inform..

[6] Prokosch H.-U., Acker T., Bernarding J., Binder H., Boeker M., Boerries M., Daumke P., Ganslandt T., Hesser J., Höning G. (2018). MIRACUM: Medical Informatics in Research and Care in University Medicine. Methods Inf. Med..

[7] Metzger P., Scheible R., Hess M., Boeker M., Andrieux G., Börries P. MIRACUM-Pipe (AG-Boerries/MIRACUM-Pipe Repository). https://github.com/AG-Boerries/MIRACUM-Pipe.

[8] Gao J., Aksoy B.A., Dogrusoz U., Dresdner G., Gross B., Sumer S.O., Sun Y., Jacobsen A., Sinha R., Larsson E. (2013). Integrative analysis of complex cancer genomics and clinical profiles using the cBioPortal. Sci. Signal..

[9] Cerami E., Gao J., Dogrusoz U., Gross B.E., Sumer S.O., Aksoy B.A., Jacobsen A., Byrne C.J., Heuer M.L., Larsson E. (2012). The cBio cancer genomics portal: an open platform for exploring multidimensional cancer genomics data. Cancer Discov..

[10] Shneiderman B., Jantke K.P. (2001). Inventing Discovery Tools: Combining Information Visualization with Data Mining. Discovery science, Proceedings of the 4th International Conference, DS 2001, Washington, DC, USA, 25–28 November 2001.

[11] Omta W.A., de Nobel J., Klumperman J., Egan D.A., Spruit M.R., Brinkhuis M.J.S. (2017). Improving Comprehension Efficiency of High Content Screening Data Through Interactive Visualizations. Assay Drug Dev. Technol..

[12] Halfmann M., Stenzhorn H., Gerjets P., Kohlbacher O., Oestermeier U., Auer S., Vidal M.-E. (2019). User-Driven Development of a Novel Molecular Tumor Board Support Tool. Data Integration in the Life Sciences.

[13] Fegeler C., Zsebedits D., Bochum S., Finkeisen D., Martens U.M. (2018). Implementierung eines IT-gestützten molekularen Tumorboards in der Regelversorgung. FORUM.

[14] Moher D., Liberati A., Tetzlaff J., Altman D.G. (2009). Preferred reporting items for systematic reviews and meta-analyses: the PRISMA statement. PLoS Med..

[15] Liberati A., Altman D.G., Tetzlaff J., Mulrow C., Gøtzsche P.C., Ioannidis J.P.A., Clarke M., Devereaux P.J., Kleijnen J., Moher D. (2009). The PRISMA statement for reporting systematic reviews and meta-analyses of studies that evaluate health care interventions: explanation and elaboration. PLoS Med..

[16] Chakravarty D., Gao J., Phillips S.M., Kundra R., Zhang H., Wang J., Rudolph J.E., Yaeger R., Soumerai T., Nissan M.H. (2017). OncoKB: A Precision Oncology Knowledge Base. JCO Precis. Oncol..

[17] SOPHiA GENETICS. https://www.sophiagenetics.com/.

[18] Dubovenko A., Nikolsky Y., Rakhmatulin E., Nikolskaya T. (2017). Functional Analysis of OMICs Data and Small Molecule Compounds in an Integrated "Knowledge-Based" Platform. Methods Mol. Biol..

[19] Griffith M., Spies N.C., Krysiak K., McMichael J.F., Coffman A.C., Danos A.M., Ainscough B.J., Ramirez C.A., Rieke D.T., Kujan L. (2017). CIViC is a community knowledgebase for expert crowdsourcing the clinical interpretation of variants in cancer. Nat. Genet..

[20] The Jackson Laboratory JAX Clinical Knowledgebase - Disclaimer. https://ckb.jax.org/about/disclaimer.

[21] Patterson S.E., Liu R., Statz C.M., Durkin D., Lakshminarayana A., Mockus S.M. (2016). The clinical trial landscape in oncology and connectivity of somatic mutational profiles to targeted therapies. Hum. Genomics.

[22] U.S. National Library of Medicine ClinicalTrials.gov. https://clinicaltrials.gov/.

[23] HL7 Welcome to FHIR. https://www.hl7.org/fhir/.

[24] Karczewski K.J., Francioli L.C., Tiao G., Cummings B.B., Alföldi J., Wang Q., Collins R.L., Laricchia K.M., Ganna A., Birnbaum D.P. (2019). Variation across 141,456 human exomes and genomes reveals the spectrum of loss-of-function intolerance across human protein-coding genes. bioRxiv.

[25] GitHub User Leexgh. https://github.com/cBioPortal/cbioportal-frontend/pull/2064.

[26] GitHub User Leexgh. https://github.com/cBioPortal/cbioportal-frontend/pull/2502.

[27] GitHub User Leexgh. https://github.com/cBioPortal/cbioportal-frontend/pull/2596.

[28] GitHub User Jjgao. https://github.com/cBioPortal/cbioportal/issues/6444.

[29] GitHub User Pvannierop. https://github.com/cBioPortal/cbioportal-frontend/pull/2053.

[30] GitHub User Pvannierop. https://github.com/cBioPortal/cbioportal-frontend/pull/2055.

[31] GitHub User Kjgao. https://github.com/cBioPortal/cbioportal/issues/6446.

[32] Lukasse P., van Hagen S. RFC45: Gene Panel Information in Patient View. https://docs.google.com/document/d/1X7dA_wJFtv5xJO1oHCSt8DUdTmk07RexvHUpjCJsSM4/edit.

[33] Lindsay J., Fitz C.D.V., Zwiesler Z., Kumari P., van der Veen B., Monrose T., Mazor T., Barry S., Albayrak A., Tung M. MatchMiner: An Open Source Computational Platform for Real-Time Matching of Cancer Patients to Precision Medicine Clinical Trials Using Genomic and Clinical Criteria. https://www.biorxiv.org/content/10.1101/199489v3.

[34] GitHub User Victoria34. https://github.com/cBioPortal/cbioportal/pull/5679.

[35] MEONA GmbH Meona. https://www.meona.de/.

[36] Unberath P. RFC50: Add Support for Additional Arbitrary Variant Annotation. https://docs.google.com/document/d/1Pybk4_-lrirKJZ_cH64riZBRdWXdkJnCQqzx1O2fjRo/edit#heading=h.oyj1ec8k7lgx.

[37] German Federal Ministry of Health Updated Standardized Oncological Basic Data Set of the Consortium of German Tumor Centers e.V. (ADT) and the Society of Epidemiological Cancer Registries in Germany e.V. (GEKID) (“Aktualisierter Einheitlicher Onkologischer Basisdatensatz der Arbeitsgemeinschaft Deutscher Tumorzentren e.V. (ADT) und der Gesellschaft der Epidemiologischen Krebsregister in Deutschland e.V. (GEKID)”). https://www.bundesanzeiger.de/.

[38] OncoTree. https://github.com/cBioPortal/oncotree.

[39] German Federal Ministry of Justice and Consumer Protection German Federal Data Protection Act (“Bundesdatenschutzgesetz / BDSG”). https://www.gesetze-im-internet.de/englisch_bdsg/englisch_bdsg.pdf.

[40] (2016). REGULATION (EU) 2016/679 OF THE EUROPEAN PARLIAMENT AND OF THE COUNCIL - of 27 April 2016 - On the Protection of Natural Persons with Regard to the Processing of Personal Data and On the Free Movement of Such Data, and Repealing Directive 95/46/EC (General Data Protection Regulation).

[41] German Federal Ministry of Justice and Consumer Protection German Medical Devices Act (“Gesetz über Medizinprodukte - MPG”). https://www.gesetze-im-internet.de/mpg/.

[42] YouTube-User PersOnS Interface Prototype Demo—YouTube. https://www.youtube.com/watch?v=VXD3Rap11zg.

