# Supplementary material for: Requirements Analysis and Specification for a Molecular Tumor Board Platform Based on cBioPortal
Source: Diagnostics (Basel). 2020 Feb 10;10(2):93. doi: 10.3390/diagnostics10020093 (PMC7167859; doi:10.3390/diagnostics10020093)
Supplement: Supplementary file 1 [file diagnostics-10-00093-s001.zip › Supplementary File 1_Methods and results of scoping review.pdf]

Supplementary File 1: Methods and results of scoping review.

Methods of scoping review

Although the scoped review was only intended to generate questions and topics for the subsequent first round of interviews, we tried to follow the Preferred Reporting Items for Systematic Reviews and Meta-Analysis (PRISMA) guidelines [1,2] as far as appropriate for this scoping review. Furthermore, a study protocol was written prior to the investigation but has not been registered.

We searched MEDLINE and Web of Science (all databases) for IT systems and tools supporting MTBs. The search included the three main aspects “molecular tumor boards”, “decision support” and “information science" covered by the search query as shown in Table A1. The final search was conducted on September 12, 2017. The inclusion criteria for the scoping review were as follows: English article; manuscript in a peer-reviewed journal; research article or review paper, involving an MTB, describing IT support for MTBs, full-text is available or can be requested.

Table A1. Search queries by databases.

Database	Search query
MEDLINE	(tumor board) AND ((molecular) OR (genetic*) OR (genomic*) OR (pharmacogenetic*) OR (pharmacogenomic*)) AND ((decision support) OR (decision making) OR (clinical decision-making) OR (information science))
Web of Science	TS=((tumor board) AND ((molecular) OR (genetic*) OR (genomic*) OR (pharmacogenetic*) OR (pharmacogenomic*)) AND ((decision support) OR (decision making) OR (clinical decision-making) OR (information science)))

MH screened the titles, index terms, and abstracts for all identified publications to decide if all inclusion criteria were met. In cases where no clear decision could be made on the basis of this information, the full texts of the articles were screened in order to make a decision about inclusion or exclusion.

For all publications meeting the inclusion criteria listed above, the following data items were extracted:

1. article type,
2. setting,
3. objective,
4. IT support used or provided for,
    a. case preparation prior to the MTB review,
    b. case review during MTB review and
    c. for results communication of MTB review.

For data extraction, article types were defined as review paper if IT support for MTB was discussed and reviewed within the article, but no original research was conducted. Research article was defined as any kind of original research including IT support for MTBs. For example, we did not distinguish between an article simply describing or evaluating software developments for MTBs and manuscripts of clinicians reporting on case series of MTBs. However, to provide an overview of the studies included, we summarized both the setting and the objective of each included manuscript. This included the study time, location and institution as well as a description of the project and its funding. The category “IT support used or provided for” comprised IT tools and software components which were manually used by clinicians or other health care providers (such as databases searched by clinicians, for instance); semi-automatically implemented (such as preliminary variant calling and annotations which need to

be reviewed by a bioinformatician, for instance) or fully automated (such as computer-generated clinical reports for results communication of the MTB).

The risk of bias for individual studies was not systematically assessed.

We either cited the text verbatim or summarized it. The categories "article type", "setting" and "objective" were supposed to provide a brief but sufficient overview of the study context of each included article. Whereas, the last category "IT support used or provided for" and its three sub-categories "case preparation prior to the MTB review", "case review during MTB review", and "final results communication of MTB review" was specifying the IT tools and systems for the support of MTBs.

The articles were summarized in the form of tables and narrative discussion. Furthermore, we identified and analyzed IT support used or provided for

a) case preparation prior to the MTB review,
b) case review during MTB review and
c) for results communication of MTB review.

In order to classify and rank the identified and analyzed IT support, the following two levels of IT support (Level 1 and Level 2) were defined:

Level 1 (articles which fulfill at least one of the following two criteria):

- solely describing the manual review of publicly or commercially available IT tools or databases (e.g. for manual variant annotation)
- the bioinformatics pipeline described in the article only provides automated IT support from data sequencing up to variant calling

Level 2 (articles which meet at least one of the following two criteria):

- the bioinformatics pipeline described in the article provides automated IT support beyond variant calling (e.g. automated variant annotation)
- other automated IT support for members of the MTBs (e.g. automatically generated report containing drug recommendations).

Each article was assigned to one of the two IT support levels. Therefore, we used the citation and the results of the tabulated attributes of the second domain.

Results and Discussion of scoping review

The databases searches identified 337 potentially relevant articles with 246 articles found in MEDLINE and 91 articles identified in Web of Science respectively. After 31 duplicates were removed, 306 potentially relevant articles remained. During the title and abstract review, 279 articles were excluded for not involving an MTB. It means, an MTB was neither mentioned nor described in the title or abstract. We could obtain full-texts for 25 of the remaining 27 articles, whereas 2 articles were excluded because the full-text was neither available via institutional library access nor through the authors' user profile on the ResearchGate platform. The remaining 25 articles underwent full-text review, after which 13 articles were excluded for not describing IT support for MTBs. After all, 12 articles were finally included for review (see Figure A1).

Figure A1. Flow diagram of article selection.

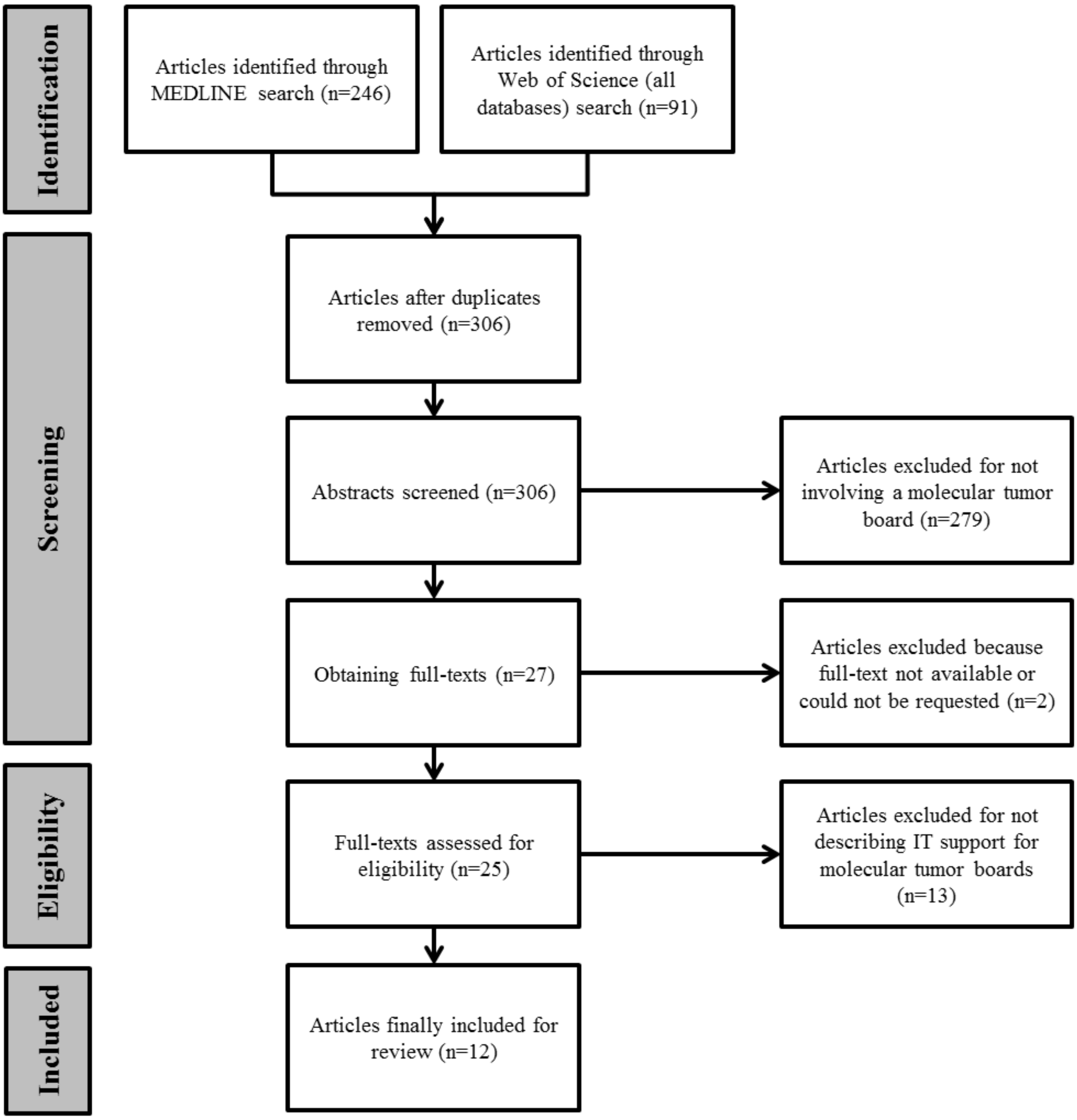

In accordance with our level of IT support classification, we categorized 8 articles as providing Level 1 IT support and 4 articles as providing Level 2 IT support respectively. A tabulated and brief but sufficient overview of the study context of each included article is provided below (see Table A2). Depending on which abstraction method was most appropriate for an individual article, we either summarized or simply copied out relevant parts of the articles.

Table A2. Overview of articles finally included for review. RP = review paper, RA = research article.

Level	Citation	Article type	Setting	Objective
1	Holch et al. 2017 [3]	RP	Description of developments leading to identification and application of potential biomarkers using Universal Genomic Testing (UGT). On this basis, the authors review the clinical evidence of this approach and summarize recommendations for the ongoing evaluation of UGT as the next step in oncological decision-making.	Illustrating the current perspective on the importance of genetic testing and MTBs for precision medicine in general.
	Bardia et al. 2016 [4]	RA	Case report of a patient with metastatic breast cancer with an ESR1 mutation. The case was discussed at a Massachusetts General Hospital Molecular and Precision Medicine (MAP) tumor board meeting in 2015.	Reviewing the general approach for interpretation of genotyping results, the clinical significance of the specific mutation in the particular cancer, potential strategies to target the pathway, and implications for clinical practice.
	Tafe et al. 2016 [5]	RA	DNA from tumor specimens was sequenced in a CLIA-certified laboratory to identify coding mutations in a 50-gene panel (n= 534) or a 255-gene panel (n= 51). Cases were evaluated by a multidisciplinary MTB at the Dartmouth-Hitchcock Medical Center.	Providing the framework of the MTB at the Dartmouth-Hitchcock Medical Center, their format for case evaluation, a summary of 1 year of cases and their experience with anticipated obstacles.
	Everett et al. 2014 [6]	RA	Genetic counselors (GCs) discuss options for return of results with patients during the informed consent process and document family histories. GCs also review germline findings and actively participate in the multidisciplinary Precision Medicine Tumor Board (PMTB), providing clinical context for interpretation of germline results and making recommendations about disclosure of germline findings.	Describing the experiences and the roles of GCs as part of a research team implementing a protocol for whole genome sequencing (WGS) of tumours and paired germline DNA known as the Michigan Oncology Sequencing project (MI-ONCOSEQ).
	Roycho-wdhury et al. 2011 [7]	RA	Patients with advanced or refractory cancer were enrolled in a trial (MI-ONCOSEQ). For each patient, WES of the tumor, targeted whole-exome sequencing of tumor and normal DNA, and transcriptome sequencing (RNA-Seq) of the tumor was performed to identify	Exploring practical challenges of applying high-throughput sequencing in clinical oncology.

			potentially informative mutations in a clinically relevant time frame of 3 to 4 weeks. A multidisciplinary Sequencing Tumor Board (STB) deliberated on the clinical interpretation of the obtained sequencing results.	
	Lane et al. 2015 [8]	RA	Specimens were analysed for approx. 2800 mutations in 50 genes. Outcomes of interest included tumor sequencing advisory board (TSAB) function and processes, timely discussion of results, and proportion of reports having potentially actionable mutations were presented at a biweekly TSAB.	Outlining the roles, function, and interaction of a multidisciplinary TSAB.
	Hinderer et al. 2017 [9]	RA	Semi-structured interviews with experts of five university hospitals between December 2016 and February 2017 were conducted.	Describing the organizational structure and procedures which are currently supporting the MTBs of five German university hospitals.
	Oberg et al. 2016 [10]	RA	Reviewing the results of the first 101 patients in the precision cancer medicine program. WES of patient-matched tumor-normal samples and RNA-Seq of tumor was performed to identify sequence variants, fusion transcripts, relative gene expression, and copy number variation (CNV). Results were initially reviewed by a molecular pathologist and subsequently by a multi-disciplinary MTB. Clinical reports were issued to the ordering physician and posted to the patient's electronic medical record.	1) Reporting the experience with integrating clinical next-generation sequencing (NGS) into paediatric haematology-oncology practice using the PIPseq pipeline; and 2) describing the broad clinical utility of genomically informed cancer medicine.
2	Beltran et al. 2015 [11]	RA	Trial for patients with metastatic or treatment-resistant disease using a WES clinical test called ExaCT-1 during a 19-month period (February 2013 through September 2014). A comprehensive computational pipeline capable of categorizing mutations (as category 1, 2, or 3 on the basis of actionability) and generating a clinical report for discussion in a multidisciplinary PMTB and clinical follow-up. Patients were observed for 7 to 25 months for	Understanding how WES data affect therapeutic decision making in patients with advanced cancer and to identify novel biomarkers of response.

			correlation of molecular information with clinical response.	
	Meißner et al. 2015 [12]	RA	A software for supporting MTB as part of an Omics Pipe tool or as a standalone reporting tool is presented. The software is called OncoRep, an RNA-Seq based n-of-1 reporting tool for breast cancer patients, which is developed within the open-source software environments R (v3.0.2) and Bioconductor (v2.13).	Presenting a software development to address several major challenges of precision medicine.
	Saulnier Sholler et al. 2015 [13]	RA	In this study, patients undergoing tumor biopsy have a sample sent for pathological evaluation and gene expression profiling from which bioinformatics analysis and generation of a drug prediction report is created. This is reviewed by an MTB which yields an individualized treatment plan for each patient, who is then followed for safety and response.	1) Evaluating the feasibility of a process which would utilize genome-wide mRNA expression data of bone-marrow-derived neuroblastoma cells or tumor biopsies to support individualized treatment decisions; 2) evaluating the safety of allowing a MTB to determine individualized treatment plans; and 3) determining the activity of treatments chosen based on overall response rate (ORR) and progression free survival (PFS)
	LoRusso et al. 2015 [14]	RA	A nontreatment pilot study utilizing NGS technologies, including whole genome and whole transcriptome sequencing, to identify molecular aberrations in patients with non–V600 BRAF metastatic melanoma. This information was then rationally matched to an appropriate clinical treatment from a defined pharmacopeia. Five patients with advanced non–V600 BRAF metastatic melanoma were enrolled.	Mirroring most aspects of a subsequent large randomized study which is currently being conducted.

A tabulated and comprehensive comparison of the IT tools and systems for the support of MTBs of each included article is provided below (see Table A3). Depending on which abstraction method was most appropriate for an individual article, we either summarized or simply copied out relevant parts of the articles

Table A3. IT support used or provided for MTBs.

Level	Citation	IT support used or provided for		
		case preparation prior to MTB review	case review during MTB review	final results communication of MTB review
1	Holch et al. 2017 [3]	Proposed selected databases for: 1. variant calling (1.1) germline variants:	-	-

		ExAC (Exome Aggregation Consortium); IGSR (The International Genome Sample Resource); NHLBI Exome Sequencing Project; dbSNP (The Single Nucleotide Polymorphism Database); 1.2) somatic variants: COSMIC (Catalogue of Somatic Mutations in Cancer); NIH Genomic Data Commons (GDC) Data Portal; cBioPortal for Cancer Genomics; IntOGen (Integrative OncoGenomics)); and 2. variant annotation and assessment/classification of actionability (CIViC (Clinical Interpretation of Variants in Cancer); JAX-CKB (The Jackson Laboratory Clinical Knowledgebase); MD Anderson Knowledge Base for Precision Oncology; My Cancer Genome)		
	Bardia et al. 2016 [4]	The report of the patient's genotyping results from tissue and blood are visualized in JBrowse	-	-
	Tafe et al. 2016 [5]	Each case was assigned to a member of the MTB to review publicly available tools and databases to determine or evaluate: 1. frequency of given mutation in large patient populations (e.g., cBio Cancer Genomics Portal); 2. (a) whether a given mutation was previously observed and evaluated, (b) potential for germline mutation, (c) pathway analysis, (d) available drugs (approved, off-label, or experimental) targeting the affected pathway(s), and (e) the level of evidence (i.e., preclinical in vitro [cell-free vs. cell	-	-

		culture], preclinical in vivo, clinical case report, clinical trial, and phase supporting a mutation-induced change in protein function and/or drug sensitivity (at the protein, pathway, cellular, or tumoral level) (e.g., PubMed, COSMIC, Google, MutationAssessor, UniProt, ClinVar, and dbSNP)		
	Everett et al. 2014 [6]	It is mentioned that a computational omics pipeline exists, e.g. for routine annotation of germline variants in a list of 160 genes in recognized cancer pathways. However, no particular IT tool or database is mentioned. Furthermore, pedigrees and GC interpretation and clinical comments are entered into a shared data portal (no further description of this shared data portal provided). GC's and a molecular geneticist review the assayed germline findings in the context of medical and family history, and research publicly available mutation databases and primary literature for relevant clinical information and pathogenic classification (no particular databases mentioned again). The results are presented in the MTB by the GC.	-	-
	Roychowdhury et al. 2011 [7]	Set of bioinformatics pipelines for data sequencing, alignment and variant calling (Genome Analysis Toolkit (GATK), BWA and in-house algorithms), e.g. detecting somatic mutations, CNV, structural variations, gene fusions, and highly	-	-

		overexpressed genes. Databases (COSMIC, dbSNP, HapMap3 and the Human Gene Mutation Database (HGMD) were used as IT support for variant annotation. The final results are tabulated for presentation to the STB.		
	Lane et al. 2015 [8]	Set of bioinformatics pipelines for data sequencing, alignment and variant calling (in-house Ion Torrent Variant Caller using COSMIC mutations), e.g. detecting somatic mutations, pathway analysis. The final results are presented to the TSAB.	-	-
	Hinderer et al. 2017 [9]	Set of bioinformatics pipeline, including the databases COSMIC, ClinVar, and dbSNP resources. The databases were mainly used for results analysis and annotation. The annotated somatic gene variants and mutations for the final interpretation were organized in a Microsoft Excel spreadsheet. In four hospitals the report is electronically communicated to the treating physician and stored as a PDF document in the EHR for the MTB.	-	-
	Oberg et al. 2016 [10]	Set of bioinformatics pipeline (PIPSeq) for variant calling (NextGene software using COSMIC database), CNV, fusion transcripts, relative gene expression, pathway analysis etc. Therefore, they generated a model from transcriptomes in their database, allowing them to identify expression outliers. For each patient, a report was issued containing	-	-

		variant calls, CNV, fusions, and overexpressed genes. Variants were assigned a tier based on disease-association and separately a tier based on level of evidence for clinical actionability. Reports were delivered to ordering oncologists and posted to the electronic medical record (EMR).		
2	Beltran et al. 2015 [11]	A custom computational pipeline (IPM-Exome-pipeline, version 0.9) with alignment, normalization (R), rigorous quality control, mutation calling and annotation was implemented for each case-control pair for simultaneous detection of somatic single-nucleotide variation (e.g. using an in-house SNV caller (SNVseeqer) or COSMIC for somatic mutations and querying dbSNP for potential germline mutations), indels, and CNV, including actionable and cancer mutation prioritization (category 1: actionable and clinically relevant genes with FDA approved drugs, 2: cancer-associated genes that represent targets for therapies in clinical or preclinical development or are considered mutational drivers and potentially actionable; or 3: all other somatic alterations of unknown clinical or biologic significance). Furthermore, publicly available tools and databases to determine frequency of given mutation in large patient populations. Finally, a	-	Clinical report (EXaCT-1 sequencing report) in PDF format is uploaded into the patient's EMR, including 1. clinical information (e.g. disease type, site of biopsy, and tumor content), 2. case images (e.g. photomicrographs of tumor histology), 3. genetic alterations and 4. automated lists of category I-III alterations (and possible drug recommendations, if applicable) with references and clinical trial information. Additional data sharing with clinicians and team members occurs through an internal web-based cBioPortal for data visualization, a BAM file viewing interface using IGV and integration with EMR systems.

		clinical report was generated and additional data shared with clinicians and team members through cBioPortal for data visualization and a BAM file viewing interface using the Integrative Genomics Viewer IGV and integration with EMR systems.		
	Meißner et al. 2015 [12]	OncoRep was integrated as an RNA-Seq Cancer Report pipeline in Omics Pipe, which handles the processing of the raw RNA-Seq data in an automated and parallel manner on a compute cluster, e.g. for quality control, alignment, gene expression quantification and differential expression, gene fusion identification, pathway analysis and variant calling (SNPiR). After the data were processed, the results files from each step and the patient specific meta data were automatically processed by OncoRep to produce a summary report for each patient. OncoRep performs the following analyses: i) variant annotation (SnpEff, dbNSFP, COSMIC, ClinVar, CADD, DrugBank, PharmGKB and IntOGen); ii) gene expression estimation; iii) differential gene expression analysis (DESeq2); iv) pathway analysis (SPIA); v) prediction of receptor status and molecular subtype; and vi) selection of drugs targeting dysregulated genes, variants and pathways (by drawing on information provided by DrugBank, KEGG	The HTML report produces interactive tables that are sortable and searchable. They can be exported as CSV files to be viewed in spreadsheet software. Gene descriptors and drugs are linked to the respective databases for easy access to further information. Pathways are visualized and they are annotated with differentially expressed genes	-

		Drug and PharmGKB). OncoRep displays these results along with the results from the quality control of the raw data and alignment, variant calling, fusion gene detection and estimation of oncogenic potential. The R package knitr is used to produce an interactive HTML report. A PDF file containing a final summary report is generated using the R package Sweave.		
	Saulnier Sholler et al. 2015 [13]	Generating a drug prediction report by using microarray expression data from patient tumours which are compared to a series of normal biological controls. The normal reference set was a whole-body bank of 45 normal tissue gene expression levels which were used as the reference set for the normalization calculations. Data were submitted to a database of algorithms designed to predict relevant medications. These algorithms included biomarker rules, drug target expression (using a variety of public and commercial knowledge bases including DrugBank, PharmGKB, GeneGo-Thomson Reuters, UptoDate, MedTrack and DrugDex), network-based methods, drug response, and drug sensitivity signatures. The results were then presented in a report to the MTB. For all drugs with predicted efficacy, an associated predicted efficacy score and rank was provided in the report.	-	-
	LoRusso et al. 2015 [14]	Set of bioinformatics pipeline for variant calling, CNV, fusion transcripts, structural	-	-

		events, relative gene expression, pathway analysis etc. (no particular databases mentioned). The study team curated a drug rule database as a screening tool comprising genomic alterations which were previously linked to a therapy of the study pharmacopeia (including both FDA-approved and investigational agents). Each drug in the pharmacopeia was annotated by domain experts with information on how specific genes and alterations in those genes may influence drug response from evidence in published literature sources, such as: gene, genomic alteration such as variant, drug, direction of association (sensitive, resistant), publication link, and evidence text of the relationship. If a patient's gene matched a gene in the database, then the aberration type was checked. If a match occurred, an annotated rule statement was triggered and presented in table form stating the relationship and indication for the drug. The generated report also included additional evidence tables for triggered rules and included outbound hyperlinks to the original rule evidence source. An algorithmic method for ranking rules was not implemented, as that was considered to be the purpose of the tumor board.		

The group of Level 1 IT support includes one article simply proposing a selection of publicly or commercially available databases for manual review and search which could be incorporated into a bioinformatics pipeline [3]. As opposed to that, five articles described the available databases which they used for manual review of their genetic results [5,7–10]. In another article, a set of bioinformatics pipeline up to the variant calling was mentioned but no further information on the specific IT tools or databases used was provided [6]. Bardia et al. implemented a commercial software for the visualization of the report of the patient's genotyping results from tissue and blood. However, we could not determine whether the commercial software is generally used for the review of the genetic test results of the CLIA-certified laboratory or just for the presented case report [4].

All four articles providing Level 2 IT support included a bioinformatics pipeline that goes beyond Level 1 IT support and results in a clinical report for the MTB review. In one article, the pipeline included prioritization of actionable and cancer mutation (categories 1-3) that were included in the clinical report. This report was generated as a PDF file. Furthermore, additional data were shared with clinicians and team members through cBioPortal for data visualization and a BAM file viewing interface using the Integrative Genomics Viewer (IGV) and integration with EMR systems [11]. In the study by Meißner et al., the results were automatically processed by the IT tool OncoRep to produce a summary report for each patient. Therefore, R packages were implemented and used to generate both an interactive HTML report and a PDF file [12]. According to Saulnier Sholler et al., their pipeline results were submitted to a database of algorithms designed to predict relevant medications for the generation of a drug prediction report [13]. The authors of the fourth article curated a drug rule database as a screening tool and a pharmacopeia (including both FDA-approved and investigational agents) with annotated drugs. The annotation of the pharmacopeia drugs was performed by biomedical experts of the study. The clinical report did not only contain drug recommendations but also additional evidence tables for triggered rules and outbound hyperlinks to the original rule evidence source [14].

Furthermore, we found COSMIC, ClinVar, and dbSNP to be amongst the most cited and searched databases of the included articles [3,5,7–10,12]. These databases were mainly used for the preparation of relevant cases prior to the actual MTB review in both levels of IT support (Level 1 and Level 2). Furthermore, databases for the selection of appropriate drugs such as PharmGKB and DrugBank, for instance, were found to be useful for Level 2 IT support in two articles by Meißner et al. and Saulnier Sholler et al. [12,13]. In both of them, the integration of such databases resulted in automated drug recommendations as part of a final clinical report for the MTB review. Therefore, Saulnier Sholler et al. submitted their results data to a database of algorithms designed to predict relevant medications. These algorithms included biomarker rules, drug target expression, network-based methods, drug response, and drug sensitivity signatures. For all drugs with predicted efficacy, an associated predicted efficacy score and rank was provided in the report. Nevertheless, the format of the drug prediction report (e.g. PDF, HTML, etc.) is not described. Furthermore, the authors provide no information on the results communication (e.g. storing the drug prediction report in the patient's EMR) [13].

Only one included article provided IT support for the MTB review in the form of both an HTML report producing interactive tables that are sortable and searchable during the MTB review and a PDF clinical report. Moreover, these data are machine-readable and can be exported as CSV files to be viewed in spreadsheet software and incorporated into the final clinical report. Furthermore, the HTML report provided the members of the MTB with links to gene descriptors and drugs for further information. The software claims to be suitable for supporting MTB as part of an Omics Pipe tool or as a standalone reporting tool. However, the article does not describe or include an actual MTB. It also does not present any information regarding the feasibility or usability of OncoRep. Both would be crucial to determine the usefulness of this tool. Furthermore, we could not determine whether the HTML-based

or PDF clinical reports are finally stored within the EMR after the review of the MTB. It is only mentioned that a PDF version is sent to the treating physicians [12]. Nevertheless, bioinformatics pipelines generating machine-readable data are already in place in most clinical environments [6–14].

We identified one article that provided an in-depth overview of their IT support for the communication of the results of the MTB. This IT support consists of two main parts: First, a comprehensive clinical report (EXaCT-1 sequencing report) that was uploaded and stored as a PDF file in the patient's EMR. This makes the results accessible for treating physicians and can be added to the final medical report for patient admission, for instance. Second, the treating physicians are enabled to gather further through both an internal web-based cBioPortal for data visualization and a BAM file viewing interface using an integrative genomics viewer [11]. Despite all this, the authors do not describe whether the initial clinical report generated by the computational pipeline is modified during the MTB review (e.g. interpretations and therapy options filled in) or whether this report is already final prior to the MTB review and thus stored in the patient's EMR without modifications.

In our opinion, another promising approach of IT support for MTB was described by LoRusso et al.. The article provides an in-depth overview of IT support for the preparation of the MTB. The study team curated a drug rule database comprising drugs of the study pharmacopeia (including both FDA-approved and investigational agents). Each drug in the pharmacopeia was annotated by domain experts of the study which is a crucial factor for user’s acceptance [14]. The database automatically checked for applicable drugs for an individual patient and drug matches were incorporated into the clinical report. This report also included additional evidence tables for triggered rules and included outbound hyperlinks to the original rule evidence. This is another crucial factor for users’ acceptance since it is an evidence-based approach revealing the indication for a particular selected drug [14]. Unfortunately, the article does not provide any details regarding the format of the results report (e.g. PDF, HTML, etc.). It solely mentions that a summary of the subsequent clinical tumor board and treatment plan was written [14].

References

1. Moher, D.; Liberati, A.; Tetzlaff, J.; Altman, D.G. Preferred reporting items for systematic reviews and meta-analyses: the PRISMA statement. *PLoS Med.* **2009**, *6*, e1000097:1-e1000097:6, doi:10.1371/journal.pmed.1000097.

2. Liberati, A.; Altman, D.G.; Tetzlaff, J.; Mulrow, C.; Gøtzsche, P.C.; Ioannidis, J.P.A.; Clarke, M.; Devereaux, P.J.; Kleijnen, J.; Moher, D. The PRISMA statement for reporting systematic reviews and meta-analyses of studies that evaluate health care interventions: explanation and elaboration. *PLoS Med.* **2009**, *6*, e1000100:1-e1000100:28, doi:10.1371/journal.pmed.1000100.

3. Holch, J.W.; Metzeler, K.H.; Jung, A.; Riedmann, K.; Jost, P.J.; Weichert, W.; Kirchner, T.; Heinemann, V.; Westphalen, C.B. Universal Genomic Testing: The next step in oncological decision-making or a dead end street? *Eur. J. Cancer* **2017**, *82*, 72–79, doi:10.1016/j.ejca.2017.05.034.

4. Bardia, A.; Iafrate, J.A.; Sundaresan, T.; Younger, J.; Nardi, V. Metastatic Breast Cancer With ESR1 Mutation: Clinical Management Considerations From the Molecular and Precision Medicine (MAP) Tumor Board at Massachusetts General Hospital. *Oncologist* **2016**, *21*, 1035–1040, doi:10.1634/theoncologist.2016-0240.

5. Tafe, L.J.; Gorlov, I.P.; Abreu, F.B. de; Lefferts, J.A.; Liu, X.; Pettus, J.R.; Marotti, J.D.; Bloch, K.J.; Memoli, V.A.; Suriawinata, A.A.; et al. Implementation of a Molecular Tumor Board: The Impact

on Treatment Decisions for 35 Patients Evaluated at Dartmouth-Hitchcock Medical Center. *Oncologist* **2015**, *20*, 1011–1018, doi:10.1634/theoncologist.2015-0097.

6. Everett, J.N.; Gustafson, S.L.; Raymond, V.M. Traditional roles in a non-traditional setting: genetic counseling in precision oncology. *J. Genet. Couns.* **2014**, *23*, 655–660, doi:10.1007/s10897-014-9698-3.
7. Roychowdhury, S.; Iyer, M.K.; Robinson, D.R.; Lonigro, R.J.; Wu, Y.-M.; Cao, X.; Kalyana-Sundaram, S.; Sam, L.; Balbin, O.A.; Quist, M.J.; et al. Personalized oncology through integrative high-throughput sequencing: a pilot study. *Sci. Transl. Med.* **2011**, *3*, 111ra121:1-111ra121:12, doi:10.1126/scitranslmed.3003161.
8. Lane, B.R.; Bissonnette, J.; Waldherr, T.; Ritz-Holland, D.; Chesla, D.; Cottingham, S.L.; Alberta, S.; Liu, C.; Thompson, A.B.; Graveel, C.; et al. Development of a Center for Personalized Cancer Care at a Regional Cancer Center: Feasibility Trial of an Institutional Tumor Sequencing Advisory Board. *J. Mol. Diagn.* **2015**, *17*, 695–704, doi:10.1016/j.jmoldx.2015.07.003.
9. Hinderer, M.; Boerries, M.; Haller, F.; Wagner, S.; Sollfrank, S.; Acker, T.; Prokosch, H.-U.; Christoph, J. Supporting Molecular Tumor Boards in Molecular-Guided Decision-Making - The Current Status of Five German University Hospitals. *Stud. Health Technol. Inform.* **2017**, *236*, 48–54, doi:10.3233/978-1-61499-759-7-48.
10. Oberg, J.A.; Glade Bender, J.L.; Sulis, M.L.; Pendrick, D.; Sireci, A.N.; Hsiao, S.J.; Turk, A.T.; Dela Cruz, F.S.; Hibshoosh, H.; Remotti, H.; et al. Implementation of next generation sequencing into pediatric hematology-oncology practice: moving beyond actionable alterations. *Genome Med.* **2016**, *8*, 133:1-133:19, doi:10.1186/s13073-016-0389-6.
11. Beltran, H.; Eng, K.; Mosquera, J.M.; Sigaras, A.; Romanel, A.; Rennert, H.; Kossai, M.; Pauli, C.; Faltas, B.; Fontugne, J.; et al. Whole-Exome Sequencing of Metastatic Cancer and Biomarkers of Treatment Response. *JAMA Oncol.* **2015**, *1*, 466–474, doi:10.1001/jamaoncol.2015.1313.
12. Meißner, T.; Fisch, K.M.; Gioia, L.; Su, A.I. OncoRep: an n-of-1 reporting tool to support genome-guided treatment for breast cancer patients using RNA-sequencing. *BMC Med. Genomics* **2015**, *8*, 24:1-24:8, doi:10.1186/s12920-015-0095-z.
13. Saulnier Sholler, G.L.; Bond, J.P.; Bergendahl, G.; Dutta, A.; Dragon, J.; Neville, K.; Ferguson, W.; Roberts, W.; Eslin, D.; Kraveka, J.; et al. Feasibility of implementing molecular-guided therapy for the treatment of patients with relapsed or refractory neuroblastoma. *Cancer Med.* **2015**, *4*, 871–886, doi:10.1002/cam4.436.
14. LoRusso, P.M.; Boerner, S.A.; Pilat, M.J.; Forman, K.M.; Zuccaro, C.Y.; Kiefer, J.A.; Liang, W.S.; Hunsberger, S.; Redman, B.G.; Markovic, S.N.; et al. Pilot Trial of Selecting Molecularly Guided Therapy for Patients with Non-V600 BRAF-Mutant Metastatic Melanoma: Experience of the SU2C/MRA Melanoma Dream Team. *Mol. Cancer Ther.* **2015**, *14*, 1962–1971, doi:10.1158/1535-7163.MCT-15-0153.
