# Supplementary material for: Requirements Analysis and Specification for a Molecular Tumor Board Platform Based on cBioPortal
Source: Diagnostics (Basel). 2020 Feb 10;10(2):93. doi: 10.3390/diagnostics10020093 (PMC7167859; doi:10.3390/diagnostics10020093)
Supplement: Supplementary file 1 [file diagnostics-10-00093-s001.zip › Supplementary File 2_Guideline with questions for first round of interviews.pdf]

Supplementary File 2: Guideline with questions for first round of interviews.

We conducted the interviews in German. This is a translation of the guideline we created and used in the first round of interviews.

1. Is there already a tool like cBioPortal in use to aid in the preparation of the MTB?
2. Which tools for annotation are used at the site? Which do you know, but do not use?
3. Which databases do you frequently consult to prepare a case for the MTB?
4. Is there a way to support the search for literature?
5. How and where do you search for suitable studies?
6. How do you present your results to the molecular tumor board?
7. Where do you enter the therapy recommendation?
8. How are the MTB recommendations passed on to the clinical client?
    a. How do you link the realized therapy to the initial therapy recommendation?
9. Do you archive the therapy recommendation also in your local HIS (or somewhere else)?
10. Which file format is used at the site to store data from sequencing?
11. Where do your archive sequencing data?
12. In addition to the sequencing data, the clinical report and the therapy recommendation, are there other "intermediate formats" stored (e.g., as PDF/XML/DB elements)?
13. How long do you keep those archived data?
14. cBioPortal & OncoKB & other tools
    a. Presentation (see Supplementary Files 3 and 4)
    b. Which of the shown features may be useful?
    c. What improvements need to be made to use those tools in preparation for an MTB?
    d. What features do you miss?
    e. Which clinical data must be displayed on the platform?
