# Supplementary material for: Requirements Analysis and Specification for a Molecular Tumor Board Platform Based on cBioPortal
Source: Diagnostics (Basel). 2020 Feb 10;10(2):93. doi: 10.3390/diagnostics10020093 (PMC7167859; doi:10.3390/diagnostics10020093)
Supplement: Supplementary file 1 [file diagnostics-10-00093-s001.zip › Supplementary File 3_Introduction of cBioPortal.pdf]

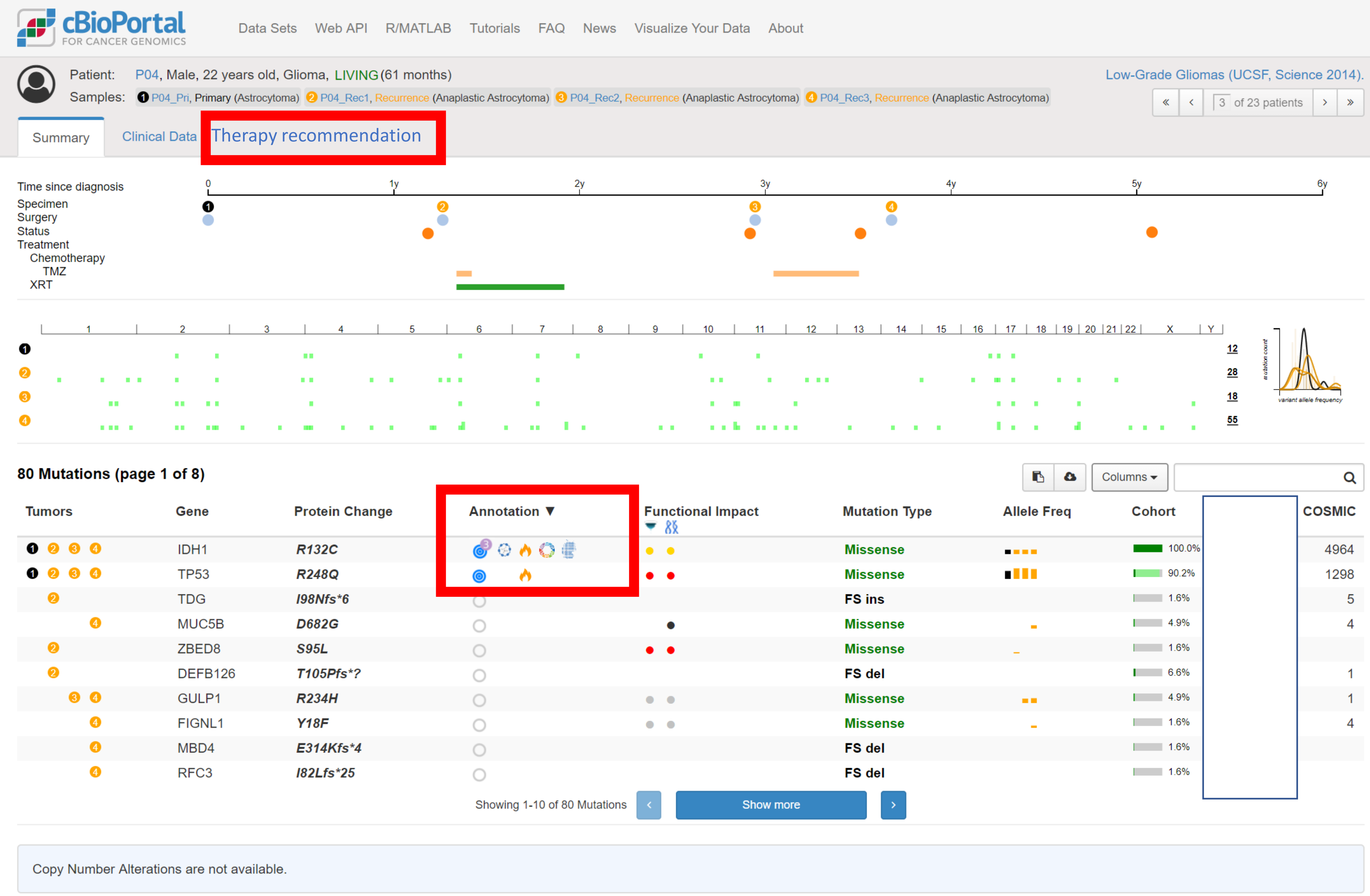
cBioPortal FOR CANCER GENOMICS
Data Sets
Web API
R/MATLAB
Tutorials
FAQ
News
Visualize Your Data
About
Patient: P04, Male, 22 years old, Glioma, LIVING (61 months)
Samples: 1 P04_Pri, Primary (Astrocytoma) 2 P04_Rec1, Recurrence (Anaplastic Astrocytoma) 3 P04_Rec2, Recurrence (Anaplastic Astrocytoma) 4 P04_Rec3, Recurrence (Anaplastic Astrocytoma)
Low-Grade Gliomas (UCSF, Science 2014).
3 of 23 patients
Summary
Clinical Data
Therapy recommendation
Time since diagnosis
0
1y
2y
3y
4y
5y
6y
Specimen
Surgery
Status
Treatment
Chemotherapy
TMZ
XRT
12
28
18
55
mutation count
variant allele frequency
80 Mutations (page 1 of 8)
Columns
Tumors
Gene
Protein Change
Annotation
Functional Impact
Mutation Type
Allele Freq
Cohort
COSMIC
IDH1 R132C Missense 100.0% 4964
TP53 R248Q Missense 90.2% 1298
TDG I98Nfs*6 FS ins 1.6% 5
MUC5B D682G Missense 4.9% 4
ZBED8 S95L Missense 1.6%
DEFB126 T105Pfs*? FS del 6.6% 1
GULP1 R234H Missense 4.9% 1
FIGNL1 Y18F Missense 1.6% 4
MBD4 E314Kfs*4 FS del 1.6%
RFC3 I82Lfs*25 FS del 1.6%
Showing 1-10 of 80 Mutations
Show more
Copy Number Alterations are not available.

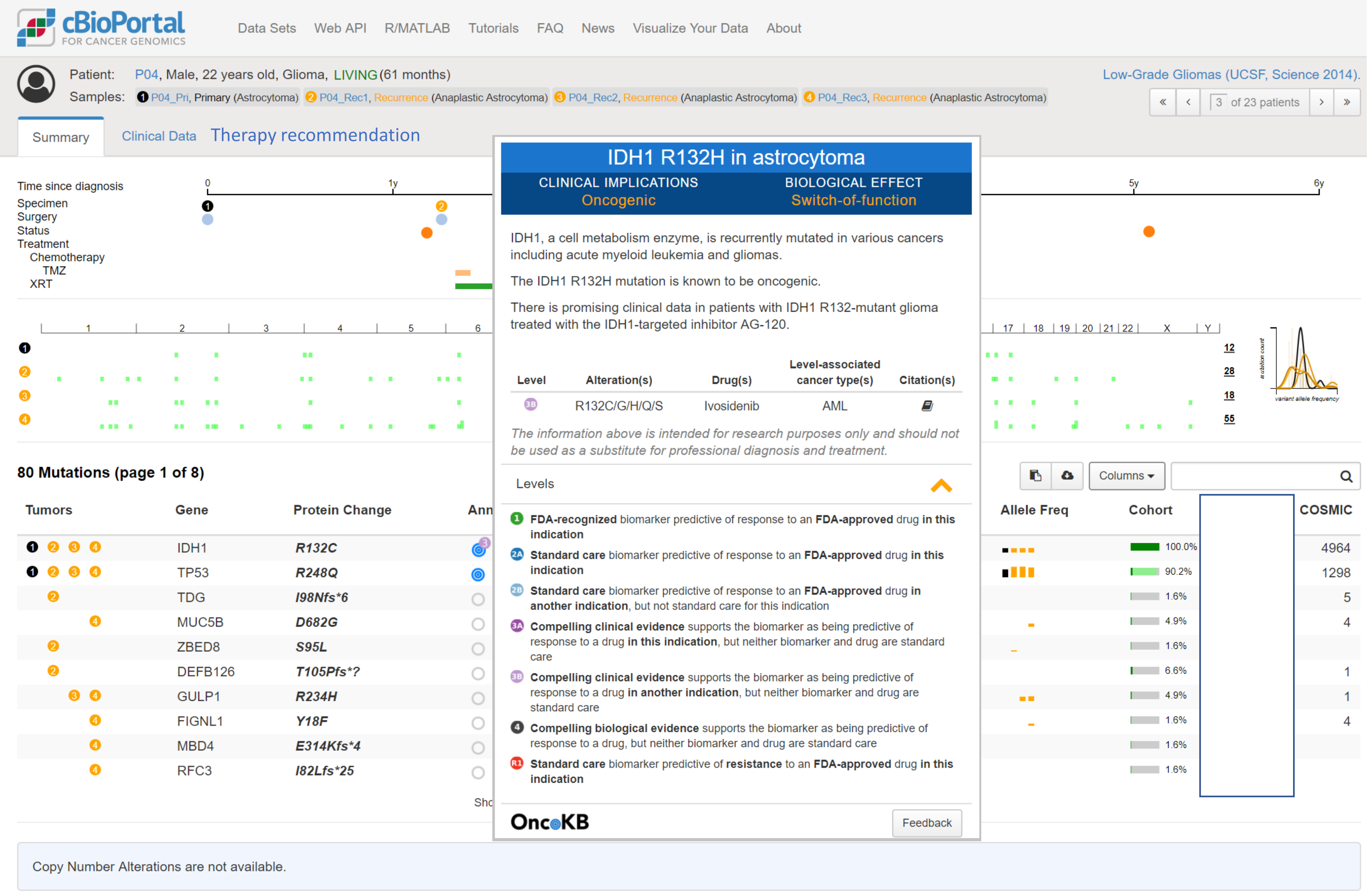
cBioPortal
FOR CANCER GENOMICS
Data Sets
Web API
R/MATLAB
Tutorials
FAQ
News
Visualize Your Data
About
Patient: P04, Male, 22 years old, Glioma, LIVING (61 months)
Low-Grade Gliomas (UCSF, Science 2014).
Samples: 1 P04_Pri, Primary (Astrocytoma) 2 P04_Rec1, Recurrence (Anaplastic Astrocytoma) 3 P04_Rec2, Recurrence (Anaplastic Astrocytoma) 4 P04_Rec3, Recurrence (Anaplastic Astrocytoma)
3 of 23 patients
Summary
Clinical Data
Therapy recommendation
Time since diagnosis
Specimen
Surgery
Status
Treatment
Chemotherapy
TMZ
XRT
IDH1 R132H in astrocytoma
CLINICAL IMPLICATIONS
Oncogenic
BIOLOGICAL EFFECT
Switch-of-function
IDH1, a cell metabolism enzyme, is recurrently mutated in various cancers including acute myeloid leukemia and gliomas.
The IDH1 R132H mutation is known to be oncogenic.
There is promising clinical data in patients with IDH1 R132-mutant glioma treated with the IDH1-targeted inhibitor AG-120.
Level | Alteration(s) | Drug(s) | Level-associated cancer type(s) | Citation(s)
3B | R132C/G/H/Q/S | Ivosidenib | AML
The information above is intended for research purposes only and should not be used as a substitute for professional diagnosis and treatment.
Levels
1 FDA-recognized biomarker predictive of response to an FDA-approved drug in this indication
2A Standard care biomarker predictive of response to an FDA-approved drug in this indication
2B Standard care biomarker predictive of response to an FDA-approved drug in another indication, but not standard care for this indication
3A Compelling clinical evidence supports the biomarker as being predictive of response to a drug in this indication, but neither biomarker and drug are standard care
3B Compelling clinical evidence supports the biomarker as being predictive of response to a drug in another indication, but neither biomarker and drug are standard care
4 Compelling biological evidence supports the biomarker as being predictive of response to a drug, but neither biomarker and drug are standard care
R1 Standard care biomarker predictive of resistance to an FDA-approved drug in this indication
OncoKB
Feedback
80 Mutations (page 1 of 8)
Columns
Tumors | Gene | Protein Change | Ann | Allele Freq | Cohort | COSMIC
IDH1 | R132C | 100.0% | 4964
TP53 | R248Q | 90.2% | 1298
TDG | I98Nfs*6 | 1.6% | 5
MUC5B | D682G | 4.9% | 4
ZBED8 | S95L | 1.6%
DEFB126 | T105Pfs*? | 6.6% | 1
GULP1 | R234H | 4.9% | 1
FIGNL1 | Y18F | 1.6% | 4
MBD4 | E314Kfs*4 | 1.6%
RFC3 | I82Lfs*25 | 1.6%
Copy Number Alterations are not available.

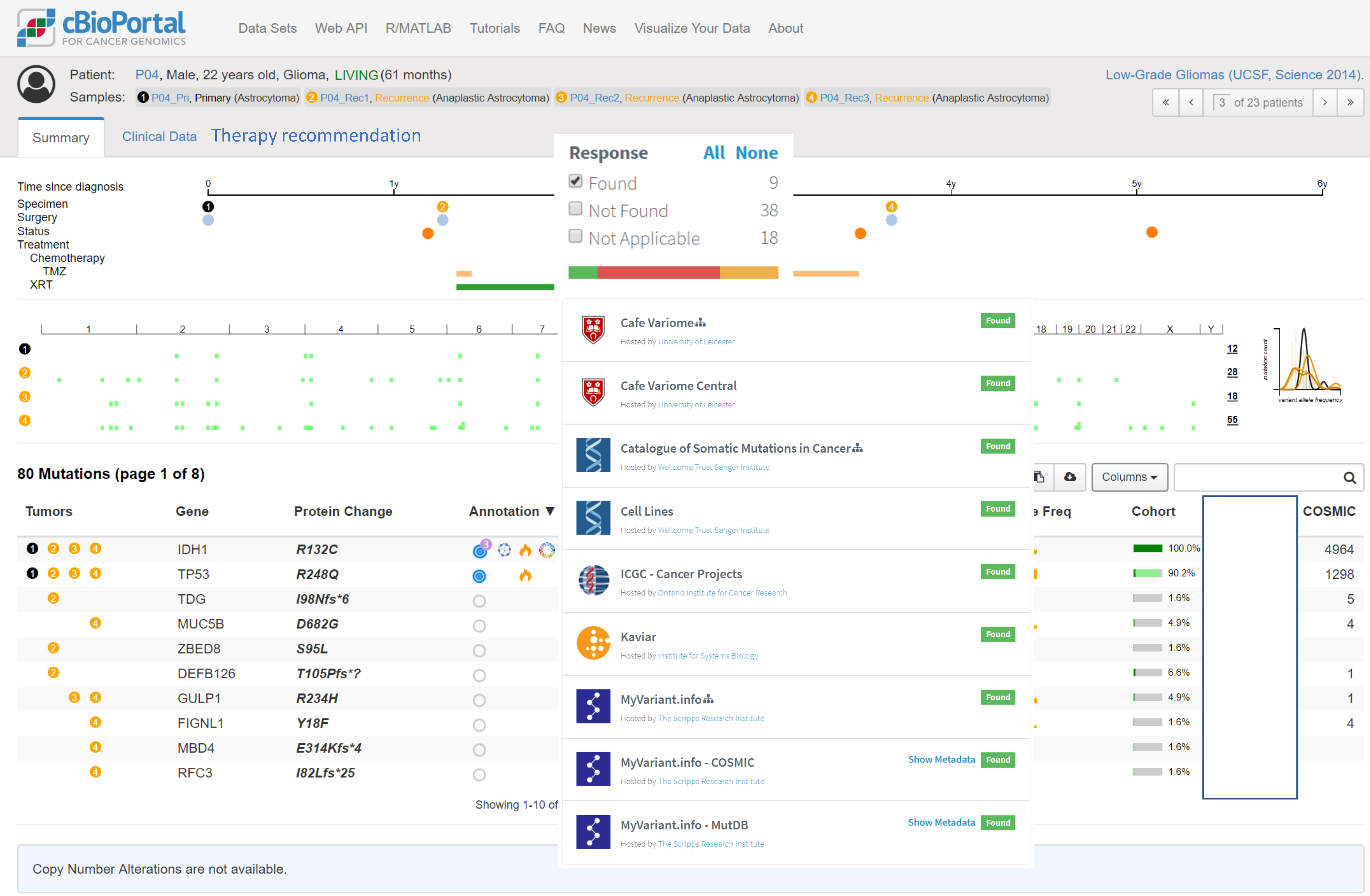
cBioPortal FOR CANCER GENOMICS
Data Sets Web API R/MATLAB Tutorials FAQ News Visualize Your Data About
Patient: P04, Male, 22 years old, Glioma, LIVING (61 months)
Low-Grade Gliomas (UCSF, Science 2014).
Samples: P04_Pri, Primary (Astrocytoma) P04_Rec1, Recurrence (Anaplastic Astrocytoma) P04_Rec2, Recurrence (Anaplastic Astrocytoma) P04_Rec3, Recurrence (Anaplastic Astrocytoma)
3 of 23 patients
Summary
Clinical Data
Therapy recommendation
Time since diagnosis
Specimen
Surgery
Status
Treatment
Chemotherapy
TMZ
XRT
Response All None
Found 9
Not Found 38
Not Applicable 18
Cafe Variome
Hosted by University of Leicester
Found
Cafe Variome Central
Hosted by University of Leicester
Found
Catalogue of Somatic Mutations in Cancer
Hosted by Wellcome Trust Sanger Institute
Found
Cell Lines
Hosted by Wellcome Trust Sanger Institute
Found
ICGC - Cancer Projects
Hosted by Ontario Institute for Cancer Research
Found
Kaviar
Hosted by Institute for Systems Biology
Found
MyVariant.info
Hosted by The Scripps Research Institute
Found
MyVariant.info - COSMIC
Hosted by The Scripps Research Institute
Show Metadata Found
MyVariant.info - MutDB
Hosted by The Scripps Research Institute
Show Metadata Found
80 Mutations (page 1 of 8)
Columns
Tumors Gene Protein Change Annotation Freq Cohort COSMIC
IDH1 R132C 100.0% 4964
TP53 R248Q 90.2% 1298
TDG I98Nfs*6 1.6% 5
MUC5B D682G 4.9% 4
ZBED8 S95L 1.6%
DEFB126 T105Pfs*? 6.6% 1
GULP1 R234H 4.9% 1
FIGNL1 Y18F 1.6% 4
MBD4 E314Kfs*4 1.6%
RFC3 I82Lfs*25 1.6%
Showing 1-10 of
variant allele frequency
Copy Number Alterations are not available.

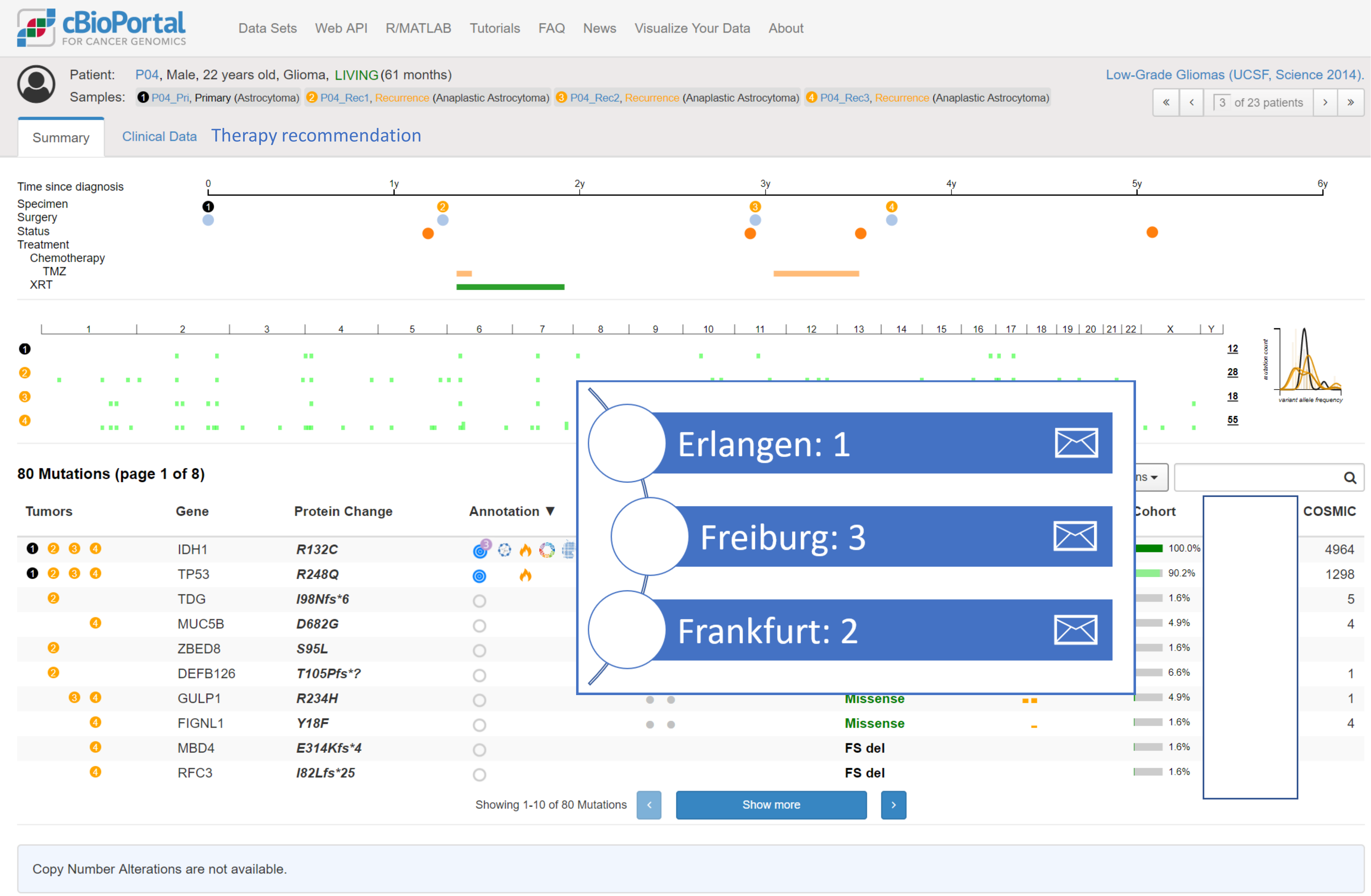
cBioPortal FOR CANCER GENOMICS
Data Sets
Web API
R/MATLAB
Tutorials
FAQ
News
Visualize Your Data
About
Patient: P04, Male, 22 years old, Glioma, LIVING (61 months)
Low-Grade Gliomas (UCSF, Science 2014).
Samples: 1 P04_Pri, Primary (Astrocytoma) 2 P04_Rec1, Recurrence (Anaplastic Astrocytoma) 3 P04_Rec2, Recurrence (Anaplastic Astrocytoma) 4 P04_Rec3, Recurrence (Anaplastic Astrocytoma)
3 of 23 patients
Summary
Clinical Data
Therapy recommendation
Time since diagnosis
Specimen
Surgery
Status
Treatment
Chemotherapy
TMZ
XRT
0
1y
2y
3y
4y
5y
6y
12
28
18
55
mutation count
variant allele frequency
Erlangen: 1
Freiburg: 3
Frankfurt: 2
80 Mutations (page 1 of 8)
Tumors
Gene
Protein Change
Annotation
Cohort
COSMIC
IDH1 R132C 100.0% 4964
TP53 R248Q 90.2% 1298
TDG I98Nfs*6 1.6% 5
MUC5B D682G 4.9% 4
ZBED8 S95L 1.6%
DEFB126 T105Pfs*? 6.6% 1
GULP1 R234H Missense 4.9% 1
FIGNL1 Y18F Missense 1.6% 4
MBD4 E314Kfs*4 FS del 1.6%
RFC3 I82Lfs*25 FS del 1.6%
Showing 1-10 of 80 Mutations
Show more
Copy Number Alterations are not available.

cBioPortal FOR CANCER GENOMICS

Data Sets | Web API | R/MATLAB | Tutorials | FAQ | News | Visualize Your Data | About

Patient: P04, Male, 22 years old, Glioma, LIVING (61 months)

Samples: 1 P04_Pri, Primary (Astrocytoma) 2 P04_Rec1, Recurrence (Anaplastic Astrocytoma) 3 P04_Rec2, Recurrence (Anaplastic Astrocytoma) 4 P04_Rec3, Recurrence (Anaplastic Astrocytoma)

Low-Grade Gliomas (UCSF, Science 2014).

« ‹ 3 of 23 patients › »

Summary | Clinical Data | Therapy recommendation

Date: 04/12/2018:
Reasoning: Mutations in IDH1 and TDG
Therapy recommendation: Ivosidenib
Comment:

Or:

„ Patient (m/22) with GIST, Result WES:
Lorem ipsum…“

80 Mutations (page 1 of 8)

Columns

Tumors	Gene	Protein Change	Annotation ▼	Functional Impact	Mutation Type	Allele Freq	Cohort	Locally used as Biomarker	COSMIC
1 2 3 4	IDH1	*R132C*			Missense		10[illegible]%	☑	4964
1 2 3 4	TP53	*R248Q*			Missense		90 %	☐	1298
2	TDG	*I98Nfs*6*			FS ins		1.6	☑	5
4	MUC5B	*D682G*			Missense		4.9	☐	4
2	ZBED8	*S95L*			Missense		1.6	☐	
2	DEFB126	*T105Pfs*?*			FS del		6.6	☐	1
3 4	GULP1	*R234H*			Missense		4.9	☐	1
4	FIGNL1	*Y18F*			Missense		1.6	☐	4
4	MBD4	*E314Kfs*4*			FS del		1.6	☐	
4	RFC3	*I82Lfs*25*			FS del		1.6	☐	

Showing 1-10 of 80 Mutations ‹ Show more ›

Copy Number Alterations are not available.

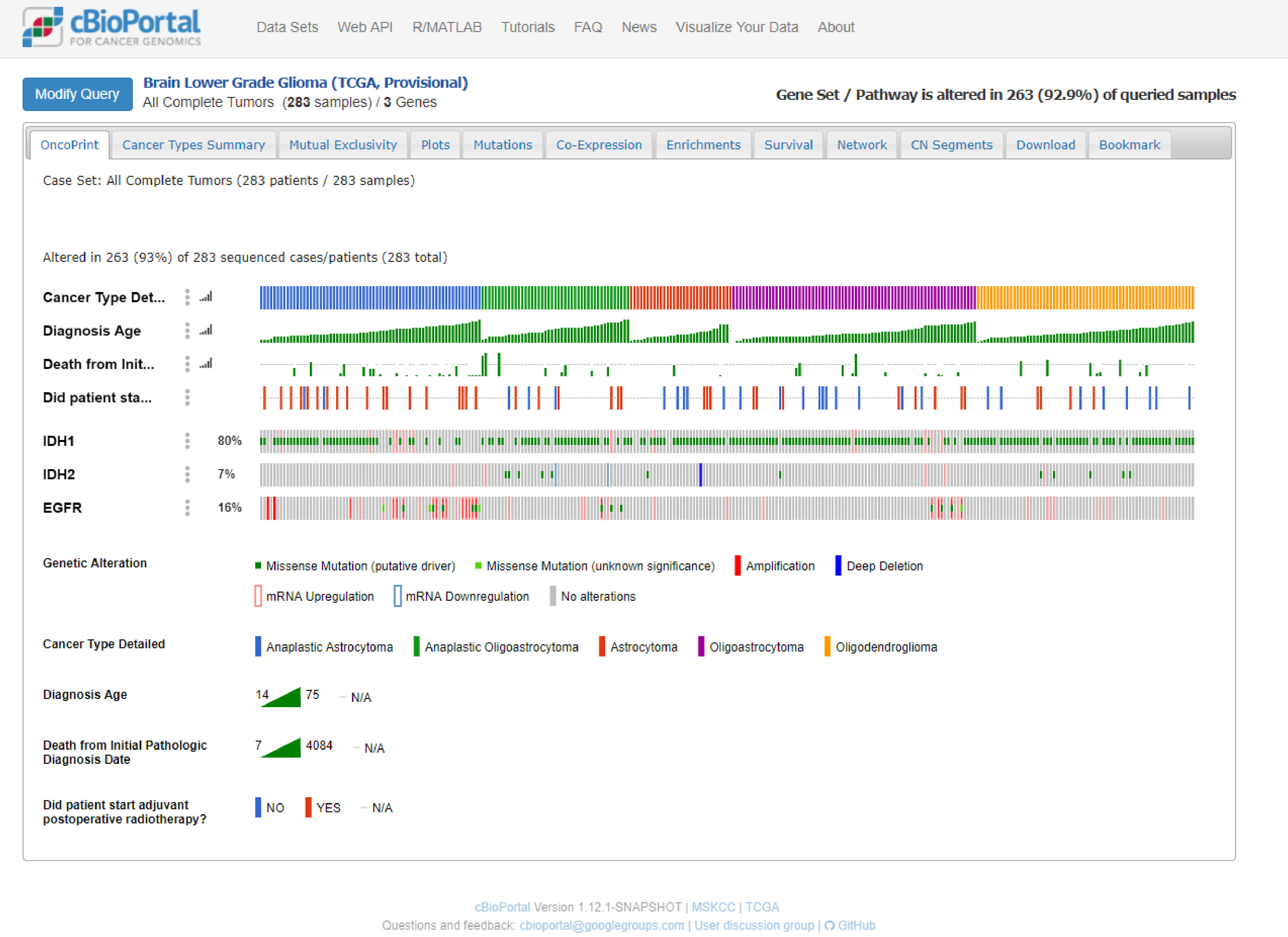
cBioPortal FOR CANCER GENOMICS
Data Sets
Web API
R/MATLAB
Tutorials
FAQ
News
Visualize Your Data
About
Modify Query
Brain Lower Grade Glioma (TCGA, Provisional)
All Complete Tumors (283 samples) / 3 Genes
Gene Set / Pathway is altered in 263 (92.9%) of queried samples
OncoPrint
Cancer Types Summary
Mutual Exclusivity
Plots
Mutations
Co-Expression
Enrichments
Survival
Network
CN Segments
Download
Bookmark
Case Set: All Complete Tumors (283 patients / 283 samples)
Altered in 263 (93%) of 283 sequenced cases/patients (283 total)
Cancer Type Det...
Diagnosis Age
Death from Init...
Did patient sta...
IDH1
80%
IDH2
7%
EGFR
16%
Genetic Alteration
Missense Mutation (putative driver)
Missense Mutation (unknown significance)
Amplification
Deep Deletion
mRNA Upregulation
mRNA Downregulation
No alterations
Cancer Type Detailed
Anaplastic Astrocytoma
Anaplastic Oligoastrocytoma
Astrocytoma
Oligoastrocytoma
Oligodendroglioma
Diagnosis Age
14
75
N/A
Death from Initial Pathologic Diagnosis Date
7
4084
N/A
Did patient start adjuvant postoperative radiotherapy?
NO
YES
N/A
cBioPortal Version 1.12.1-SNAPSHOT | MSKCC | TCGA
Questions and feedback: cbioportal@googlegroups.com | User discussion group | GitHub

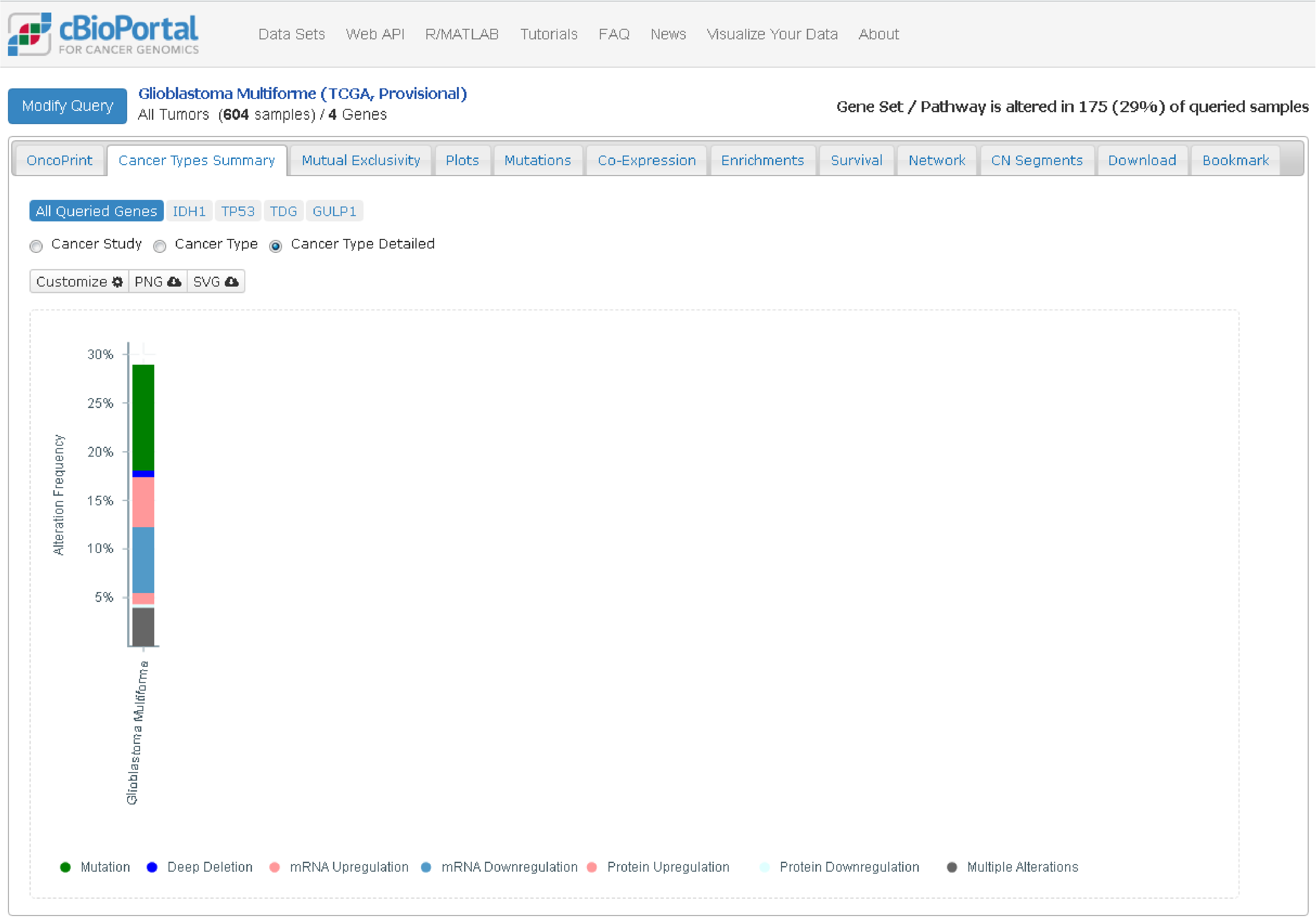
cBioPortal FOR CANCER GENOMICS
Data Sets
Web API
R/MATLAB
Tutorials
FAQ
News
Visualize Your Data
About
Modify Query
Glioblastoma Multiforme (TCGA, Provisional)
All Tumors (604 samples) / 4 Genes
Gene Set / Pathway is altered in 175 (29%) of queried samples
OncoPrint
Cancer Types Summary
Mutual Exclusivity
Plots
Mutations
Co-Expression
Enrichments
Survival
Network
CN Segments
Download
Bookmark
All Queried Genes
IDH1
TP53
TDG
GULP1
Cancer Study
Cancer Type
Cancer Type Detailed
Customize
PNG
SVG
30%
25%
20%
15%
10%
5%
Alteration Frequency
Glioblastoma Multiforme
Mutation
Deep Deletion
mRNA Upregulation
mRNA Downregulation
Protein Upregulation
Protein Downregulation
Multiple Alterations

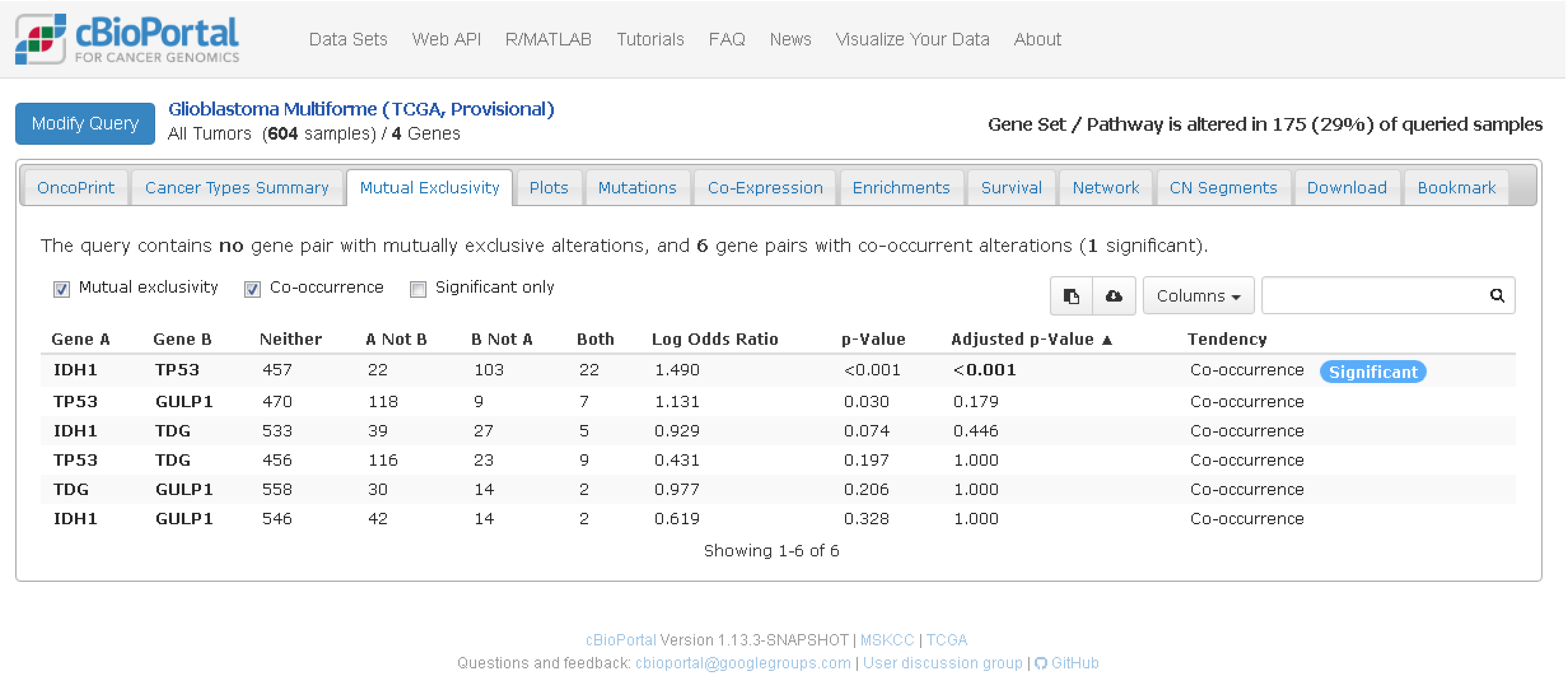

cBioPortal FOR CANCER GENOMICS | Data Sets | Web API | R/MATLAB | Tutorials | FAQ | News | Visualize Your Data | About

Modify Query

Glioblastoma Multiforme (TCGA, Provisional)

All Tumors (**604** samples) / **4** Genes

Gene Set / Pathway is altered in 175 (29%) of queried samples

OncoPrint | Cancer Types Summary | Mutual Exclusivity | Plots | Mutations | Co-Expression | Enrichments | Survival | Network | CN Segments | Download | Bookmark

The query contains **no** gene pair with mutually exclusive alterations, and **6** gene pairs with co-occurrent alterations (**1** significant).

☑ Mutual exclusivity ☑ Co-occurrence ☐ Significant only

Columns

Gene A	Gene B	Neither	A Not B	B Not A	Both	Log Odds Ratio	p-Value	Adjusted p-Value ▲	Tendency
**IDH1**	**TP53**	457	22	103	22	1.490	<0.001	**<0.001**	Co-occurrence Significant
**TP53**	**GULP1**	470	118	9	7	1.131	0.030	0.179	Co-occurrence
**IDH1**	**TDG**	533	39	27	5	0.929	0.074	0.446	Co-occurrence
**TP53**	**TDG**	456	116	23	9	0.431	0.197	1.000	Co-occurrence
**TDG**	**GULP1**	558	30	14	2	0.977	0.206	1.000	Co-occurrence
**IDH1**	**GULP1**	546	42	14	2	0.619	0.328	1.000	Co-occurrence

Showing 1-6 of 6

cBioPortal Version 1.13.3-SNAPSHOT | MSKCC | TCGA

Questions and feedback: cbioportal@googlegroups.com | User discussion group | GitHub

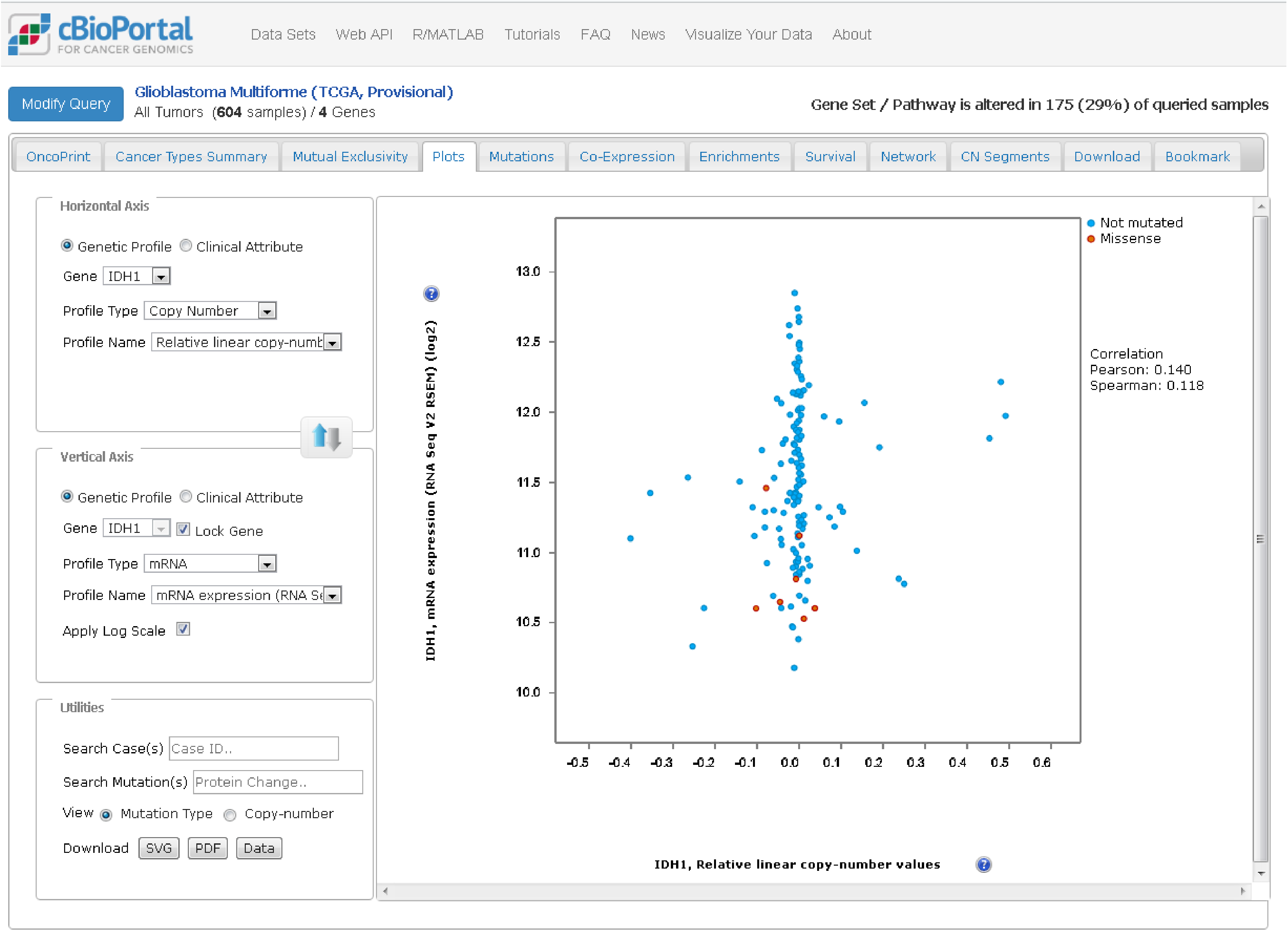
cBioPortal
FOR CANCER GENOMICS
Data Sets
Web API
R/MATLAB
Tutorials
FAQ
News
Visualize Your Data
About
Modify Query
Glioblastoma Multiforme (TCGA, Provisional)
All Tumors (604 samples) / 4 Genes
Gene Set / Pathway is altered in 175 (29%) of queried samples
OncoPrint
Cancer Types Summary
Mutual Exclusivity
Plots
Mutations
Co-Expression
Enrichments
Survival
Network
CN Segments
Download
Bookmark
Horizontal Axis
Genetic Profile
Clinical Attribute
Gene IDH1
Profile Type Copy Number
Profile Name Relative linear copy-numb
Vertical Axis
Genetic Profile
Clinical Attribute
Gene IDH1
Lock Gene
Profile Type mRNA
Profile Name mRNA expression (RNA Se
Apply Log Scale
Utilities
Search Case(s) Case ID..
Search Mutation(s) Protein Change..
View Mutation Type Copy-number
Download SVG PDF Data
Not mutated
Missense
IDH1, mRNA expression (RNA Seq V2 RSEM) (log2)
13.0
12.5
12.0
11.5
11.0
10.5
10.0
-0.5 -0.4 -0.3 -0.2 -0.1 0.0 0.1 0.2 0.3 0.4 0.5 0.6
IDH1, Relative linear copy-number values
Correlation
Pearson: 0.140
Spearman: 0.118

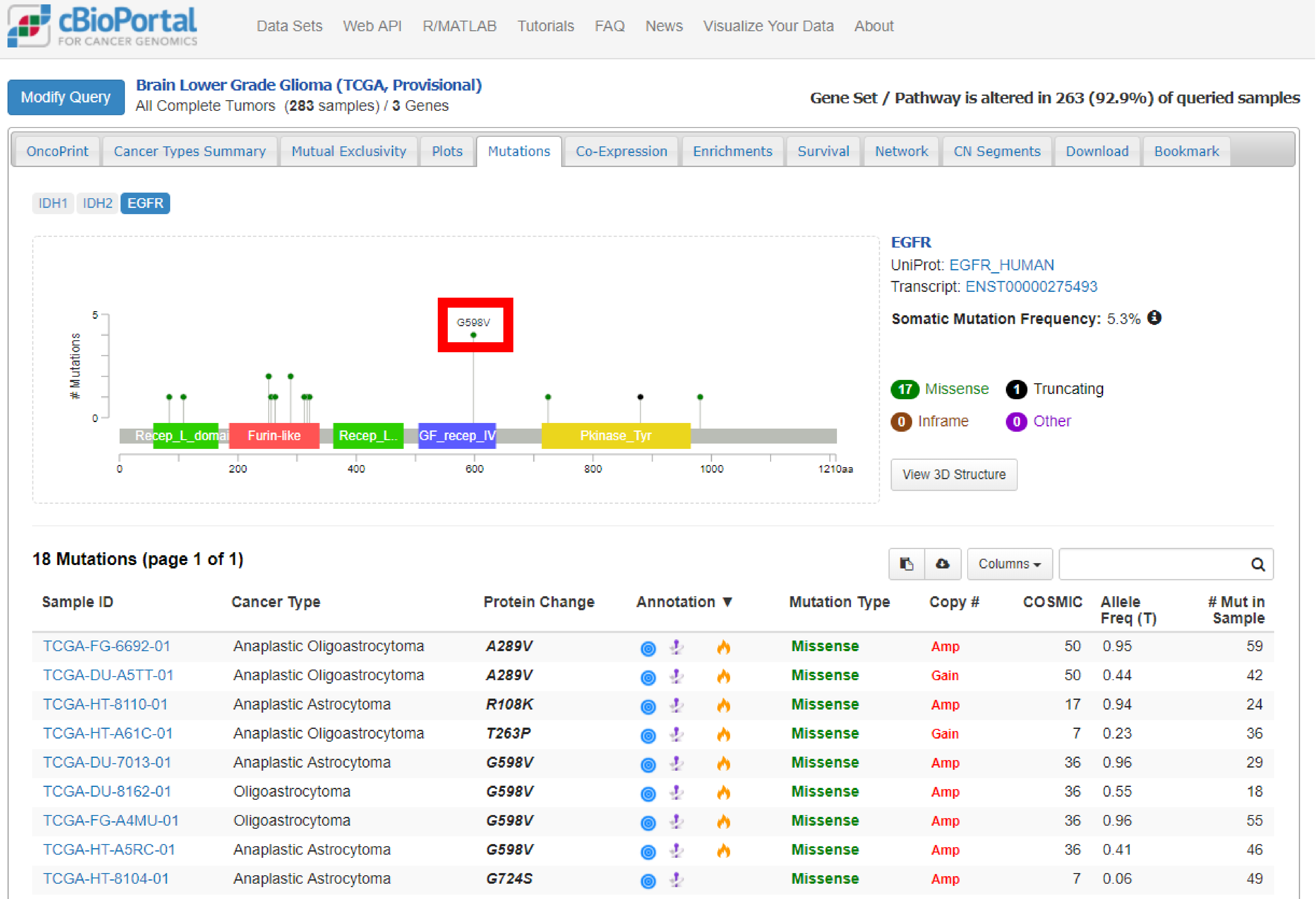

Sample ID	Cancer Type	Protein Change	Annotation ▼	Mutation Type	Copy #	COSMIC	Allele Freq (T)	# Mut in Sample
TCGA-FG-6692-01	Anaplastic Oligoastrocytoma	A289V		Missense	Amp	50	0.95	59
TCGA-DU-A5TT-01	Anaplastic Oligoastrocytoma	A289V		Missense	Gain	50	0.44	42
TCGA-HT-8110-01	Anaplastic Astrocytoma	R108K		Missense	Amp	17	0.94	24
TCGA-HT-A61C-01	Anaplastic Oligoastrocytoma	T263P		Missense	Gain	7	0.23	36
TCGA-DU-7013-01	Anaplastic Astrocytoma	G598V		Missense	Amp	36	0.96	29
TCGA-DU-8162-01	Oligoastrocytoma	G598V		Missense	Amp	36	0.55	18
TCGA-FG-A4MU-01	Oligoastrocytoma	G598V		Missense	Amp	36	0.96	55
TCGA-HT-A5RC-01	Anaplastic Astrocytoma	G598V		Missense	Amp	36	0.41	46
TCGA-HT-8104-01	Anaplastic Astrocytoma	G724S		Missense	Amp	7	0.06	49

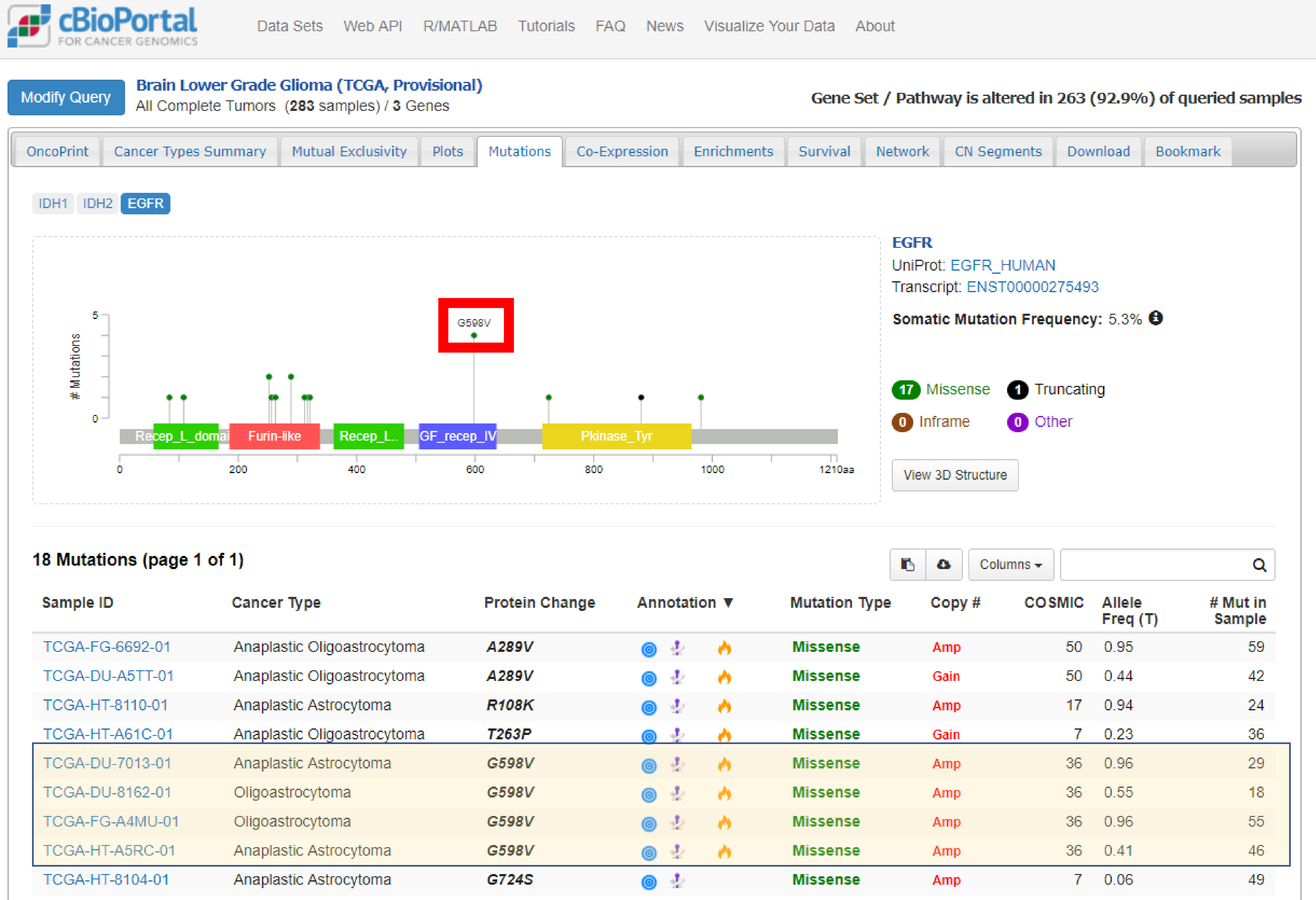

Sample ID	Cancer Type	Protein Change	Annotation	Mutation Type	Copy #	COSMIC	Allele Freq (T)	# Mut in Sample
TCGA-FG-6692-01	Anaplastic Oligoastrocytoma	A289V		Missense	Amp	50	0.95	59
TCGA-DU-A5TT-01	Anaplastic Oligoastrocytoma	A289V		Missense	Gain	50	0.44	42
TCGA-HT-8110-01	Anaplastic Astrocytoma	R108K		Missense	Amp	17	0.94	24
TCGA-HT-A61C-01	Anaplastic Oligoastrocytoma	T263P		Missense	Gain	7	0.23	36
TCGA-DU-7013-01	Anaplastic Astrocytoma	G598V		Missense	Amp	36	0.96	29
TCGA-DU-8162-01	Oligoastrocytoma	G598V		Missense	Amp	36	0.55	18
TCGA-FG-A4MU-01	Oligoastrocytoma	G598V		Missense	Amp	36	0.96	55
TCGA-HT-A5RC-01	Anaplastic Astrocytoma	G598V		Missense	Amp	36	0.41	46
TCGA-HT-8104-01	Anaplastic Astrocytoma	G724S		Missense	Amp	7	0.06	49

cBioPortal FOR CANCER GENOMICS

Data Sets | Web API | R/MATLAB | Tutorials | FAQ | News | Visualize Your Data | About

Modify Query

Glioblastoma Multiforme (TCGA, Provisional)
All Tumors (**604** samples) / **4** Genes

Gene Set / Pathway is altered in 175 (29%) of queried samples

OncoPrint | Cancer Types Summary | Mutual Exclusivity | Plots | Mutations | Co-Expression | Enrichments | Survival | Network | CN Segments | Download | Bookmark

Data Set: mRNA expression (RNA Seq V2 RSEM)
This table lists the genes with the highest expression correlation with the query genes. Click on a row to see the corresponding correlation plot.

IDH1 | TP53 | TDG | GULP1

Search Gene

Show All

Correlated Gene	Cytoband	Pearson's Correlation	Spearman's Correlation
AFF2	Xq28	-0.31	-0.37
GABRA3	Xq28	-0.31	-0.33
MECP2	Xq28	-0.45	-0.47
ZNF275	Xq28	-0.35	-0.39
CD99L2	Xq28	-0.36	-0.38
HSFX2	Xq28	-0.44	-0.45
PNMA3	Xq28	-0.36	-0.41
MAGEC3	Xq27.2	-0.37	-0.42
MCF2	Xq27.1	-0.35	-0.40
LDOC1	Xq27.1	-0.36	-0.40
LINC00632	Xq27.1	-0.35	-0.41
SMIM10L2A	Xq26.3	-0.37	-0.45
SLC9A6	Xq26.3	-0.33	-0.35
HTATSF1	Xq26.3	-0.34	-0.39
TRPC5	Xq23	-0.31	-0.32
AMOT	Xq23	-0.36	-0.39
COL4A5	Xq22.3	-0.44	-0.46
COL4A6	Xq22.3	-0.34	-0.41
DRP2	Xq22.1	-0.35	-0.36
TCEAL2	Xq22.1	-0.34	-0.34
GPRASP2	Xq22.1	-0.34	-0.36
RAB40AL	Xq22.1	-0.31	-0.33
NAP1L3	Xq21.32	-0.33	-0.35
FAM133A	Xq21.32	-0.31	-0.42
SATL1	Xq21.1	-0.34	-0.36
RPS6KA6	Xq21.1	-0.34	-0.39

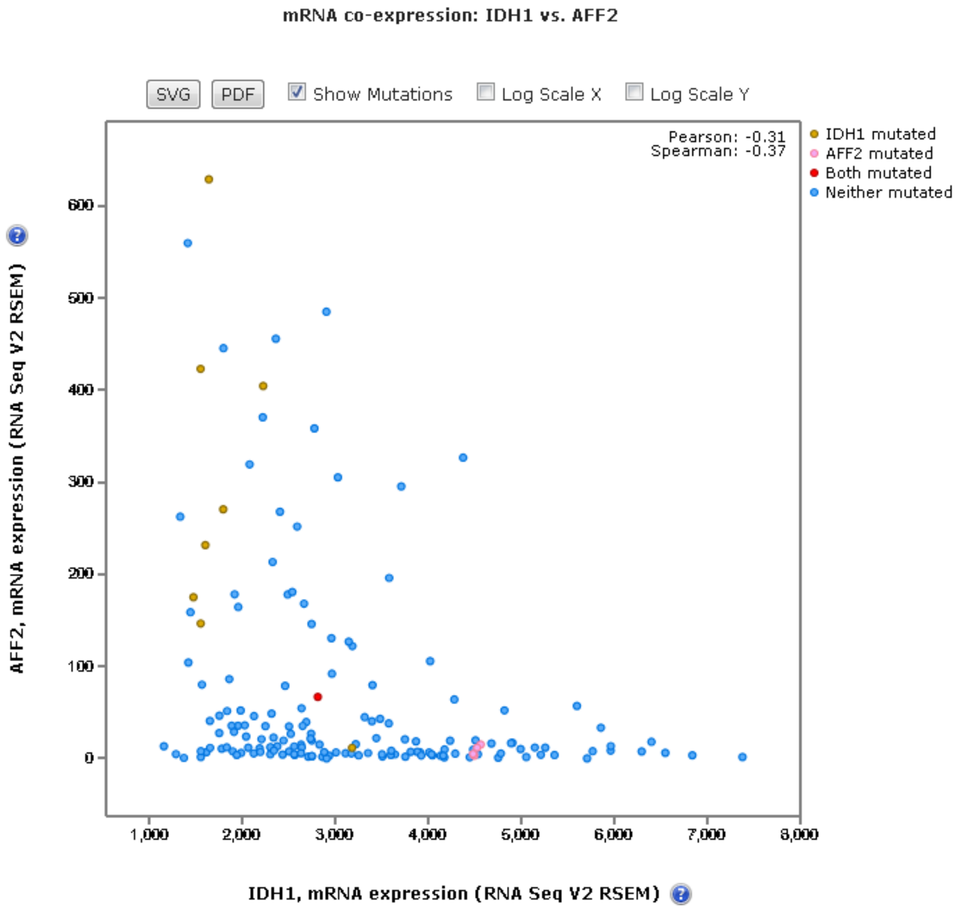

cBioPortal FOR CANCER GENOMICS

Data Sets | Web API | R/MATLAB | Tutorials | FAQ | News | Visualize Your Data | About

Modify Query

Glioblastoma Multiforme (TCGA, Provisional)
All Tumors (**604** samples) / **4** Genes

Gene Set / Pathway is altered in 175 (29%) of queried samples

OncoPrint | Cancer Types Summary | Mutual Exclusivity | Plots | Mutations | Co-Expression | Enrichments | Survival | Network | CN Segments | Download | Bookmark

Mutations | Copy-number | mRNA | Protein

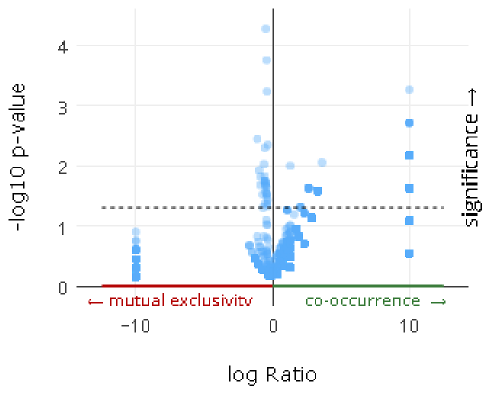

Query Genes
None selected
Select gene in table or volcano plot

Putative copy-number alterations from GISTIC (Deep Deletion)

Add checked genes to query

☑ Mutual exclusivity ☑ Co-occurrence ☐ Significant gene(s)

Search Gene

Gene	Cytoband	Percentage of alteration: In altered group	Percentage of alteration: In unaltered group	Log Ratio	p-Value	q-Value	Direction/Tendency
CDKN2B	9p21.3	72 (43.11%)	251 (61.22%)	-0.51	5.358e-5	0.348	Mutual exclusivity
CDKN2A	9p21.3	76 (45.51%)	255 (62.20%)	-0.45	1.773e-4	0.348	Mutual exclusivity
ATP1B2	17p13.1	6 (3.59%)	0 (0.00%)	>10	5.510e-4	0.348	Co-occurrence
CDKN2A-DT	9p21.3	76 (45.51%)	249 (60.73%)	-0.42	5.900e-4	0.348	Mutual exclusivity
SBF2	11p15.4	5 (2.99%)	0 (0.00%)	>10	1.945e-3	0.348	Co-occurrence
SWAP70	11p15.4	5 (2.99%)	0 (0.00%)	>10	1.945e-3	0.348	Co-occurrence
NRIP3	11p15.4	5 (2.99%)	0 (0.00%)	>10	1.945e-3	0.348	Co-occurrence
RN7SKP50	11p15.4	5 (2.99%)	0 (0.00%)	>10	1.945e-3	0.348	Co-occurrence
TRIM5	11p15.4	5 (2.99%)	0 (0.00%)	>10	1.945e-3	0.348	Co-occurrence
WRAP53	17p13.1	5 (2.99%)	0 (0.00%)	>10	1.945e-3	0.348	Co-occurrence
MIR491	9p21.3	12 (7.19%)	64 (15.61%)	-1.12	3.621e-3	0.348	Mutual exclusivity
MTAP	9p21.3	73 (43.71%)	230 (56.10%)	-0.36	4.526e-3	0.348	Mutual exclusivity
DMRTA1	9p21.3	38 (22.75%)	139 (33.90%)	-0.58	5.083e-3	0.348	Mutual exclusivity
ACCSL	11p11.2	4 (2.40%)	0 (0.00%)	>10	6.839e-3	0.348	Co-occurrence

Showing 1 to 14 of 12,405 entries

2 3 4 5 ... 887 Next Last

Download Complete Result

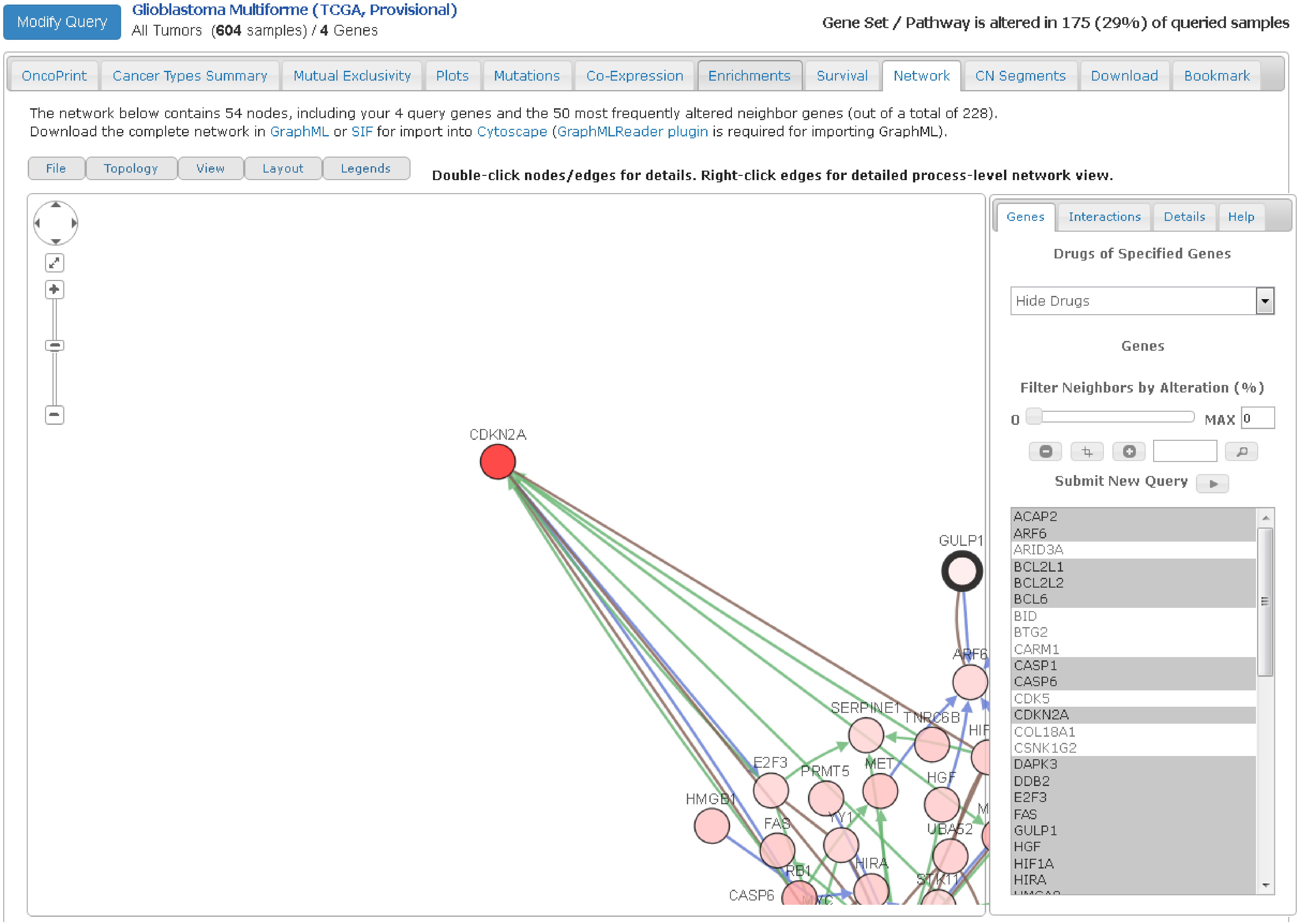
Modify Query
Glioblastoma Multiforme (TCGA, Provisional)
All Tumors (604 samples) / 4 Genes
Gene Set / Pathway is altered in 175 (29%) of queried samples
OncoPrint
Cancer Types Summary
Mutual Exclusivity
Plots
Mutations
Co-Expression
Enrichments
Survival
Network
CN Segments
Download
Bookmark
The network below contains 54 nodes, including your 4 query genes and the 50 most frequently altered neighbor genes (out of a total of 228).
Download the complete network in GraphML or SIF for import into Cytoscape (GraphMLReader plugin is required for importing GraphML).
File
Topology
View
Layout
Legends
Double-click nodes/edges for details. Right-click edges for detailed process-level network view.
CDKN2A
GULP1
ARF6
SERPINE1
TNRC6B
HIF
E2F3
PRMT5
MET
HGF
HMGB1
FAS
YY1
UBA52
RB1
HIRA
STK11
CASP6
Genes
Interactions
Details
Help
Drugs of Specified Genes
Hide Drugs
Genes
Filter Neighbors by Alteration (%)
0
MAX
Submit New Query
ACAP2
ARF6
ARID3A
BCL2L1
BCL2L2
BCL6
BID
BTG2
CARM1
CASP1
CASP6
CDK5
CDKN2A
COL18A1
CSNK1G2
DAPK3
DDB2
E2F3
FAS
GULP1
HGF
HIF1A
HIRA

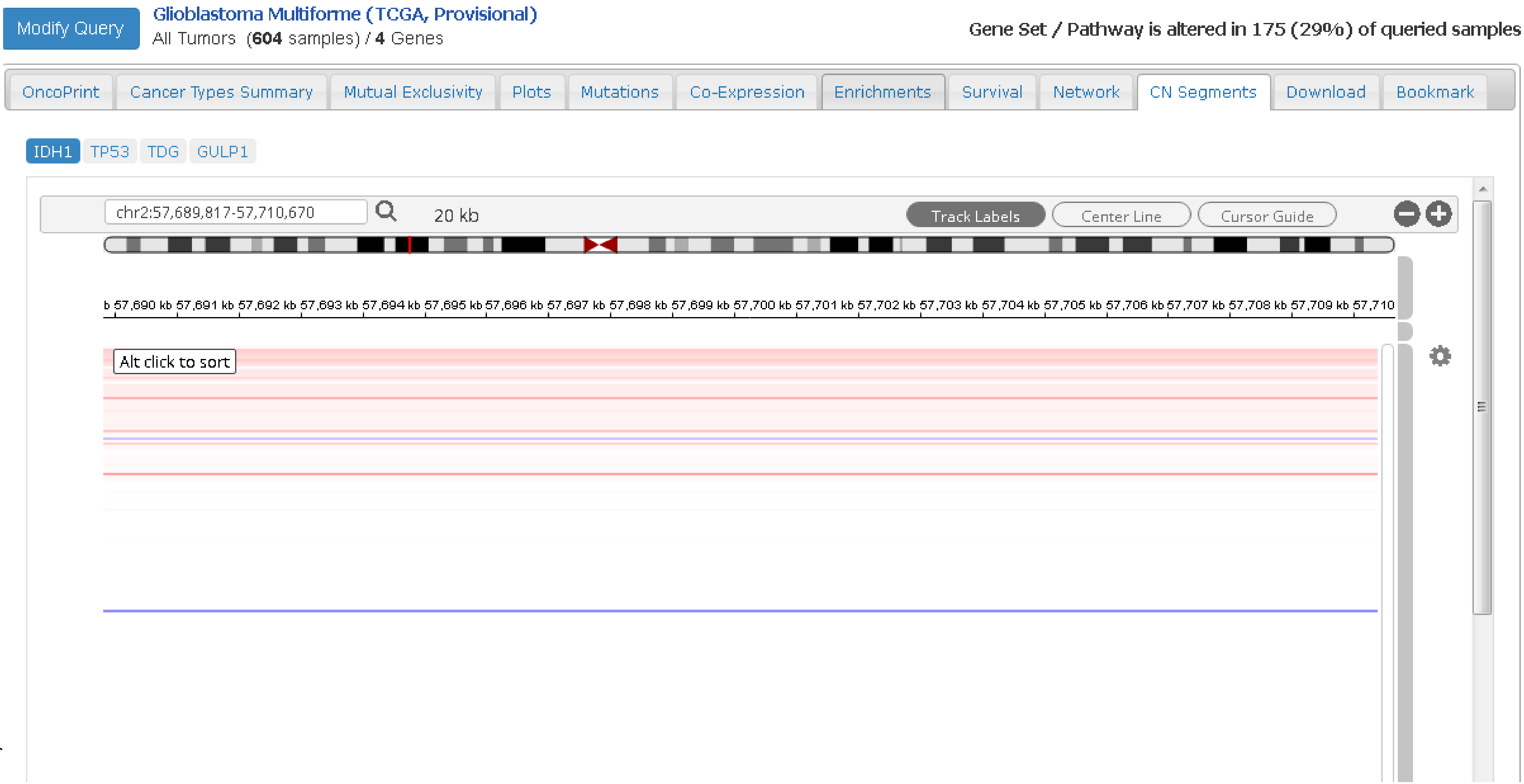
Modify Query
Glioblastoma Multiforme (TCGA, Provisional)
All Tumors (604 samples) / 4 Genes
Gene Set / Pathway is altered in 175 (29%) of queried samples
OncoPrint
Cancer Types Summary
Mutual Exclusivity
Plots
Mutations
Co-Expression
Enrichments
Survival
Network
CN Segments
Download
Bookmark
IDH1
TP53
TDG
GULP1
chr2:57,689,817-57,710,670
20 kb
Track Labels
Center Line
Cursor Guide
Alt click to sort
