# Supplementary material for: Requirements Analysis and Specification for a Molecular Tumor Board Platform Based on cBioPortal
Source: Diagnostics (Basel). 2020 Feb 10;10(2):93. doi: 10.3390/diagnostics10020093 (PMC7167859; doi:10.3390/diagnostics10020093)
Supplement: Supplementary file 1 [file diagnostics-10-00093-s001.zip › Supplementary File 4_Presentation of potential tools.pdf]

Requirements Analysis and Specification for a Molecular Tumor Board Platform Based on cBioPortal (Buechner P. et al.)
Supplementary File 4: Presentation of potential tools

Note

Since we conducted the interviews in German language, these slides were translated for this publication.

SOPHiA GENETICS

1. SOPHiA GENETICS

Online platform for analysis of high-throughput sequencing data and clinical interpretation of variants. It is also possible to share the evaluation of variants with other institutions.

SNV/INDELs

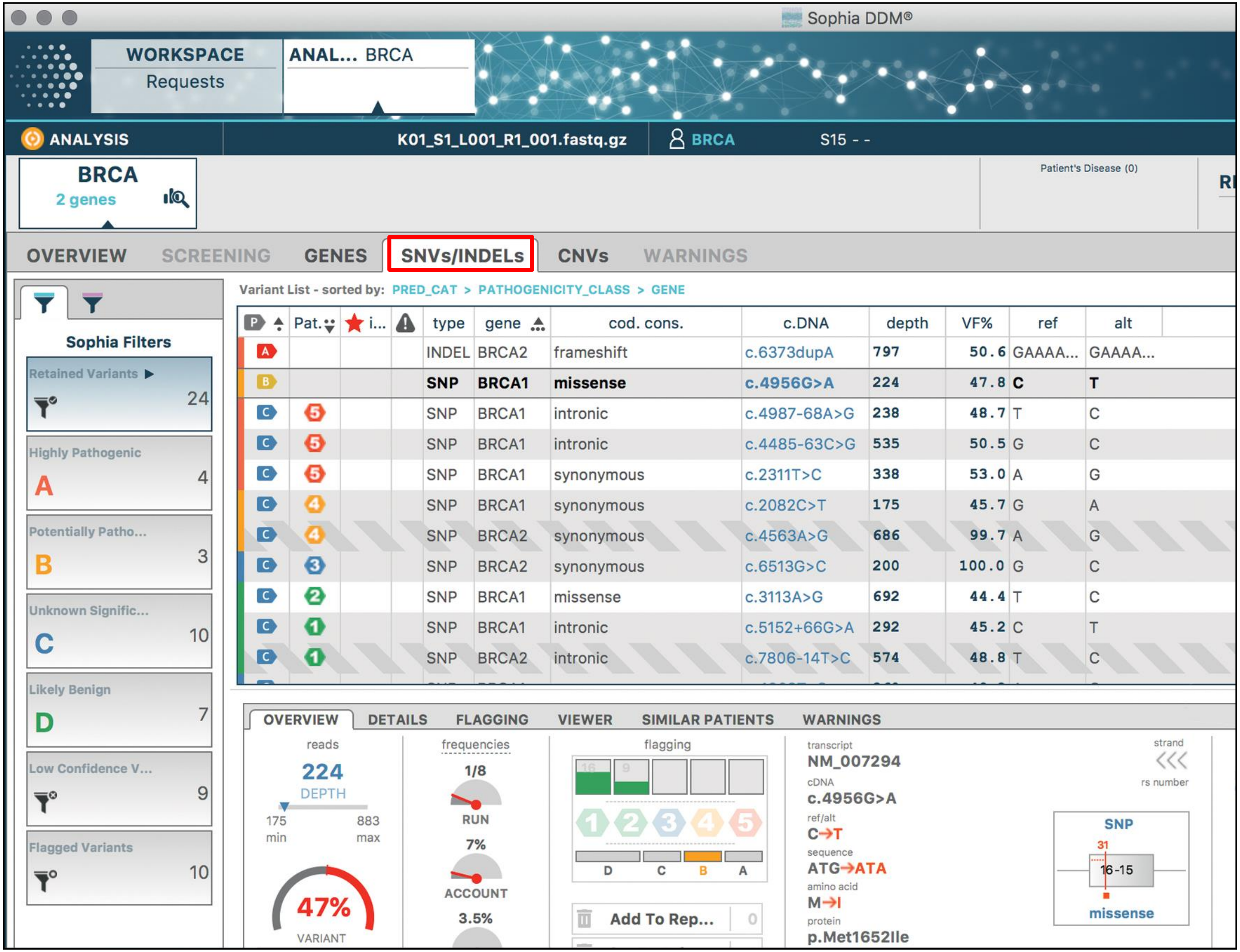
Sophia DDM®
WORKSPACE
Requests
ANAL... BRCA
ANALYSIS
K01_S1_L001_R1_001.fastq.gz
BRCA
S15 - -
BRCA
2 genes
Patient's Disease (0)
OVERVIEW
SCREENING
GENES
SNVs/INDELs
CNVs
WARNINGS
Sophia Filters
Retained Variants 24
Highly Pathogenic A 4
Potentially Patho... B 3
Unknown Signific... C 10
Likely Benign D 7
Low Confidence V... 9
Flagged Variants 10
Variant List - sorted by: PRED_CAT > PATHOGENICITY_CLASS > GENE
Pat. i... type gene cod. cons. c.DNA depth VF% ref alt
INDEL BRCA2 frameshift c.6373dupA 797 50.6 GAAAA... GAAAA...
SNP BRCA1 missense c.4956G>A 224 47.8 C T
SNP BRCA1 intronic c.4987-68A>G 238 48.7 T C
SNP BRCA1 intronic c.4485-63C>G 535 50.5 G C
SNP BRCA1 synonymous c.2311T>C 338 53.0 A G
SNP BRCA1 synonymous c.2082C>T 175 45.7 G A
SNP BRCA2 synonymous c.4563A>G 686 99.7 A G
SNP BRCA2 synonymous c.6513G>C 200 100.0 G C
SNP BRCA1 missense c.3113A>G 692 44.4 T C
SNP BRCA1 intronic c.5152+66G>A 292 45.2 C T
SNP BRCA2 intronic c.7806-14T>C 574 48.8 T C
OVERVIEW
DETAILS
FLAGGING
VIEWER
SIMILAR PATIENTS
WARNINGS
reads
224
DEPTH
175 min
883 max
47%
VARIANT
frequencies
1/8
RUN
7%
ACCOUNT
3.5%
flagging
D C B A
Add To Rep...
transcript
NM_007294
cDNA
c.4956G>A
ref/alt
C→T
sequence
ATG→ATA
amino acid
M→I
protein
p.Met1652Ile
strand
rs number
SNP
16-15
missense

CNVs

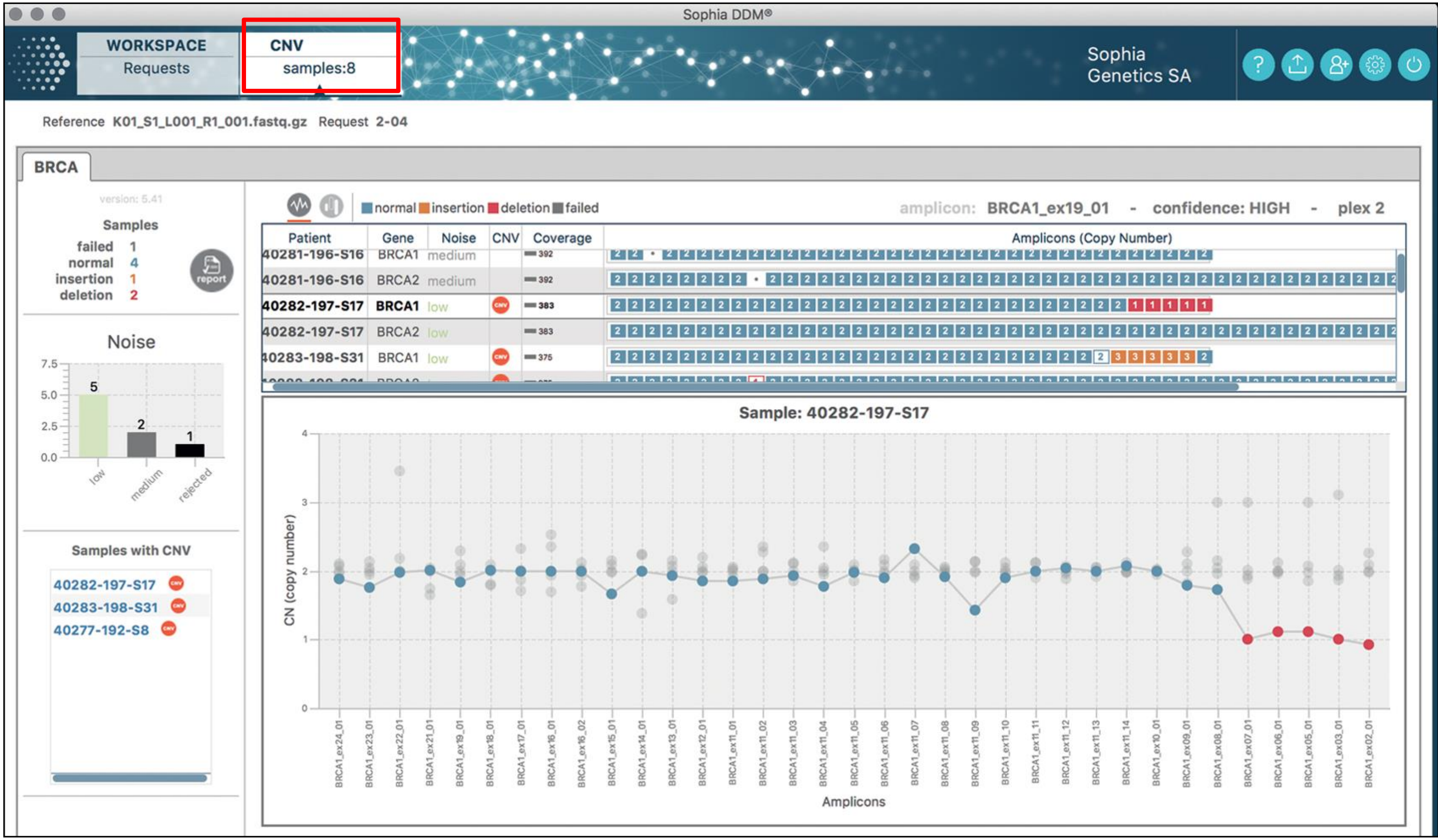
Sophia DDM®
WORKSPACE
Requests
CNV
samples:8
Sophia Genetics SA
Reference K01_S1_L001_R1_001.fastq.gz Request 2-04
BRCA
Samples
failed 1
normal 4
insertion 1
deletion 2
report
Noise
low
medium
rejected
Samples with CNV
40282-197-S17
40283-198-S31
40277-192-S8
normal insertion deletion failed
amplicon: BRCA1_ex19_01 - confidence: HIGH - plex 2
Patient
Gene
Noise
CNV
Coverage
Amplicons (Copy Number)
40281-196-S16 BRCA1 medium
40281-196-S16 BRCA2 medium
40282-197-S17 BRCA1 low
40282-197-S17 BRCA2 low
40283-198-S31 BRCA1 low
Sample: 40282-197-S17
CN (copy number)
Amplicons

Variants Report

SOPHiA GENETICS

Variant Report

Patient

First name:
Last name:
Patient ID: **SG10000002**
DOB: **2 Jan 1967**
Gender: **Female**
Father ethnicity: **-- Unknown --**
Mother ethnicity: **-- Unknown --**

Analysis by

SOPHiA™
The AI for Data-Driven Medicine

Analysis

Analysis ID: **40538**
MID: **S2**
Request run date:
Request run name: **HCS by Sophia**

RESULTS

SNVs/INDELs (selected in report)

Gene Transcript	Exon	c.DNA Protein alteration	Variant Fraction Coverage (ref / alt)	Coding consequence	Pathogenicity	ClinVar
BRCA1 NM_007294	10	c.4065.4068delTCAA p.Asn1355Lysfs*10	47.3 % (785 / 709)	frameshift	Prediction A I Highly Pathogenic	rs80357508
comment: The detected variant is highly pathogenic						

SNVs/INDELs (retained)

Gene Transcript	Exon	c.DNA Protein alteration	Variant Fraction Coverage (ref / alt)	Coding consequence	Pathogenicity	ClinVar
ATM NM_000051	3	c.146C>G p.Ser49Cys	51.7 % (1009 / 1081)	missense	Prediction B I Potentially Pathogenic	rs1800054
ATM NM_000051	40	c.5948A>G p.Asn1983Ser	100.0 % (0 / 1750)	missense	Prediction C I Unknown Significance	rs659243
BARD1 NM_000465	1	c.70C>T p.Pro24Ser	48.75 % (818 / 778)	missense	Prediction C I Unknown Significance	rs1048108
BARD1 NM_000465	6	c.1518T>C p.= (p.His506His)	100.0 % (0 / 1753)	synonymous	Prediction C I Unknown Significance	rs2070093

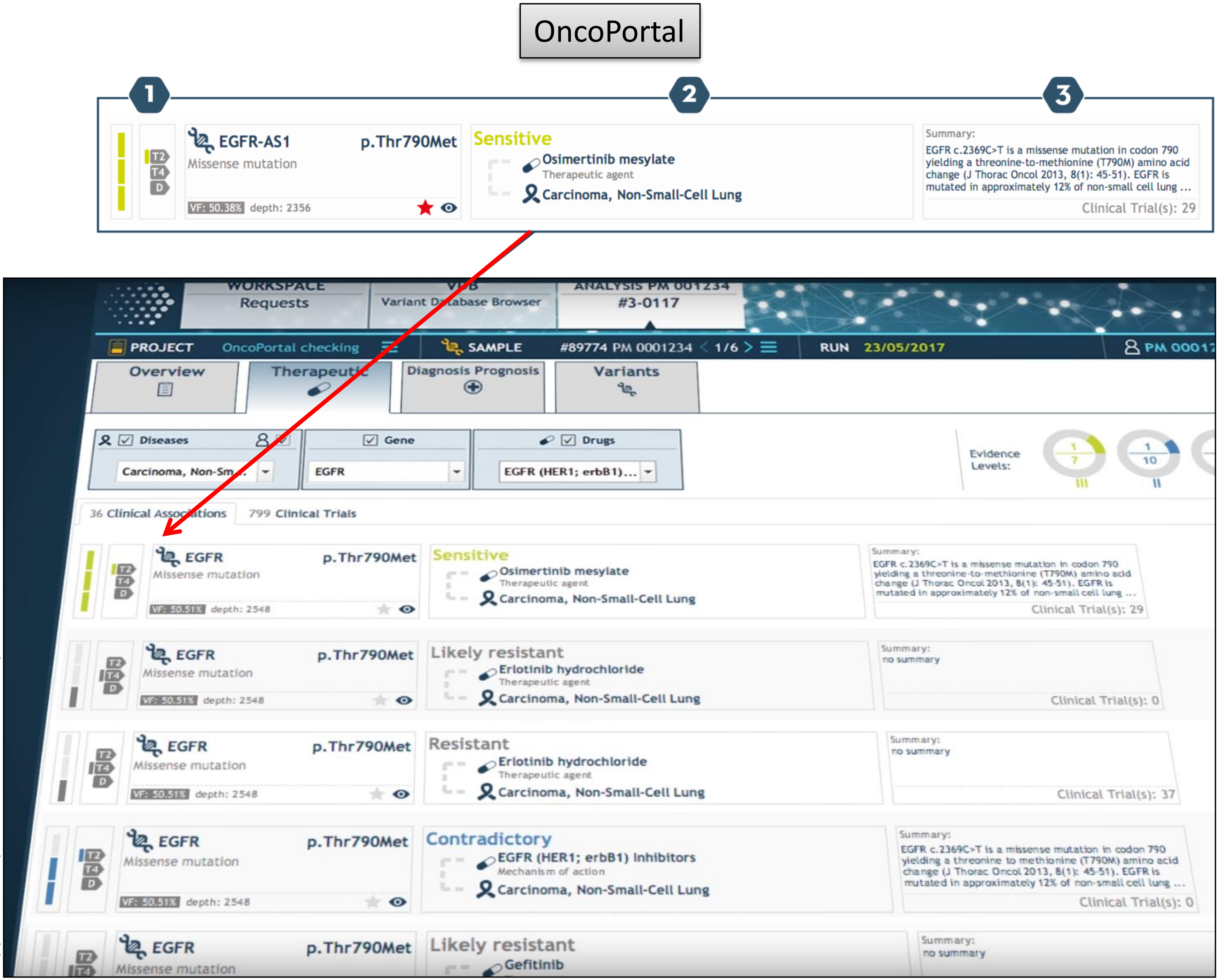
OncoPortal
1
2
3
EGFR-AS1
p.Thr790Met
Missense mutation
VF: 50.38%
depth: 2356
Sensitive
Osimertinib mesylate
Therapeutic agent
Carcinoma, Non-Small-Cell Lung
Summary:
EGFR c.2369C>T is a missense mutation in codon 790 yielding a threonine-to-methionine (T790M) amino acid change (J Thorac Oncol 2013, 8(1): 45-51). EGFR is mutated in approximately 12% of non-small cell lung ...
Clinical Trial(s): 29
Requests
Variant Database Browser
#3-0117
PROJECT
OncoPortal checking
SAMPLE
#89774 PM 0001234
1/6
RUN
23/05/2017
Overview
Therapeutic
Diagnosis Prognosis
Variants
Diseases
Carcinoma, Non-Sm...
Gene
EGFR
Drugs
EGFR (HER1; erbB1)...
Evidence Levels:
36 Clinical Associations
799 Clinical Trials
EGFR
p.Thr790Met
Missense mutation
VF: 50.51%
depth: 2548
Sensitive
Osimertinib mesylate
Therapeutic agent
Carcinoma, Non-Small-Cell Lung
Clinical Trial(s): 29
Likely resistant
Erlotinib hydrochloride
Therapeutic agent
Carcinoma, Non-Small-Cell Lung
Summary:
no summary
Clinical Trial(s): 0
Resistant
Erlotinib hydrochloride
Clinical Trial(s): 37
Contradictory
EGFR (HER1; erbB1) Inhibitors
Mechanism of action
Clinical Trial(s): 0
Likely resistant
Gefitinib

2. CIViC

Cites from CIViC homepage & nature paper:

- *"CIViC is an* ***open access, open source****, community-driven web resource for Clinical Interpretation of Variants in Cancer."*
- *"CIViC is an* ***expert-crowdsourced knowledgebase*** *for Clinical Interpretation of Variants in Cancer describing the therapeutic, prognostic, diagnostic and predisposing relevance of inherited and somatic variants of all types"*

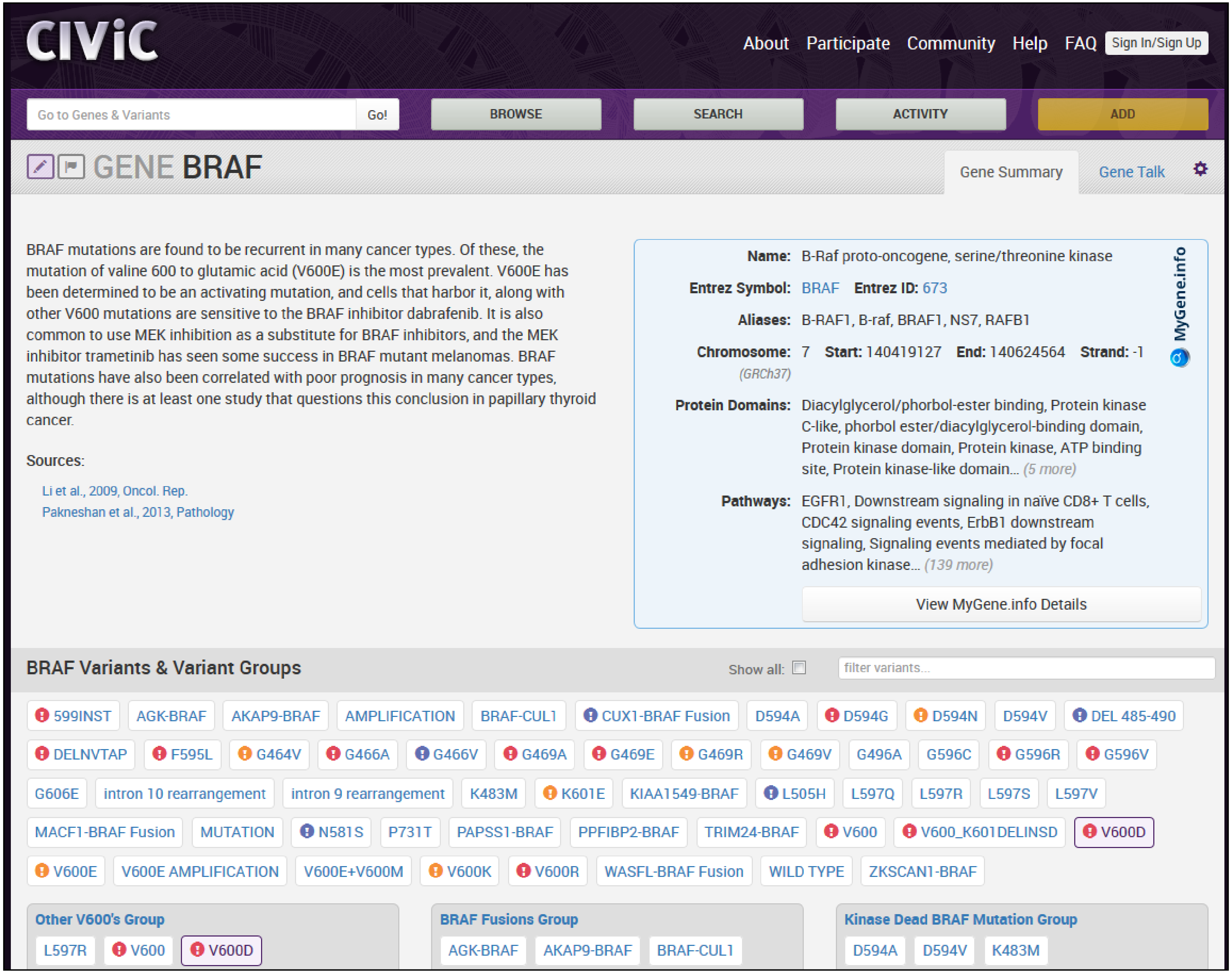
CIViC
About
Participate
Community
Help
FAQ
Sign In/Sign Up
Go to Genes & Variants
Go!
BROWSE
SEARCH
ACTIVITY
ADD
GENE BRAF
Gene Summary
Gene Talk
BRAF mutations are found to be recurrent in many cancer types. Of these, the mutation of valine 600 to glutamic acid (V600E) is the most prevalent. V600E has been determined to be an activating mutation, and cells that harbor it, along with other V600 mutations are sensitive to the BRAF inhibitor dabrafenib. It is also common to use MEK inhibition as a substitute for BRAF inhibitors, and the MEK inhibitor trametinib has seen some success in BRAF mutant melanomas. BRAF mutations have also been correlated with poor prognosis in many cancer types, although there is at least one study that questions this conclusion in papillary thyroid cancer.
Sources:
Li et al., 2009, Oncol. Rep.
Pakneshan et al., 2013, Pathology
Name: B-Raf proto-oncogene, serine/threonine kinase
Entrez Symbol: BRAF Entrez ID: 673
Aliases: B-RAF1, B-raf, BRAF1, NS7, RAFB1
Chromosome: 7 Start: 140419127 End: 140624564 Strand: -1
(GRCh37)
Protein Domains: Diacylglycerol/phorbol-ester binding, Protein kinase C-like, phorbol ester/diacylglycerol-binding domain, Protein kinase domain, Protein kinase, ATP binding site, Protein kinase-like domain... (5 more)
Pathways: EGFR1, Downstream signaling in naïve CD8+ T cells, CDC42 signaling events, ErbB1 downstream signaling, Signaling events mediated by focal adhesion kinase... (139 more)
View MyGene.info Details
MyGene.info
BRAF Variants & Variant Groups
Show all:
filter variants...
599INST AGK-BRAF AKAP9-BRAF AMPLIFICATION BRAF-CUL1 CUX1-BRAF Fusion D594A D594G D594N D594V DEL 485-490
DELNVTAP F595L G464V G466A G466V G469A G469E G469R G469V G496A G596C G596R G596V
G606E intron 10 rearrangement intron 9 rearrangement K483M K601E KIAA1549-BRAF L505H L597Q L597R L597S L597V
MACF1-BRAF Fusion MUTATION N581S P731T PAPSS1-BRAF PPFIBP2-BRAF TRIM24-BRAF V600 V600_K601DELINSD V600D
V600E V600E AMPLIFICATION V600E+V600M V600K V600R WASFL-BRAF Fusion WILD TYPE ZKSCAN1-BRAF
Other V600's Group
L597R V600 V600D
BRAF Fusions Group
AGK-BRAF AKAP9-BRAF BRAF-CUL1
Kinase Dead BRAF Mutation Group
D594A D594V K483M

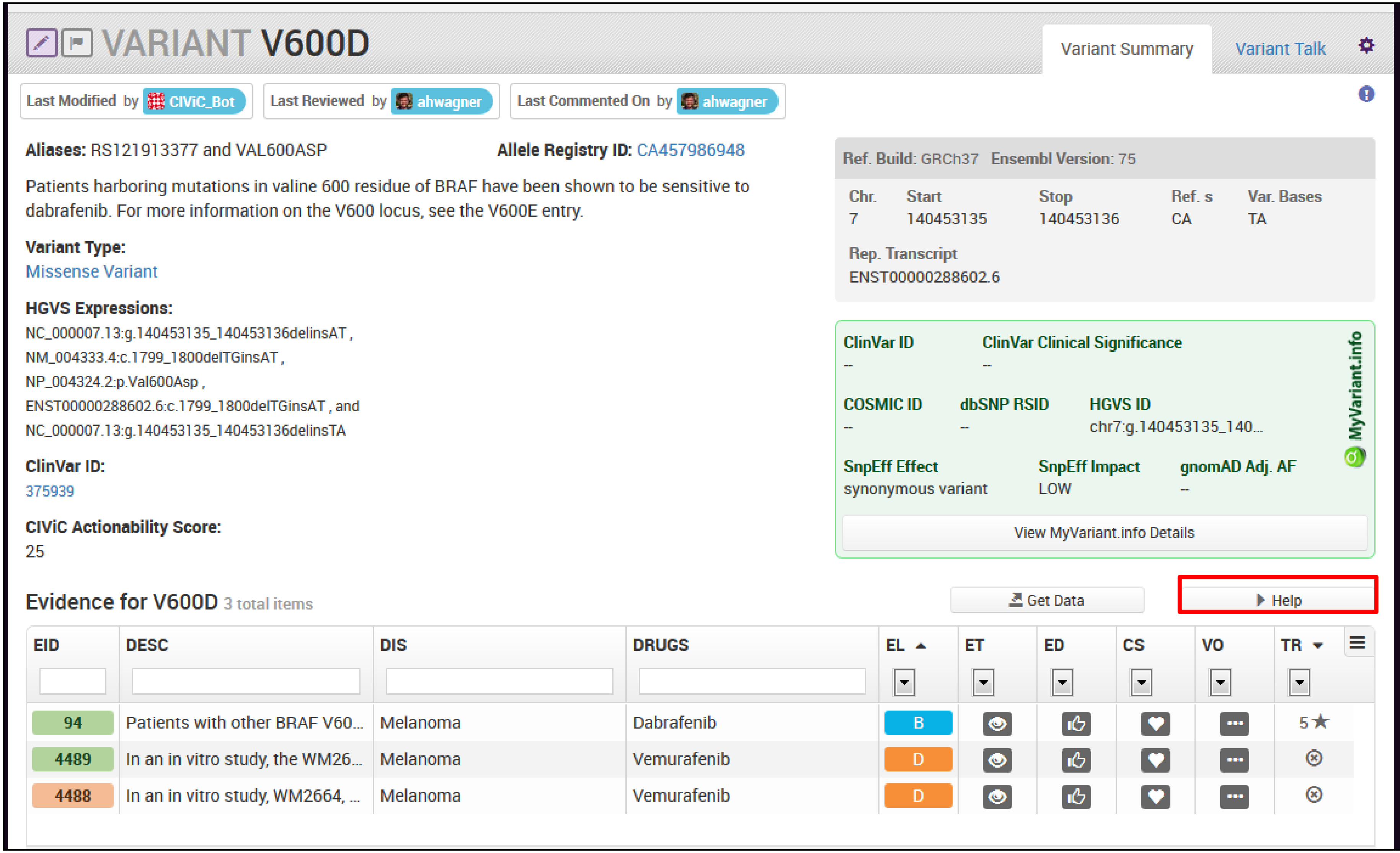
VARIANT V600D
Variant Summary
Variant Talk
Last Modified by CIViC_Bot
Last Reviewed by ahwagner
Last Commented On by ahwagner
Aliases: RS121913377 and VAL600ASP
Allele Registry ID: CA457986948
Patients harboring mutations in valine 600 residue of BRAF have been shown to be sensitive to dabrafenib. For more information on the V600 locus, see the V600E entry.
Variant Type:
Missense Variant
HGVS Expressions:
NC_000007.13:g.140453135_140453136delinsAT ,
NM_004333.4:c.1799_1800delTGinsAT ,
NP_004324.2:p.Val600Asp ,
ENST00000288602.6:c.1799_1800delTGinsAT , and
NC_000007.13:g.140453135_140453136delinsTA
ClinVar ID:
375939
CIViC Actionability Score:
25
Ref. Build: GRCh37 Ensembl Version: 75
Chr. 7
Start 140453135
Stop 140453136
Ref. s CA
Var. Bases TA
Rep. Transcript ENST00000288602.6
ClinVar ID --
ClinVar Clinical Significance --
COSMIC ID --
dbSNP RSID --
HGVS ID chr7:g.140453135_140...
SnpEff Effect synonymous variant
SnpEff Impact LOW
gnomAD Adj. AF --
MyVariant.info
View MyVariant.info Details
Evidence for V600D 3 total items
Get Data
Help
EID DESC DIS DRUGS EL ET ED CS VO TR
94 Patients with other BRAF V60... Melanoma Dabrafenib B 5
4489 In an in vitro study, the WM26... Melanoma Vemurafenib D
4488 In an in vitro study, WM2664, ... Melanoma Vemurafenib D

Column Abbreviation	Column Description
**EID**	**Evidence ID Number**
	Submitted
	Accepted
	Rejected
	Has pending revisions
**DESC**	**Evidence Description**
**DIS**	**Disease**
**DRUG**	**Drug(s)**
**EL**	**Evidence Level**
	A Validated
	B Clinical
	C Case Study
	D Preclinical
	E Inferential
**ET**	**Evidence Type**
	Predictive
	Diagnostic
	Prognostic
	Predisposing

Column Abbreviation	Column Description
**ED**	**Evidence Direction**
	Supports
	Does Not Support
**CS**	**Clinical Significance**
	Sensitivity/Response
	Resistance
	Better Outcome
	Poor Outcome
	Positive
	Negative
	Adverse Response
	Reduced Sensitivity
	Pathogenic
	Likely Pathogenic
	Benign
	Likely Benign
	Uncertain Significance
	N/A
**VO**	**Variant Origin**
	Germline Mutation
	Somatic Mutation
	Germline Polymorphism
	Unknown

Clarivate Analytics

3. Clarivate MetaCore® / Key Pathway Advisor®

commercial product
just for comparison with industrial products
no replacement for cBioPortal

Übersicht MetaCore

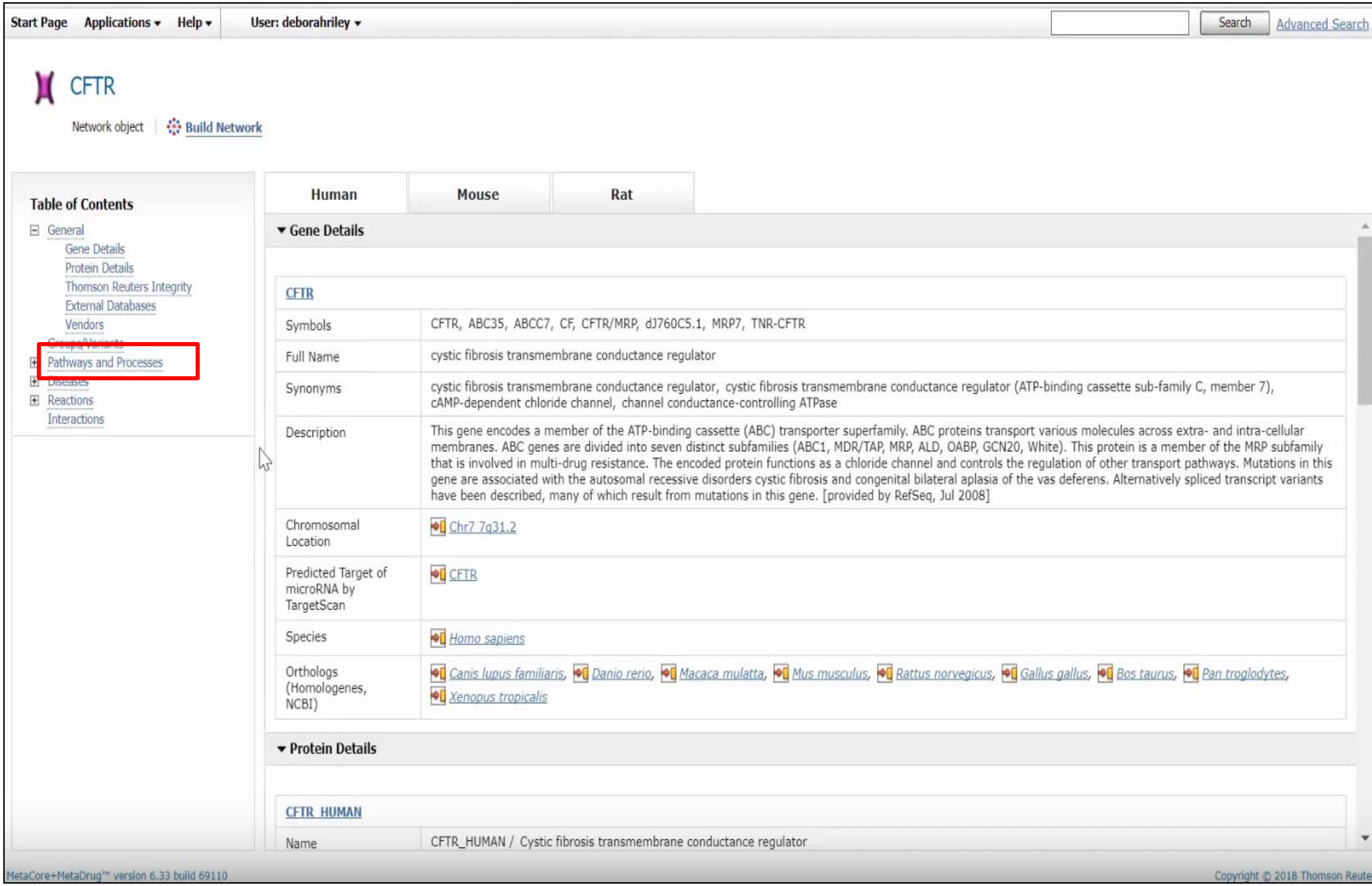

MetaCore: Map 1

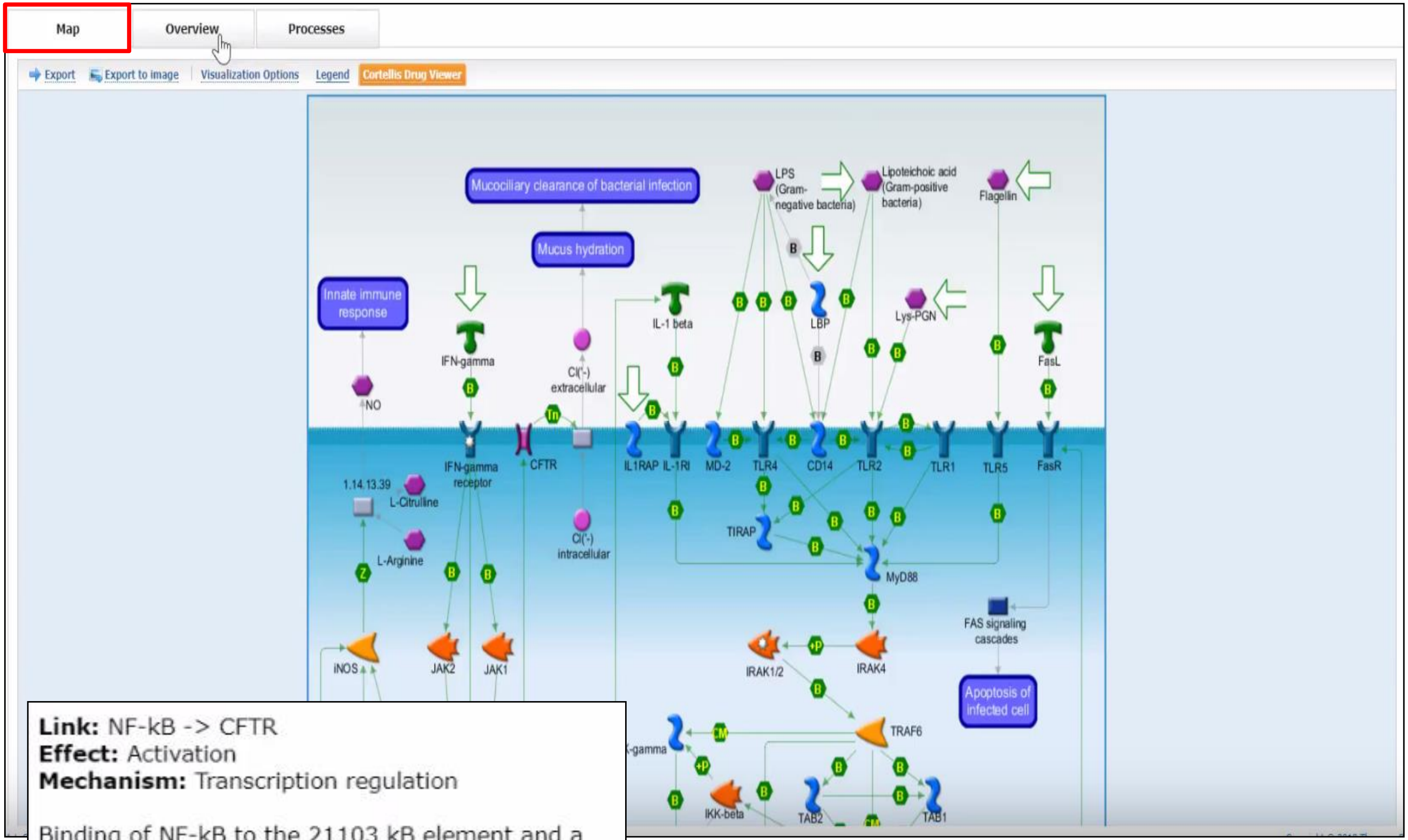

Link: NF-kB -> CFTR
Effect: Activation
Mechanism: Transcription regulation

Binding of NF-kB to the 21103 kB element and a subsequent increase in CFTR promoter activity leads to CFTR mRNA increase in response to IL-1 beta in Calu-3 cells.

MetaCore: Map 2

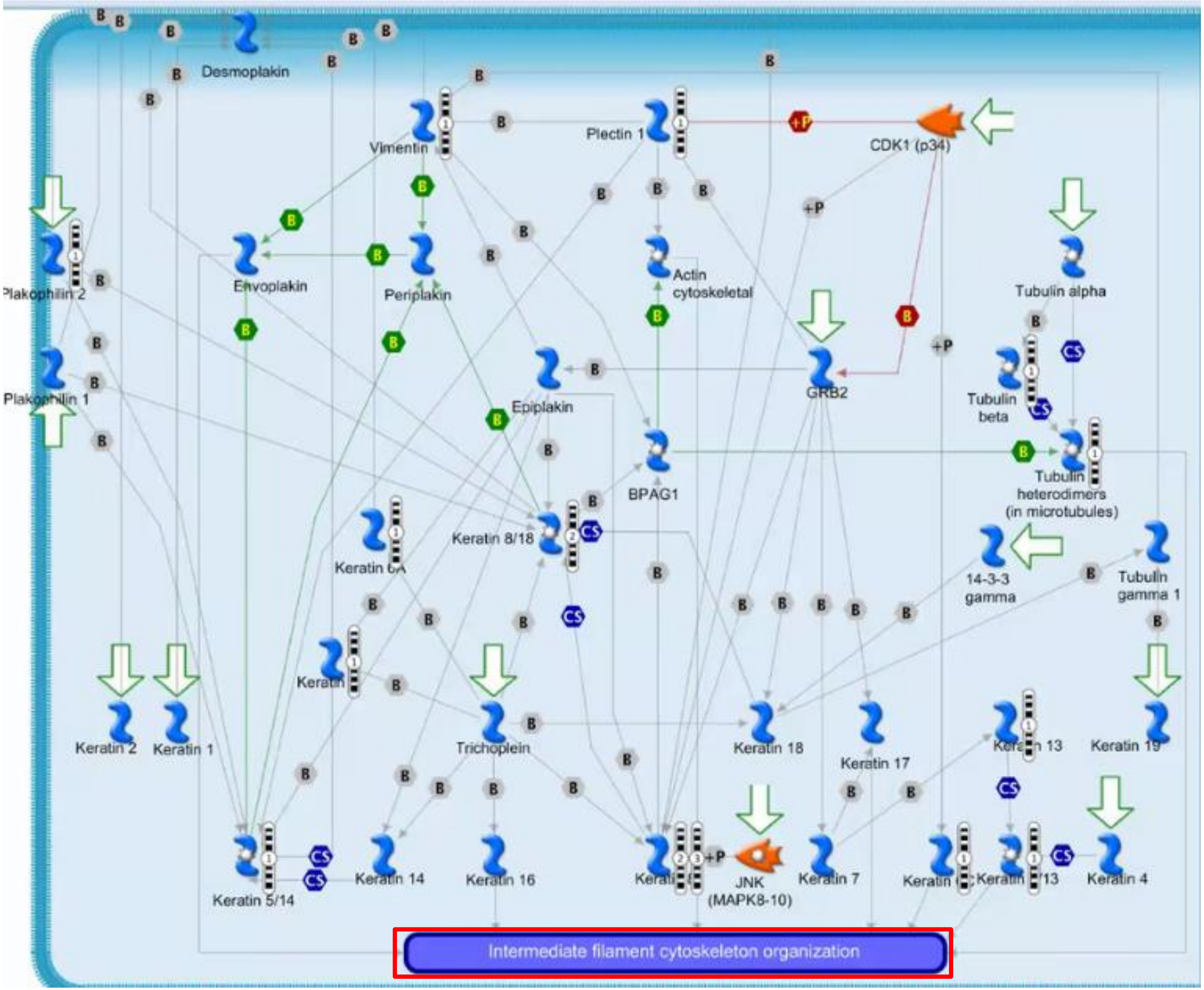

MetaCore: Map 3

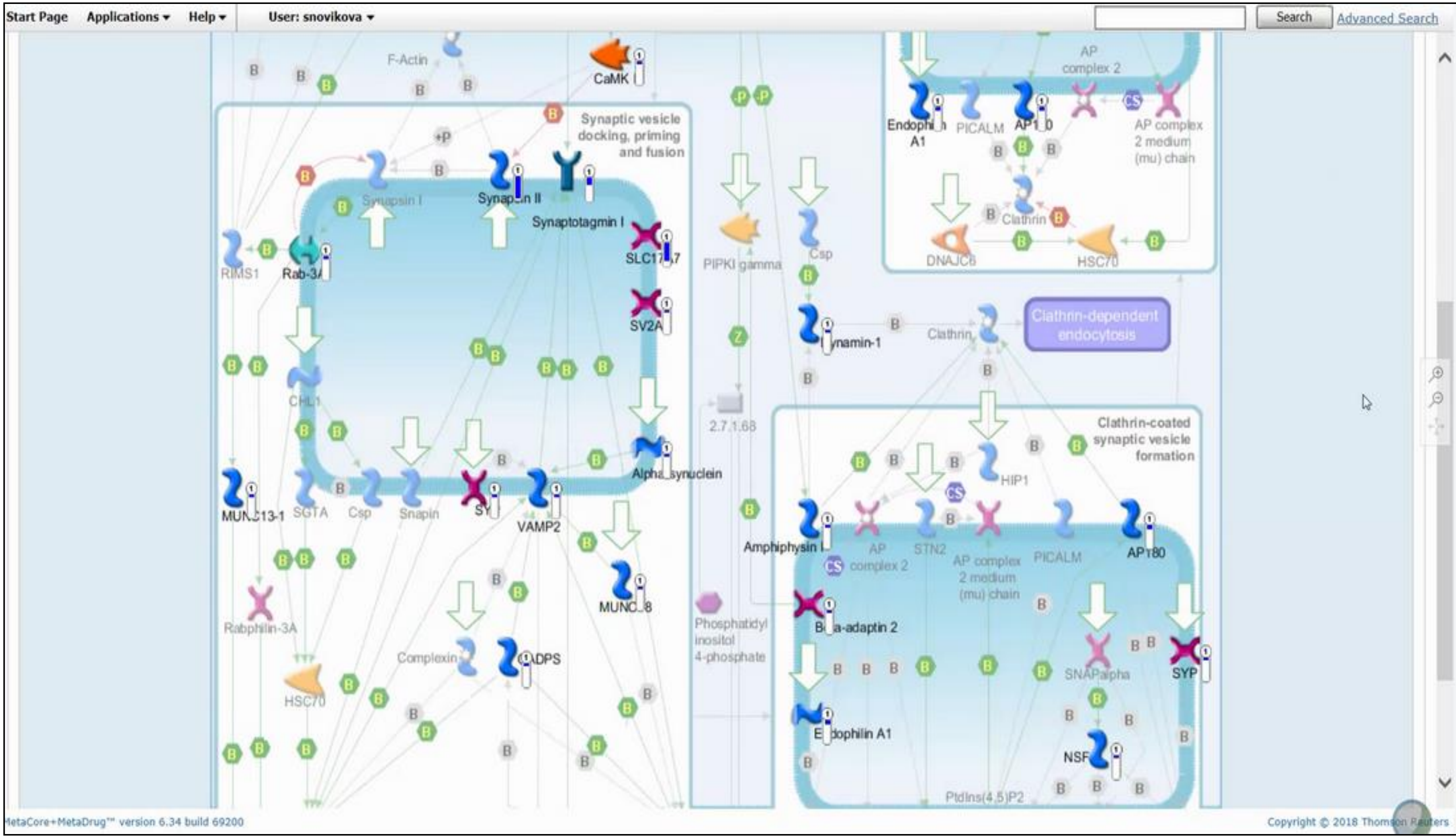

Übersicht MetaCore

Start Page Applications ▾ Help ▾ User: snovikova ▾ Search Advanced Search

Map | Overview | Processes

Keratin filaments

Cytoskeleton in most eukaryotic cells consists of three distinct, yet interconnected, filament systems: **Actin** filaments, **Microtubules** and intermediate filaments. Cell assembly is integrated by the network of intermediate filaments (IFs) and by their interactions with other cytoskeleton structural elements defining cytoarchitecture and cytodynamics.

IF network is critically involved in cell shape control and imparts intracellular mechanical strength. The family of IF proteins has five sub-families. Four of the sub-families are localized in the cytoplasm, whereas the fifth sub-family is found in the nucleus. Expression patterns of cytoplasmic IFs are cell- and tissue-type specific.

The main IF protein is found in epithelial cells is **Keratin** and in fibroblasts it is **Vimentin**. **Keratin** IFs are obligate co-polymers of acidic and basic cytokeratins [1], [2].

IF networks are cross-linked by special binding proteins, e.g., **Plectin**, **BPAG1**, **Desmoplakin**, **Envoplakin**, **Periplakin**, **Epiplakin, Trichoplein** and **Plakophilins** [3], [4], [5], [6]. These proteins maintain cell and tissue integrity by coordinated interconnection of three distinct cytoskeletal filament systems, and anchoring them to membrane complexes.

Assembly, disassembly and subcellular organization of IFs is regulated by kinases, e.g., by Mitogen-activated protein kinases 8-10 (**JNK(MAPK8-10)**) and Cell division cycle 2(**CDK1**) [7], [8].

Objects list:

14-3-3 gamma	14-3-3 protein gamma
Actin cytoskeletal	Actin cytoskeletal *Protein group*
BPAG1	Dystonin
CDK1 (p34)	Cyclin-dependent kinase 1
Desmoplakin	Desmoplakin
Envoplakin	Envoplakin
Epiplakin	Epiplakin
GRB2	Growth factor receptor-bound protein 2
JNK(MAPK8-10)	c-Jun N-terminal kinases *Protein group*
Keratin 1	Keratin, type II cytoskeletal 1
Keratin 13	Keratin, type I cytoskeletal 13
Keratin 14	Keratin, type I cytoskeletal 14
Keratin 16	Keratin, type I cytoskeletal 16

MetaCore+MetaDrug™ version 6.34 build 69200

Copyright © 2018 Thomson Reuters

MetaCore: Legende

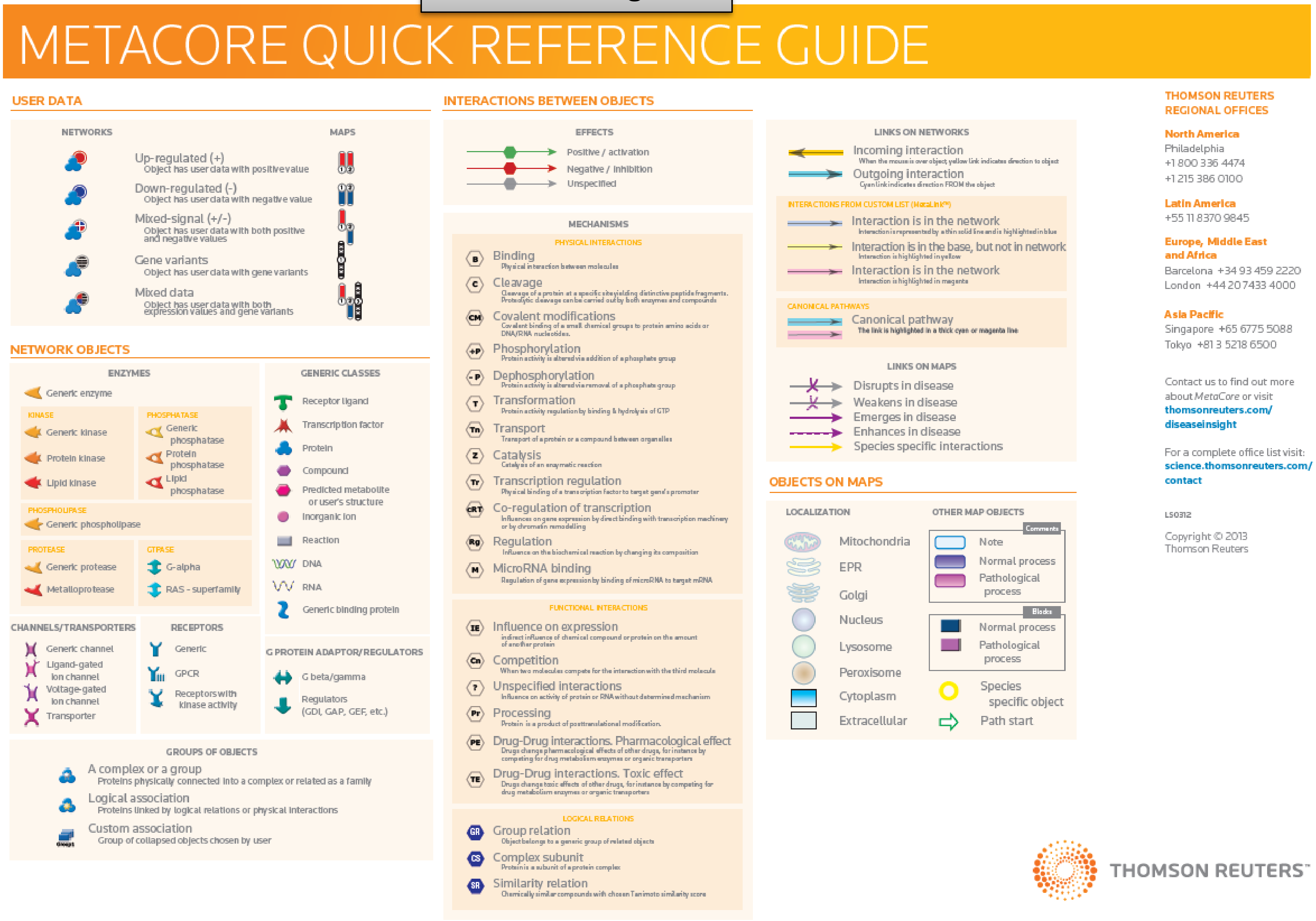

Kurzbeschreibung aus der Clarivate Sales Powerpoint-Präsentation

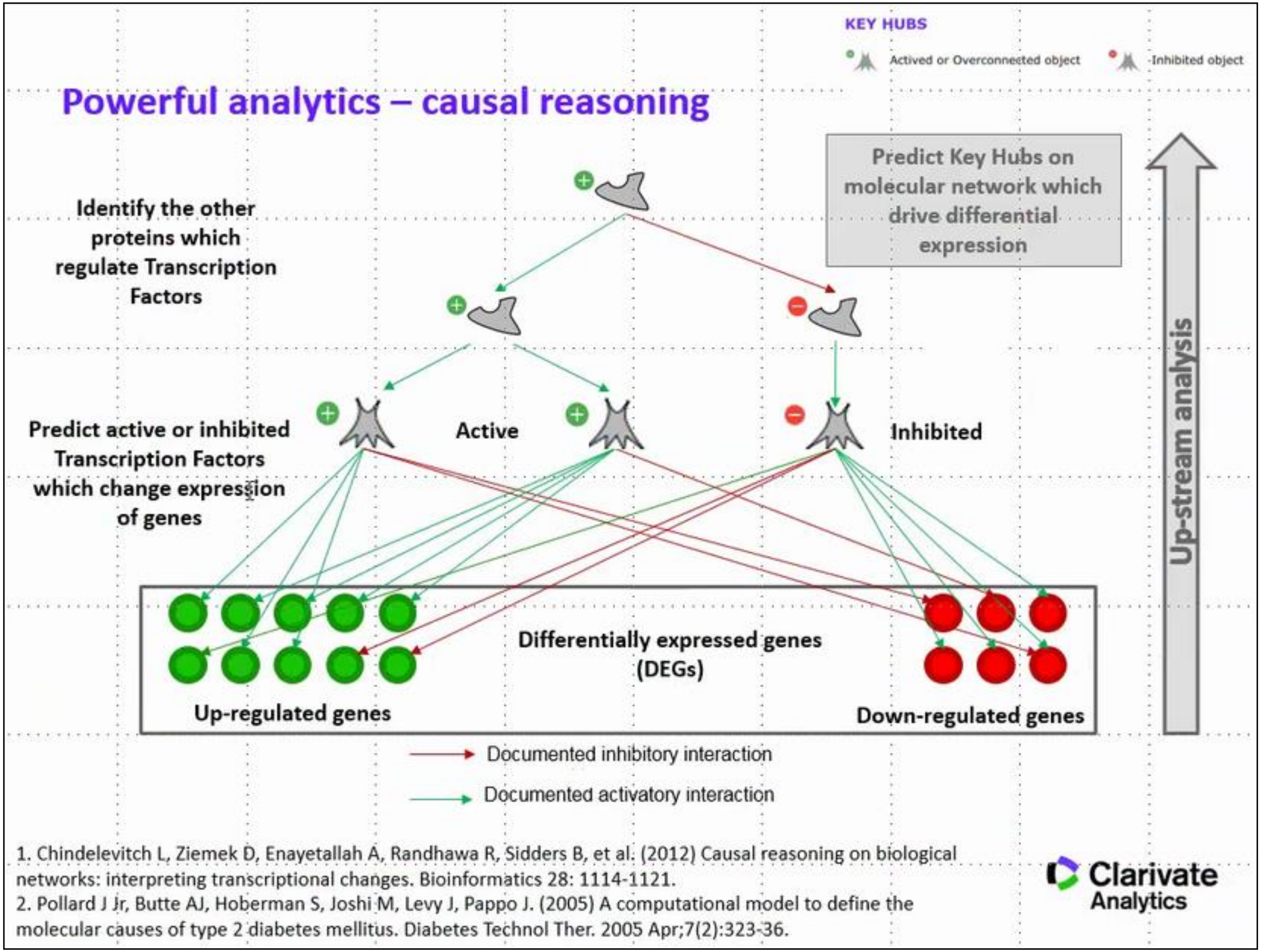

Key Pathway Advisor: Entities <-> Interactions

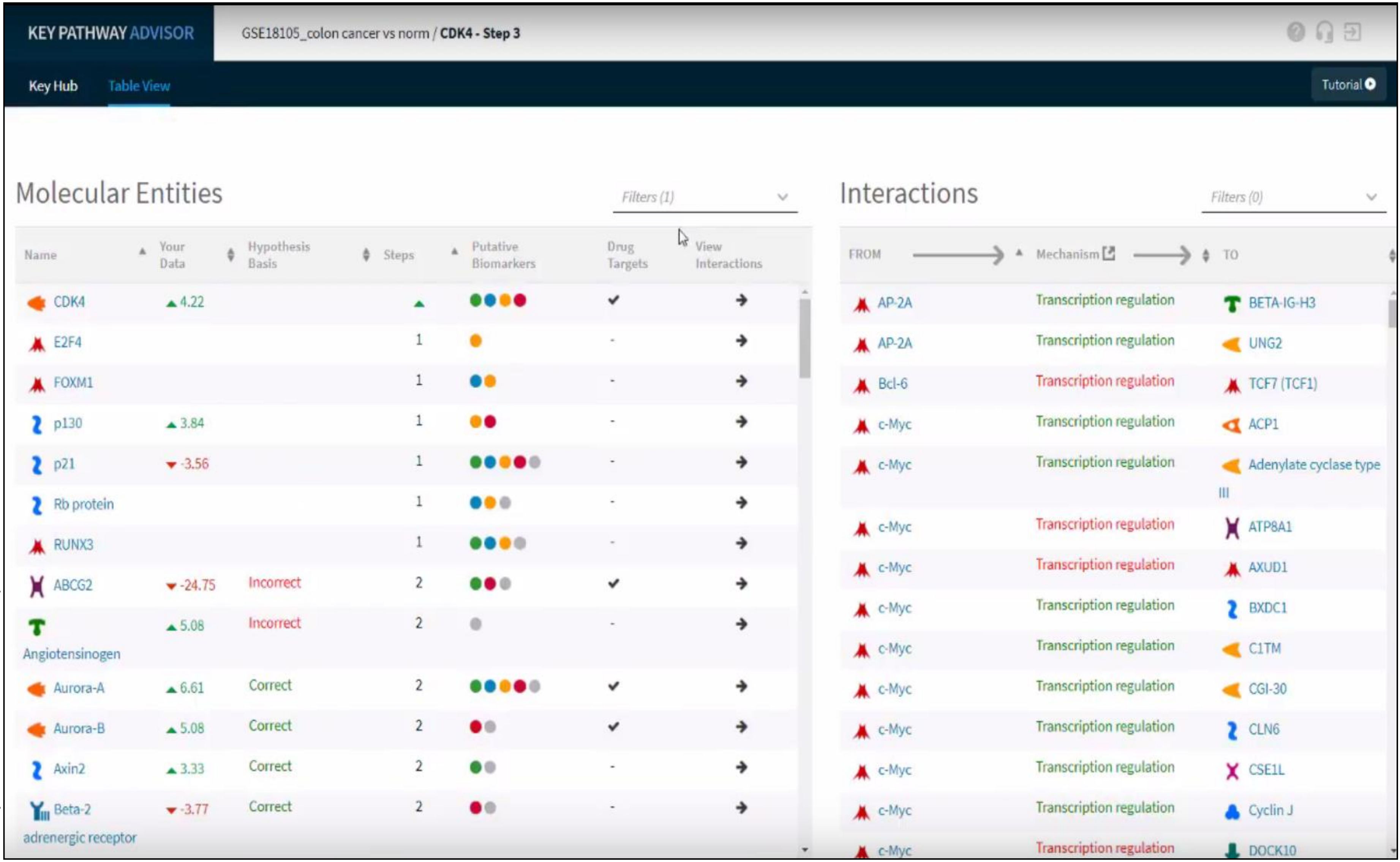

Key Pathway Advisor: Putative Biomarkers

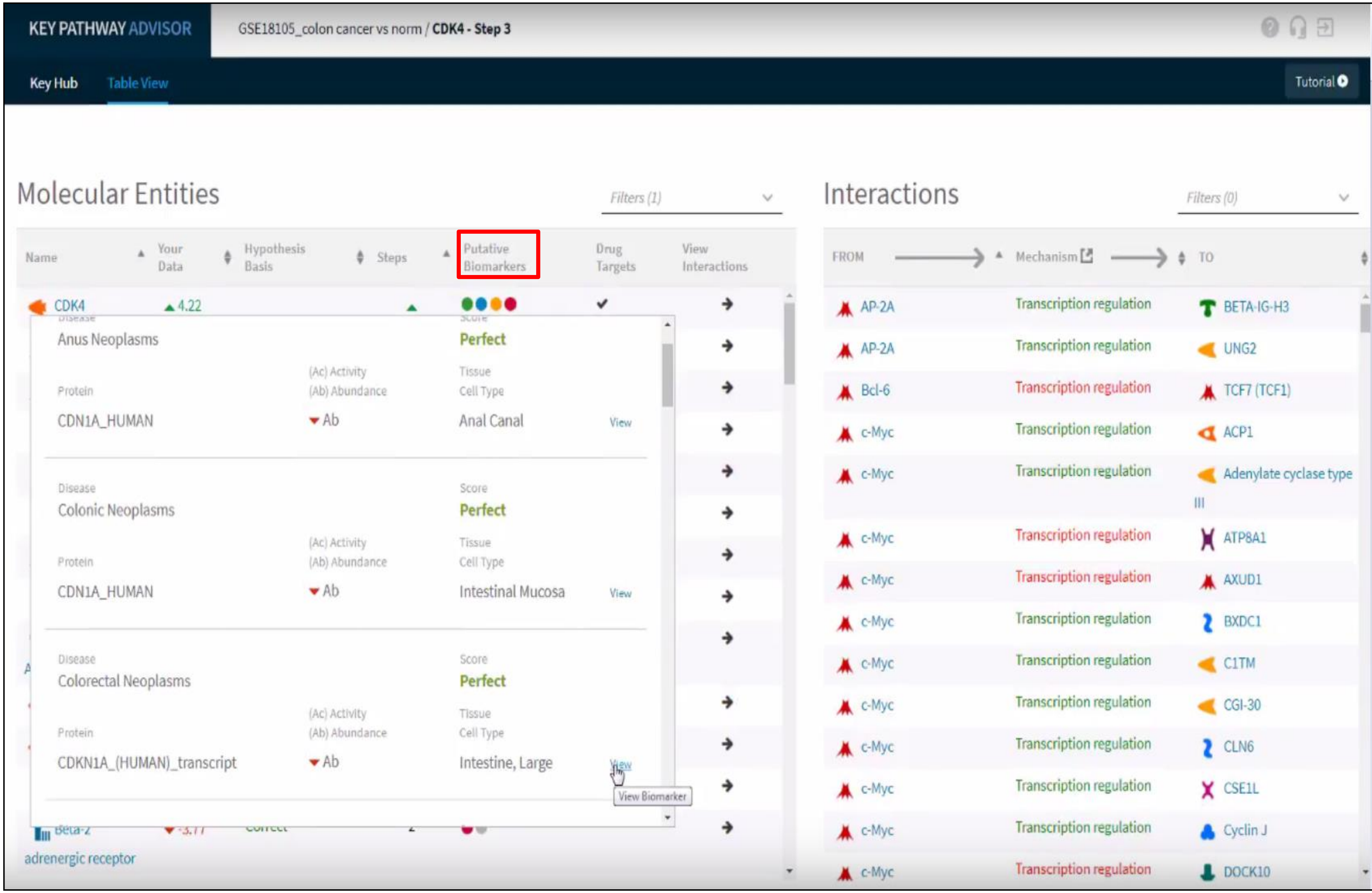

Key Pathway Advisor: Putative Biomarkers

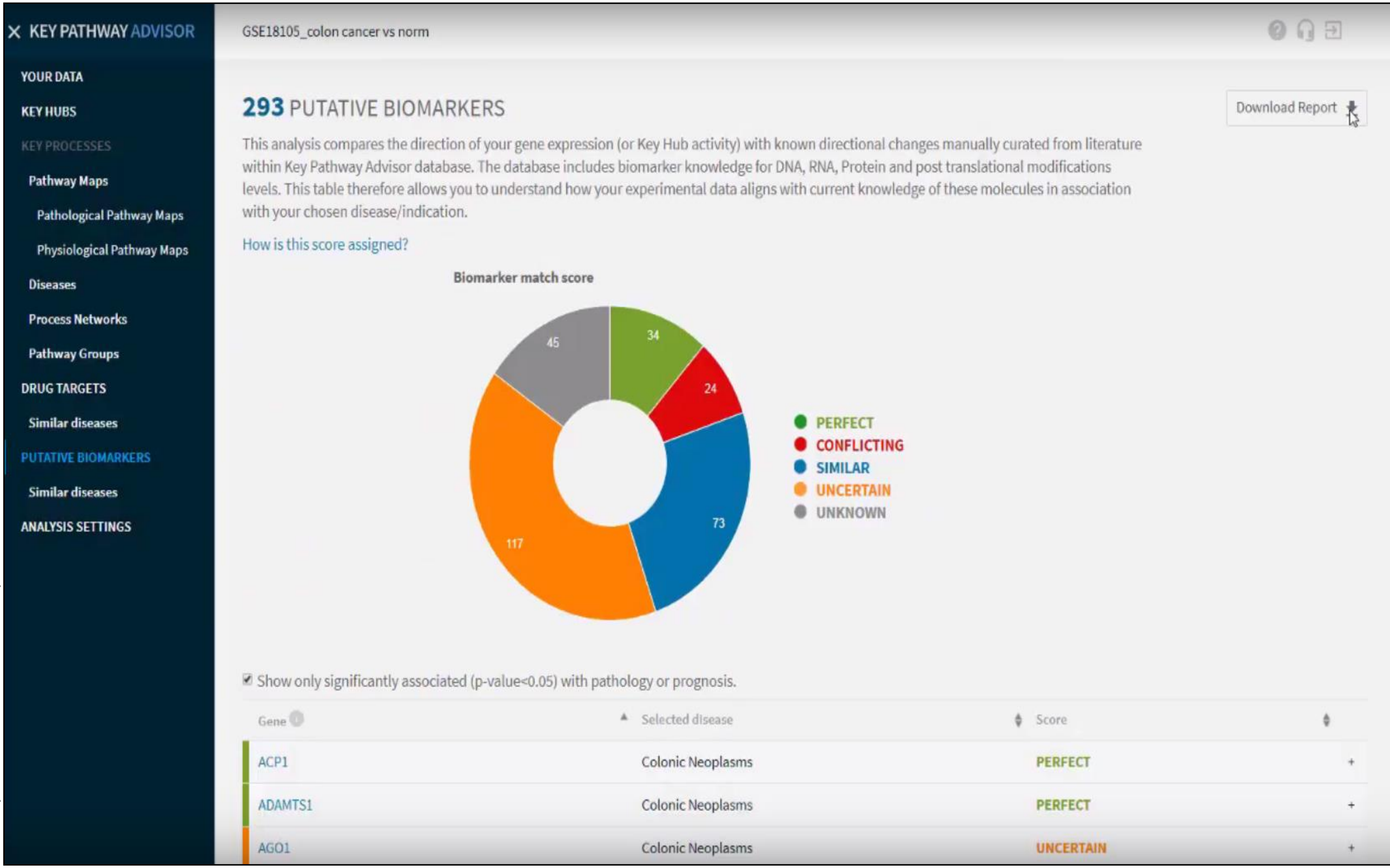

Key Pathway Advisor: Key Hub

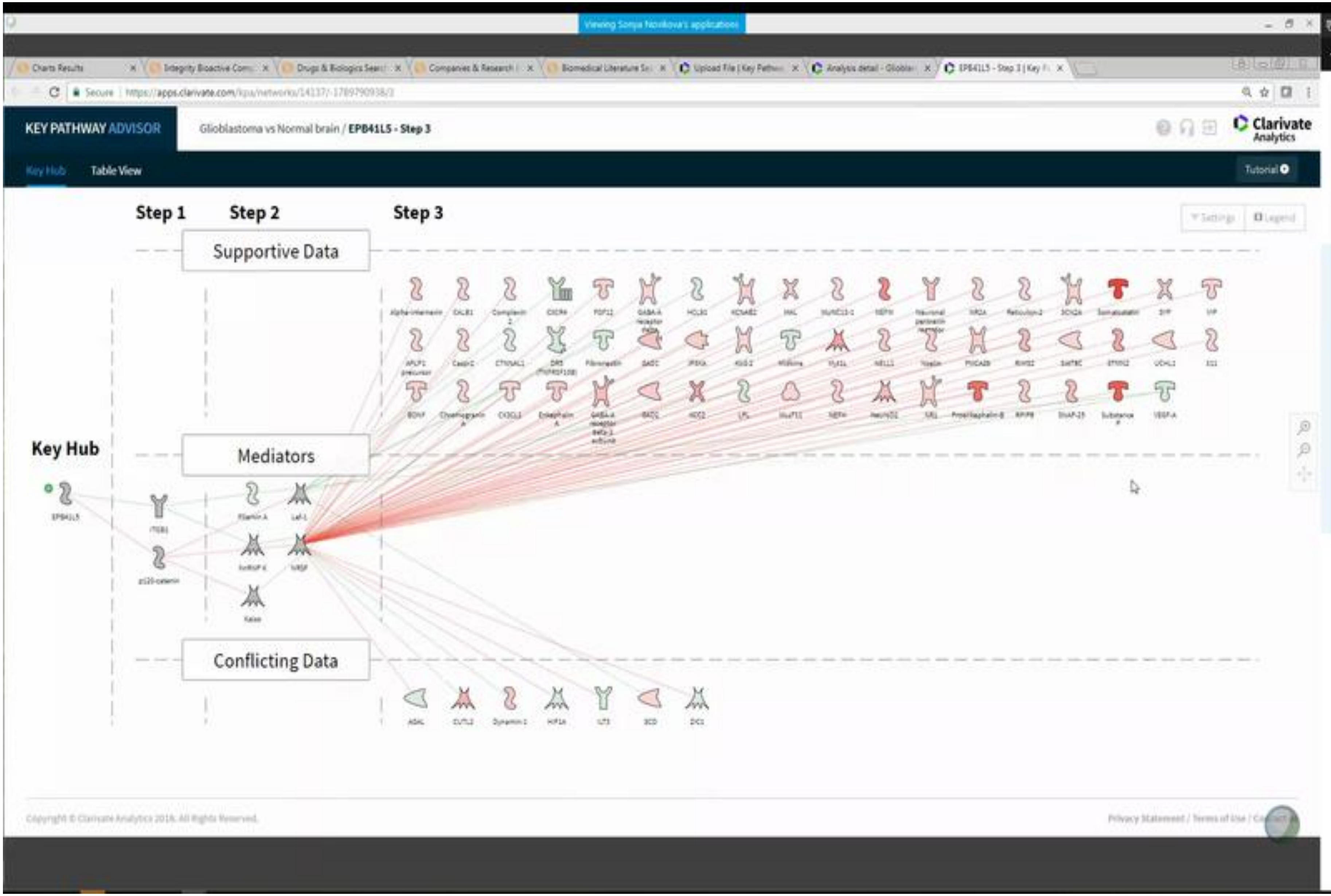

4. COMPASS™ for My Geisinger

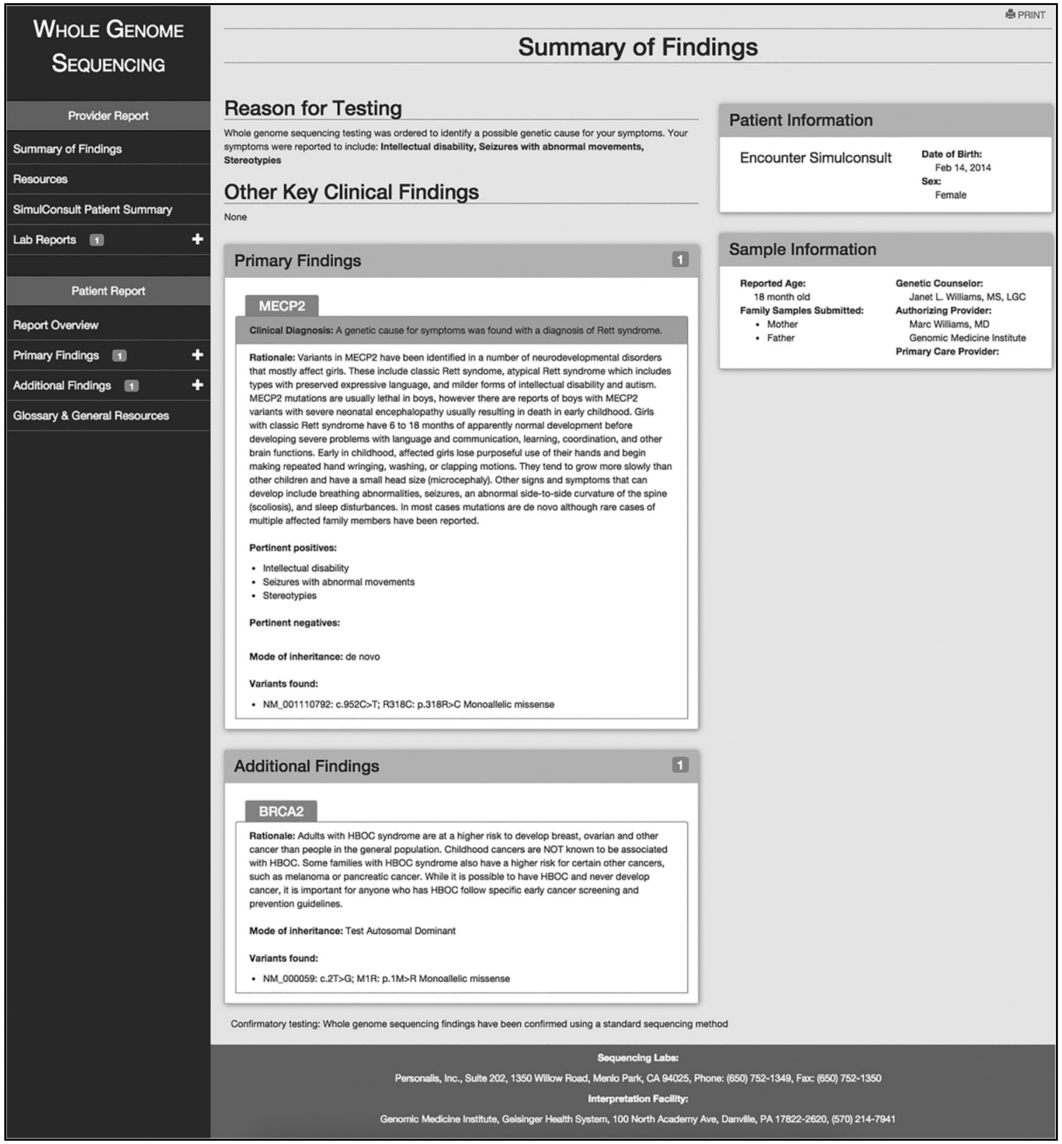

WHOLE GENOME SEQUENCING

Provider Report
- Summary of Findings
- Resources
- SimulConsult Patient Summary
- Lab Reports 1

Patient Report
- Report Overview
- Primary Findings 1
- Additional Findings 1
- Glossary & General Resources

PRINT

Summary of Findings

Reason for Testing

Whole genome sequencing testing was ordered to identify a possible genetic cause for your symptoms. Your symptoms were reported to include: **Intellectual disability, Seizures with abnormal movements, Stereotypies**

Other Key Clinical Findings

None

Patient Information

Encounter Simulconsult

Date of Birth: Feb 14, 2014
Sex: Female

Sample Information

Reported Age: 18 month old
Family Samples Submitted:
- Mother
- Father

Genetic Counselor: Janet L. Williams, MS, LGC
Authorizing Provider: Marc Williams, MD, Genomic Medicine Institute
Primary Care Provider:

Primary Findings 1

MECP2

Clinical Diagnosis: A genetic cause for symptoms was found with a diagnosis of Rett syndrome.

Rationale: Variants in MECP2 have been identified in a number of neurodevelopmental disorders that mostly affect girls. These include classic Rett syndrome, atypical Rett syndrome which includes types with preserved expressive language, and milder forms of intellectual disability and autism. MECP2 mutations are usually lethal in boys, however there are reports of boys with MECP2 variants with severe neonatal encephalopathy usually resulting in death in early childhood. Girls with classic Rett syndrome have 6 to 18 months of apparently normal development before developing severe problems with language and communication, learning, coordination, and other brain functions. Early in childhood, affected girls lose purposeful use of their hands and begin making repeated hand wringing, washing, or clapping motions. They tend to grow more slowly than other children and have a small head size (microcephaly). Other signs and symptoms that can develop include breathing abnormalities, seizures, an abnormal side-to-side curvature of the spine (scoliosis), and sleep disturbances. In most cases mutations are de novo although rare cases of multiple affected family members have been reported.

Pertinent positives:
- Intellectual disability
- Seizures with abnormal movements
- Stereotypies

Pertinent negatives:

Mode of inheritance: de novo

Variants found:
- NM_001110792: c.952C>T; R318C: p.318R>C Monoallelic missense

Additional Findings 1

BRCA2

Rationale: Adults with HBOC syndrome are at a higher risk to develop breast, ovarian and other cancer than people in the general population. Childhood cancers are NOT known to be associated with HBOC. Some families with HBOC syndrome also have a higher risk for certain other cancers, such as melanoma or pancreatic cancer. While it is possible to have HBOC and never develop cancer, it is important for anyone who has HBOC follow specific early cancer screening and prevention guidelines.

Mode of inheritance: Test Autosomal Dominant

Variants found:
- NM_000059: c.2T>G; M1R: p.1M>R Monoallelic missense

Confirmatory testing: Whole genome sequencing findings have been confirmed using a standard sequencing method

Sequencing Labs:
Personalis, Inc., Suite 202, 1350 Willow Road, Menlo Park, CA 94025, Phone: (650) 752-1349, Fax: (650) 752-1350
Interpretation Facility:
Genomic Medicine Institute, Geisinger Health System, 100 North Academy Ave, Danville, PA 17822-2620, (570) 214-7941
