# Supplementary material for: Requirements Analysis and Specification for a Molecular Tumor Board Platform Based on cBioPortal
Source: Diagnostics (Basel). 2020 Feb 10;10(2):93. doi: 10.3390/diagnostics10020093 (PMC7167859; doi:10.3390/diagnostics10020093)
Supplement: Supplementary file 1 [file diagnostics-10-00093-s001.zip › Supplementary File 5_Guideline with requirements and their options for second round of interviews.pdf]

Supplementary File 5: Guideline with requirements and their options for second round of interviews.

We conducted the interviews in German. This is a translation of the guideline we created and used in the second round of interviews. Since we hosted this guideline in our team collaboration software, the feature to view a mockup in full-size-view by clicking on it is not available in this document. For full-size-view of the screenshot mockups see Supplementary File 8.

1. Meta data about sample

This section deals with the documentation of the exact localization of the samplings taken and the respective analysis methods (WES, etc.).

Display of the localization of the tissue sample

Description	Notes	Mockup (click for full-size-view)
For each stored sample the corresponding localization of the sampling shall be displayed.		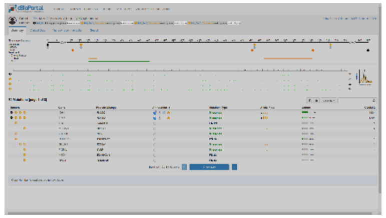

Display the analysis method

Description	Notes	Mockup (click for full-size-view)

The corresponding analysis method should be displayed for each stored sample.		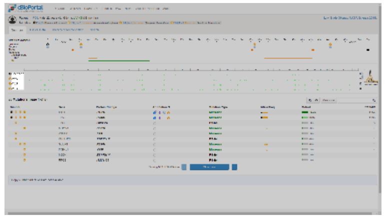

2. Scores, MAF and cohort representation

cBioPortal already offers a selection of annotations for individual mutations. This topic deals with the integration of further annotation and population databases (e.g. gnomAD) as well as the presentation of other information about the individual mutations (e.g. allele frequency or coverage).

Scores

The display of the scores listed below may be useful for evaluating the results and thus positively influence the finding of a therapy recommendation.

	**Description**	**Notes**	**Mockup (click for full-size-view)**
Condel score	Indicating for each mutation a "D" (deleterious) or "N" (neutral) based on Condel score.		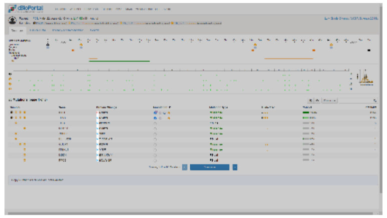
Phred quality score	Display a phred quality score quantifying the accuracy of base calling by the sequencer.		

Minor Allele Frequencies (MAF)

Data from population databases may provide important insights about an identified mutation. In order to retrieve this information effectively and easily, it may be necessary to integrate corresponding databases.

	Description	Notes	Mockup (click for full-size-view)
gnomAD	Example on gnomad.broadinstitute.org (gnomAD)<br>• Exome and genome data<br>• Successor project of ExAC		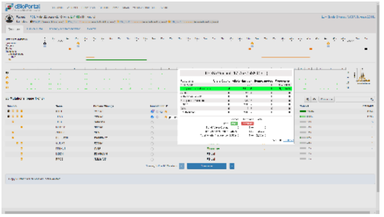
ExAC	Example on exac.broadinstitute.org (ExAC)<br>• Exome data only<br>• Predecessor of gnomAD		
dbSNP	Example on ncbi.nlm.nih.gov (dbSNP)		

Cohort representation

cBioPortal already offers the possibility of classifying a mutation into the overall context of the study cohort. However, it remains to be clarified which cohort should be used as a reference for use in MTBs.

Cohort	Description	Notes	Mockup (click for full-size-view)

Local (based on patient's tumor entity)	All patients registered at the site in cBioPortal with the same tumor entity are defined as a cohort.		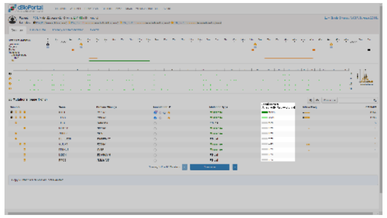
Local (all tumor entities)	All patients registered at the site in cBioPortal are defined as a cohort (independent of the actual tumor entity).		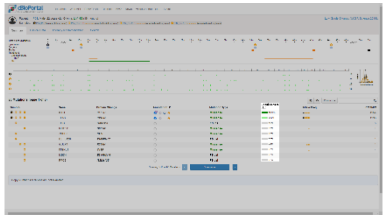
MIRACUM (based on patient's tumor entity)	All patients with the same tumor entity registered across all MIRACUM sites are defined as a cohort.		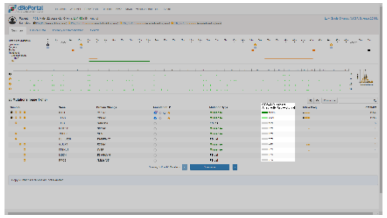
MIRACUM (all tumor entities)	All patients registered across all MIRACUM sites are defined as a cohort (independent of the actual tumor entity).		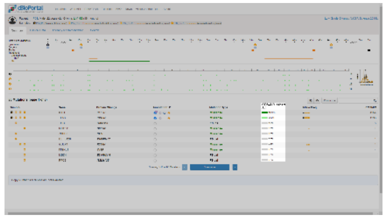
Worldwide (based on patient's tumor entity)	A worldwide cohort should be formed from various studies with patients suffering from a specific tumor entity. Possible data could originate from the public cBioPortal instance.		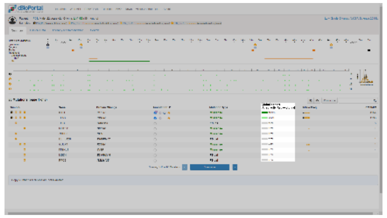

Worldwide (all tumor entities)	A worldwide cohort should be formed from various studies (independent of the actual tumor entity). Possible data could originate from the public cBioPortal instance.		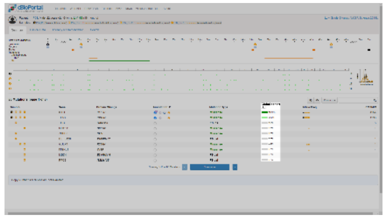

3. TMB, MIRACUM occurrences, pathway analysis

If the burden of mutations is particularly high or if the current case is a borderline type, users may require features like

- calculation of Total Mutational Burden,
- registration of occurrences of mutations in the consortium or
- analyzation of pathways.

Total Mutational Burden

To get an overview of the total mutational load of the tumor, it is recommended to calculate the Total Mutational Burden [Mutations / Mb].

	**Description**	**Notes**	**Mockup (click for full-size view)**
TMB	The TMB should be displayed without comparisons with values of other patients with the same tumor entity.		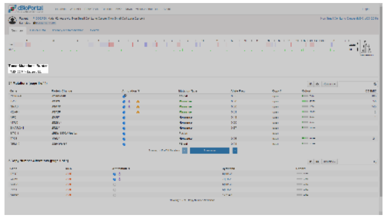

TMB in relation to tumor entity	The TMB should be placed in the overall context of the respective tumor entity. This may be done by displaying a boxplot representation.		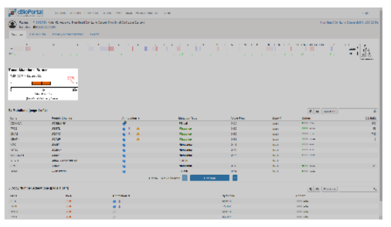

MIRACUM occurrences

This feature should provide a search for sites that have already identified a particular mutation in one of their patient samples (MIRACUM occurrences) and a way to contact the corresponding clinician or to view relevant details about the patient.

	**Description**	**Notes**	**Mockup (click for full-size view)**
MIRACUM occurrences including details about patient	For each mutation it should be indicated if and how often it has already been identified at other MIRACUM sites. Furthermore, in addition to providing contact details, access to relevant patient information (such as therapy recommendations) should be given.		
MIRACUM occurrences including contact details	For each mutation it should be indicated if and how often it has already been identified at other MIRACUM sites. Furthermore, contact details should be provided.		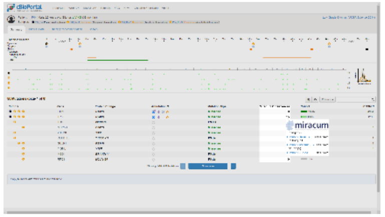
Beacon network	For each mutation, it should be indicated whether and how often it has already been identified in samples worldwide (not limit to MIRACUM sites). A database like [Beacon Network](#) may be used.		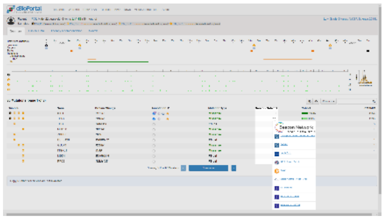

	Description	Notes	Mockup (click for full-size view)
Subdivided by tumor entity	The total number of occurrences per site should be divided into the respective tumor entities.		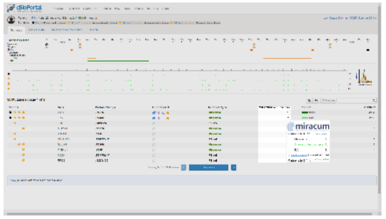
Display only total count	Only the total number of all occurrences should be displayed for each site.		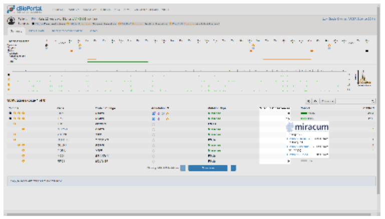

	Description	Notes
Count all occurrences	All mutations should be counted without exception.	
Count occurrence only if mutation is relevant for therapy recommendation	Only mutations that have been classified as relevant for therapy should be counted.	

Pathway analysis

The visualization of molecular pathways may be an important tool to link individual mutations to molecular function and pathway.

	Description	Notes	Mockup (click for full-size view)

Map	There should be a graphical representation in the form of a map with genes involved in a pathway and corresponding mutations should be highlighted there.		
Grouping in mutation table	In the mutation table a further column should be displayed indicating the pathways affected by the corresponding mutation. The different pathways should be differentiated.		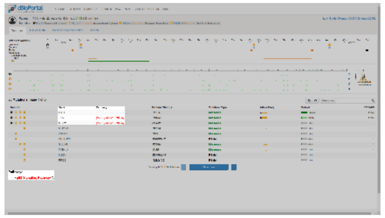 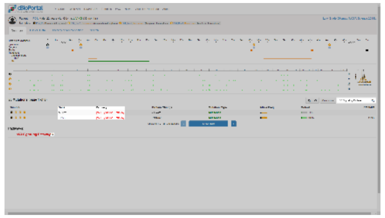

4. Visualization of CNV and methylation data

This topic deals with the visualization of duplicated and eliminated gene segments, as well as with the necessity of visualizing DNA methylation data.

Visualization of CNV data

In cBioPortal there is already a way to display Copy Number Variations (Copy Number Alterations).

	**Description**	**Notes**	**Mockup (click for full-size view)**
Like in Sophia Genetics	The CNV visualization should be implemented similar to the visualization in Sophia Genetics.		

other	The CNV visualization should be implemented in a different (not yet specified) way (compared to Sophia Genetics and cBioPortal)		
cBioPortal's current implementation	No change to current visualization in cBioPortal necessary.		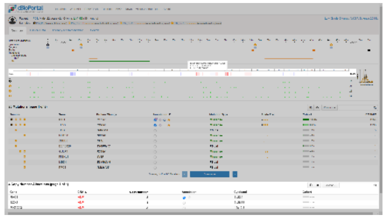

	Description	Notes	Mockup (click for full-size view)
Group display by gene	Group CNV data by gene.		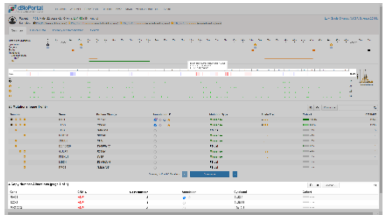
Group display by chromosome	Group CNV data by chromosome and display total amplifications / deletions per chromosome.		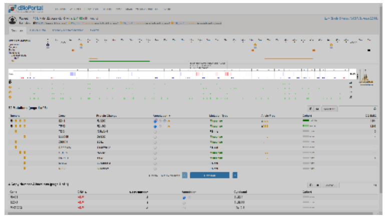
Group display by genome	Do not group CNV data but display total amplifications / deletions (all chromosomes / whole genome)		

Visualization of methylation data

Visualization of the methylation of a DNA section to detect abnormalities and deviations.

**Description**	**Notes**
cBioPortal should provide a function for the visualization of methylations.	

5. Classification of relevant mutations

There are several ways to classify the pathogenicity of a particular mutation. The goal is to reach agreement on a classification and to discuss the potentials of a (semi-)automatic application of this classification. In addition, a way for marking the relevant mutations must be defined.

JAX-CKB

JAX-CKB (Clinical Knowledge Base) provides information on mutations and literature references as well as information on possible drug therapies.

**Description**	**Notes**	**Mockup (click for full-size view)**
The database of JAX-CKB should be integrated into cBioPortal and the corresponding information should be directly accessible.		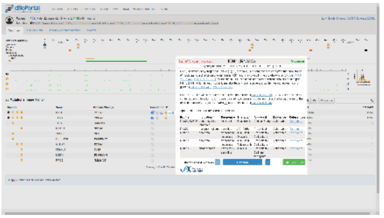

ClinVar

ClinVar provides information about the connection of a mutation to corresponding phenotypes.

**Description**	**Notes**
If an entry for a mutation can be found in ClinVar, it should be displayed in cBioPortal.<br><br>Example: TP53 (R248Q)	

MyCancerGenome

MyCancerGenome provides further information on mutations and assigns them to different diseases (currently 25). Among other things, it provides general information on mutations and direct hyperlinks to relevant abstracts.

MyCancerGenome has already been integrated into cBioPortal to the extent that direct hyperlinks to the corresponding entries for a mutation listed in MyCancerGenome are provided.

Example: BRAF-Mutation (V600M) - Melanom

	**Description**	**Notes**	**Mockup (click for full-size view)**
Current integration in cBioPortal is sufficient	As described above, cBioPortal currently displays a list of relevant hyperlinks to the entries in the MyCancerGenome database; further information is not extracted.		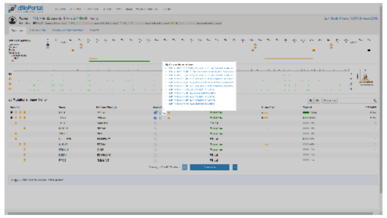
Improved integration of MyCancerGenome	The data provided by MyCancerGenome should be displayed directly in cBioPortal similar to the other annotations, so that the detour via the hyperlink call is no longer necessary.		

Decision making (regarding pathogenicity)

This is about the general way of classification process (automatic, semi-automatic, manual).

	Description	Notes
Automatically	The classification of a mutation with regard to its pathogenicity should be fully automated on the basis of suitable criteria. A manual intervention / correction is not planned.	
Semi-automatically	The generally automatic classification of a mutation should be checked manually. This would allow the appropriate specialist personnel to mark the correctly classified mutations and correct any errors manually.	
Manually	Each mutation has to be classified manually.	

Pathogenicity classification

This is about deciding in which categories individual mutations should be classified with regard to their pathogenicity.

	Description	Notes	Mockup (click for full-size view)
Pathogenicity based on level A-D	• A: Highly pathogenic<br>• B: Potentially pathogenic<br>• C: Unknown significance<br>• D: Likely benign		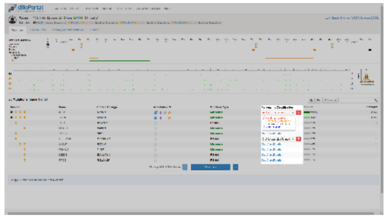<br>Mockup: manual classification

Pathogenicity based on level 1-5	• 5: Highly pathogenic<br>• 4: Potentially pathogenic<br>• 3: Unknown significance<br>• 2: Likely benign<br>• 1: Benign		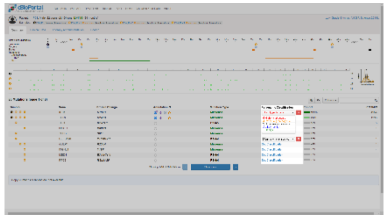<br>Mockup: manual classification

Therapy relevant mutations

A final therapy recommendation must be substantiated. In the given context, it may be relevant to mark certain mutations on the basis of which a recommendation will later be made.

	**Description**	**Notes**	**Mockup (click for full-size view)**
Select only a single mutation	The selection of a single mutation is sufficient to provide a therapy recommendation.		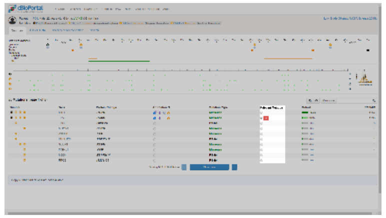
Select multiple mutations	The preparation of a therapy recommendation may require a selection of several mutations.		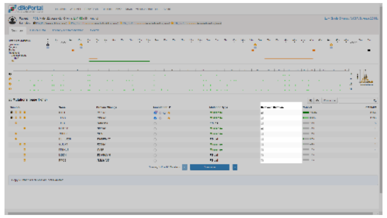

Select multiple mutations including short free text field	The preparation of a therapy recommendation may require a selection of several mutations. It should also be possible for each mutation to add a short free text.		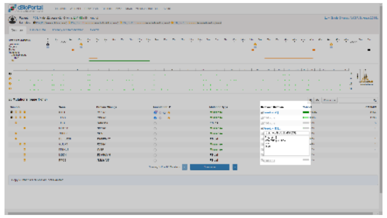

Additional flags for mutations

Certain mutations are known to predict a specific tumor entity. They can also be used to diagnose or confirm that entity. In order to document these three properties, a new function in cBioPortal is required.

Description	Notes	Mockup (click for full-size view)
It should be possible to mark a mutation on the basis of the following three characteristics:<br>• predictive<br>• diagnostic<br>• confirmatory		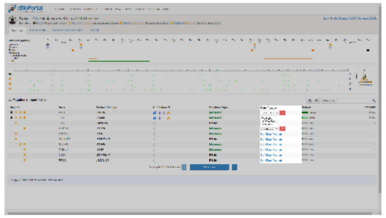

6. Search tool for similar patients

This requirement is about the ability to easily and accurately find patients who have already been treated and whose characteristics match those of the current patient to a certain degree.

Radius of search

It must be decided whether only local patients should be listed, or whether a MIRACUM-wide search should be provided by cBioPortal.

Note: This was not explicitly mentioned in the first round of interviews.

	Description	Notes
Local only	The search should include only local patients and not patient data from other locations.	
MIRACUM-wide	The search should be MIRACUM-wide and thus also include patients from other locations.<br><br>Example:<br><br>If an MTB member at location A searches for similar patients with mutations X and Y, not only the patients registered at location A with these mutations will be displayed, but also those who have already been registered at location B / C / etc. and also meet the search criteria defined by the user.	

Search parameters

The search is based on mutations previously marked as relevant for therapy recommendation.

Search for ...	Description	Notes	Mockup (click for full-size view)
... tumor entity	Only list similar patients that also match in terms of the tumor entity.		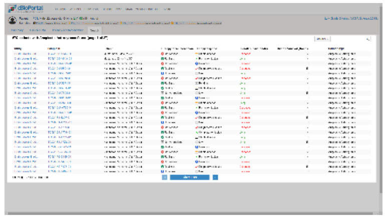
... mutation affecting the same gene	Limit search results to mutations in the same gene (regardless of type and localization) and to patients with the same clinical picture.		

... single mutation	Only a single mutation is included in the search.	• Is already supported by cBioPortal via detours	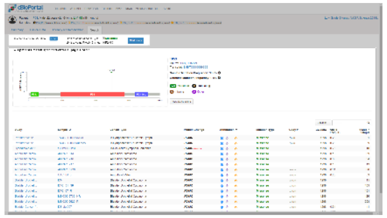
... pattern of mutations (match exactly)	The search results should be limited to an exact mutation pattern.<br><br>For example, only patients who all have a specific mutation A and also a mutation B will be considered.		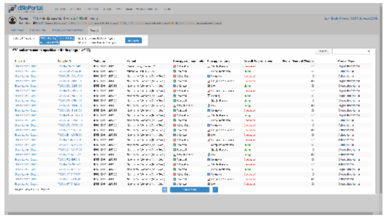
... pattern of mutations	The search results should be limited to a mutation pattern. It is also possible to search for patterns that do not exactly match the pattern of the current patient.<br><br>For example, only patients with a specific mutation A and simultaneously a mutation B and C are considered.<br><br>If, for example, the coverage rate of the search process is set to 2 out of 3 mutations, patients with only mutations A and B / A and C / C and B will also be considered.		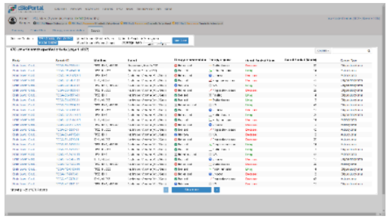

7. Extension of data provided by OncoKB

OncoKB is already represented with some functions in cBioPortal. For the use in the MIRACUM project however still some additional functions are necessary, in order to be able to use the made available data effectively also in Germany.

Display basis

Currently, OncoKB offers a list of drugs that could be suitable for a particular mutation. It needs to be clarified whether the focus of this recommendation should rather be on the tumor entity or on the corresponding mutation.

**Display grouped by...**	**Description**	**Notes**
...mutation	As it is currently solved in cBioPortal, the list of drugs for each mutation listed in OncoKB will be displayed individually if the sample of the patient contains this mutation.	
...entity	The data from OncoKB will be used to list all drugs that are suitable for the respective tumor entity regardless of the mutation(s) present.<br><br>Example:<br><br>A patient suffers from NSCLS. If a drug X is suitable for the therapy of an NSCLS with mutation A, the drug X is also proposed if the sample of the patient does not have mutation A at all.	

Approval status

There is currently no function in cBioPortal that checks a drug proposed by OncoKB for approval in Germany.

**Approval status...**	**Description**	**Notes**	**Mockup (click for full-size view)**

... for each drug	If a drug is already approved for any tumor entity, the drug should always be displayed as approved.<br><br>For example, a drug that has been approved for a tumor entity A is always displayed as approved, even if the patient suffers from a tumor of entity B.		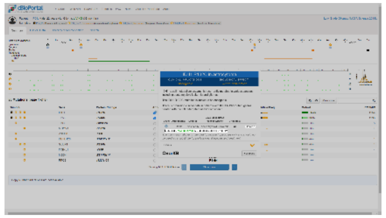
... for each drug according to tumor entity	The entity should also be taken into account when displaying the approval status of a drug.<br><br>If a drug is only approved for the therapy of a tumor entity A and B, this information should also be displayed.		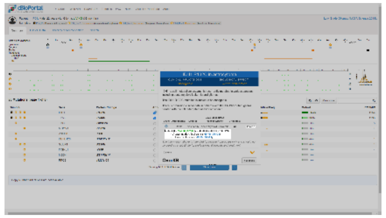

	Description	Notes
Propose drug based on evidence level	A drug should only be indicated as approved if the corresponding therapy exceeds a certain level of evidence.<br><br>For example, a drug A with evidence level IIb could still be indicated as approved, but a drug B with evidence level III might no longer be indicated.	
Always propose drug	Regardless of the level of evidence of the therapy, a drug should always be indicated as approved (provided, of course, that it is actually approved).	

Approving authority

Some of the sites requested to mark the responsible authority in addition to the actual approval status.

Description	Notes	Mockup (click for full-size view)
If a drug is indicated as approved, the authority responsible for the approval should also be displayed.		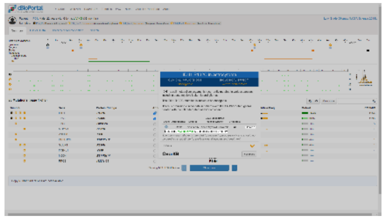

8. Search tool for suitable studies

With the services offered by clinicaltrials.gov, it is possible to find suitable studies for the current patient and his clinical picture and to obtain further information about them.

Search parameters

So far, the potential parameters listed below for the search for a suitable study have been determined. When using clinicaltrials.gov as a database, synonyms / acronyms are also considered. Thus, when searching for studies with the drug "Lomustine", those will also be found which merely refer to the corresponding acronym "CCNU".

Search for ...	Description	Notes	Mockup (click for full-size view)
... drug	The search results can be limited to a specific drug.<br>For example, you can search for studies with the drug "Lomustine".		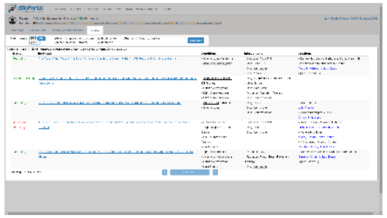
... mutation	The search results can be limited to one or more mutations.<br>The selection of the mutations is made from the mutations already marked as relevant for therapy recommendation.		

... tumor entity	The search results can be limited to a specific tumor entity (e.g. anaplastic astrocytoma).		
... clinical data	Further parameters of the patient (e.g. age or gender) can be included in the search.	This was not explicitly mentioned in the first round of interviews.	

9. Clinical data

In principle, it is already possible to integrate the clinical data of patients into cBioPortal. However, it is not yet clear how such a data set has to be designed (e.g. age, gender, etc.) in order to be used within a molecular tumor board.

Also, if the integration of clinical data becomes necessary, it must be determined whether cBioPortal should play a parallel (with identical data) or even a supplementary role in relation to other systems in the HIS.

Integration into HIS

	**Description**	**Notes**
No usage of clinical data at all	The collected clinical data of the patient are not stored in cBioPortal. Only the locally available components of the HIS are used for this purpose.	
Identical data (compared to other systems in the HIS)	The collected clinical data of the patient are also stored/displayed in cBioPortal parallel to other systems in the HIS.<br>However, much more data will be stored in the other systems of the HIS and only relevant data will be displayed in cBioPortal, too.	

Additional data (compared to other systems in the HIS)	The collected clinical data of the patient are also stored/displayed in cBioPortal. cBioPortal may contain certain data which are not stored in other systems of the HIS.<br>However, much more data will be stored in the other systems of the HIS and only relevant data will be displayed in cBioPortal, too.	

10. Therapy recommendation

The topic of the therapy recommendation is very broad and requires some important decisions. These include the type of input, the range (which location may see which content?), the content and documentation of the underlying reason of the recommendation.

Type of input

In principle, there are three ways of entering data. The most widespread method currently used in clinical practice is the recording of free text. The opposite variant is a structured input with fixed values. The third method combines both variants and offers a structured query with both fixed and freely definable values.

	Description	Notes	Mockup (click for full-size view)
free text	The therapy recommendation should be entered unstructured and in free text. This is comparable to writing a letter in established word processing programs.		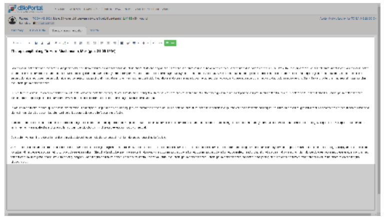

structured	The therapy recommendation should be entered in a structured manner. The user is provided with input masks where he / she can select from predefined values. A free input of text (e.g. drug names) is not provided (and the corresponding name must be taken from a selection of predefined drugs).		
combination	The therapy recommendation should be entered in a structured manner, but the author should be given room for special / unexpected entries (e.g. experimental drug that has not yet been registered).		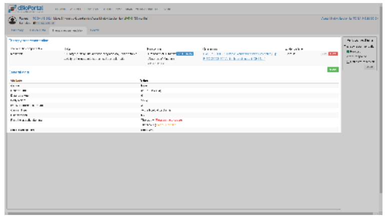 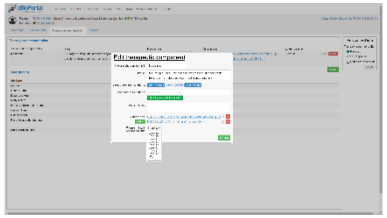

MIRACUM-wide access of therapy recommendations

	Description	Notes	Mockup (click for full-size view)

MIRACUM-wide therapy recommendation	Already documented therapy recommendations of previous patients could help other sites to make their own recommendations. For this purpose, it may be necessary to see the therapy recommendations for previous patients of other partner sites.		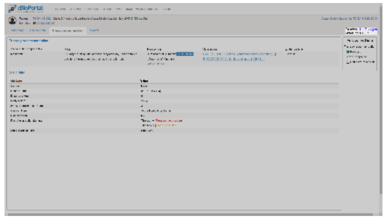

Contents

In order to develop the input mask for the recording of the therapy recommendation, the content requirements for such a recommendation must be determined.

	**Description**	**Notes**	**Mockup (click for full-size view)**
Follow-up data	Particularly in retrospect, it is often interesting to know whether a therapy was ultimately applied at all and what the outcome was. For this purpose, it should be possible to record these follow-up data and display them directly next to the recommended therapy (or somewhere else?).		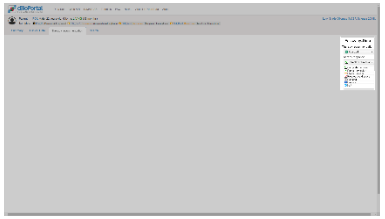
Level of evidence	After an MTB has taken place, it should be possible to record an evidence level in the therapy recommendation. This is particularly useful for drugs combinations of drugs for which clinical research results are available.<br>This level may be independent of the corresponding entries in OncoKB.		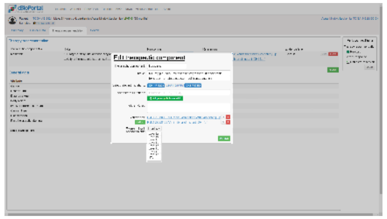
Participation in study	If the patient takes part in a study, this should also be noted in the therapy recommendation.		

Citations	For each therapy recommendation there should also be the possibility to refer to the corresponding literature by means of a hyperlink.		

Generation of text (similar to a doctor's letter)

For the further processing of a therapy recommendation in a doctor's letter it can be advantageous to formulate the therapy recommendation in natural language. For this purpose, the computer could use text modules to provide the user with a pre-formulated therapy recommendation for integration into the letter.

This text could also be included in the PDF report.

**Description**	**Notes**
There should be a function for generating text sections with predefined text modules. For this purpose, the modules are to be automatically supplemented with the corresponding patient parameters.<br>Example:<br>Text module:<br>Mr. %NAME% (%BIRTHDATE%) was presented to the MTB.<br>Generated text:<br>Mr. John Doe (01.01.1970) was introduced to the MTB.	

11. PDF report

The generation of a report in PDF format is intended to provide revision-proof storage of the data contributing to the therapy recommendation. In addition, by displaying all relevant annotations, the report provides ward physicians with a transparent view of the MTB's decision.

Selection of mutations

In order to include the relevant mutations in the report, they must first be selected.

	Description	Notes
manually	The mutations to be included in the report are selected manually when the report is generated. This means, the user can also exclude one or more of the previously selected relevant mutations.	
automatically	The relevant mutations selected before the step of making the therapy recommendation are automatically adopted and cannot be changed for the report.	

Integration of annotation databases

The diversity of the databases to be integrated in cBioPortal raises the question of integration into the PDF report. In principle, all data sources could always be included in the report automatically or manual selections could be made.

In addition, it may be necessary to sort the entries manually and thus name the most important information first.

	Description	Notes
manually	When creating the report, the desired annotation databases / population databases are individually selected for each mutation. However, the content of the actual data is not further selected, so that it is only decided if, for example, oncoKB's data set for a particular mutation is included in the report at all, but not if only parts of it are included.	

automatically	For each mutation contained in the report, all existing data records of all integrated annotation databases / population databases are included.	
Feature to sort entries	The relevant mutations and their annotations can be sorted manually according to the user's own rules. For example, it would be possible to name first all entries of oncoKB and then those of gnomAD for mutation A, but for mutation B first the entries of gnomAD and then those of oncoKB.	

Contents of PDF report

	**Description**	**Notes**
only annotations	The report shall only contain the relevant mutations and their annotations.	
include therapy recommendation	The therapy recommendation itself and the corresponding annotations should be included in the report.	

12. Presentation in MTB

Often preparing a case for the MTB meeting takes a lot of time. Savings potential could offer a function to support this preparation.

	**Description**	**Notes**

no feature for presentation at all	There should be no interface dedicated to the presentation for cBioPortal. The presentation is then held on the basis of the normal functions and visualizations.	
generation of slides	Function for generating slides with content to be selected in cBioPortal. Thus, all clinical data of the patient could be presented on slide 1 and on the following slides the demanded visualizations (e.g. TMB, etc.) could be presented.	
hiding individual contents in cBioPortal	For the MTB presentation the individual tabs of cBioPortal are reduced in content. Here it is still necessary to clarify which functions / contents are essential for a presentation in the MTB and which are negligible in order to keep the conciseness.	

13. General requirements

This topic deals with general requirements, more precisely with a comprehensive user and rights system as well as the connection of cBioPortal to external databases (e.g. OncoKB) and the local HIS.

Connection to external databases

cBioPortal uses various data sources such as annotation databases. It must be clarified in which way these are accessed. The following three ways are conceivable.

	**Description**	**Notes**
online	cBioPortal directly accesses the data sources of the different service providers (e.g. OncoKB or gnomAD) and therefore always processes the most up-to-date data.	

offline	cBioPortal exclusively uses copies of the data sources stored locally at the individual sites, which have to be cured in-house.	
combination of both	cBioPortal directly accesses the data sources of the different service providers (e.g. OncoKB or gnomAD) and therefore processes the most up-to-date data. In the event that a service is unavailable, the locally stored copies of the data sources are used.	

Integration into local HIS

It is not yet clarified how cBioPortal can/should be integrated into the already used existing IT structures (e.g. hospital information system with clinical workstation system, tumor documentation system, etc.) of the individual sites.

	**Description**	**Notes**
Hyperlink in system A	The corresponding hyperlink to the patient's cBioPortal data record is stored in system A (e.g. clinical workstation system) and the entry can therefore be opened up directly. At the same time, this means that no search for the corresponding patient in cBioPortal is necessary.	
cBioPortal exports data to system B	The therapy recommendation documented in cBioPortal for the treatment of a patient is exported and thus made accessible to system B.	
System B imports data from cBioPortal	System B imports the therapy recommendation stored in cBioPortal via a standardized interface (API) provided by cBioPortal.	

Data protection

It has to be clarified which data clearly identifying the patient (e.g. full name, date of birth, sex, etc.) should be stored in cBioPortal or if only pseudonymised data - in cBioPortal - may be used.

	**Description**	**Notes**
Pseudonymisation by means of a patient ID	In cBioPortal only a patient ID should be kept, which allows pseudonymization. The identifying data of the patient are stored in the corresponding system A (see "Integration into local HIS").	
Identifying data in cBioPortal	In cBioPortal - in addition to the patient ID - identifying data shall be stored for the patient. Parameters such as full name, date of birth, sex and many more could be conceivable.	

User and rights management

cBioPortal currently offers only limited functionality regarding user and rights management. In order to comply with legal requirements, it may be necessary to extend this scope.

	**Description**	**Notes**
Authentication and authorization	Only authorized users can access the data in cBioPortal.	

Traceability (integrity and authenticity)	It should be traceable at any time who changed which data record and when and how.<br>Example:<br>It is comprehensible that after the import of a patient's data by user A on 01.01.2018 at 10:00 a.m., user B changed it on 02.01.2018 at 1:00 p.m. from value X to value Y and user C completely deleted it on 03.01.2018 at 6:00 p.m.	
Limited access to certain features	Only privileged users should be allowed to use certain functions.<br>Example:<br>User A may only view the mutation table, while user B may also change the pathogenicity status of a mutation in this table.	
