# Supplementary material for: Requirements Analysis and Specification for a Molecular Tumor Board Platform Based on cBioPortal
Source: Diagnostics (Basel). 2020 Feb 10;10(2):93. doi: 10.3390/diagnostics10020093 (PMC7167859; doi:10.3390/diagnostics10020093)
Supplement: Supplementary file 1 [file diagnostics-10-00093-s001.zip › Supplementary File 6_Ethical approval.pdf]

FRIEDRICH-ALEXANDER
UNIVERSITÄT
ERLANGEN-NÜRNBERG
MEDIZINISCHE FAKULTÄT

Ethik-Kommission der FAU • Krankenhausstr. 12 • 91054 Erlangen

Jan Christoph, M. Sc.
Friedrich Alexander Universität
Lehrstuhl für Medizinische Informatik
Am Wetterkreuz 13
91058 Erlangen

Ethik-Kommission

Vorsitzender:
Prof. Dr. med. Renke Maas
Stellv. Vorsitzende:
Prof. Dr. med. Kerstin Amann

Geschäftsstelle:
Krankenhausstr. 12, 91054 Erlangen
Telefon: +49 9131 85-22270
+49 9131 85-26210
Fax: +49 9131 85-26021
E-Mail: ethikkommission@fau.de
Internet: www.ethikkommission.fau.de

Erlangen, 18.02.2019/VK

Antrag Nr.: **34_19 B** (bitte bei Schriftwechsel angeben)

Beratung nach § 15 Berufsordnung für Ärzte in Bayern

Studientitel:	Stakeholder analysis and requirement specification for a Molecular Tumorboard platform
Antragsteller:	Jan Christoph, M. Sc., Friedrich Alexander Universität, Lehrstuhl für Medizinische Informatik, Am Wetterkreuz 13, 91058 Erlangen

Sehr geehrter Herr Christoph, M. Sc.,

die Ethik-Kommission hat Ihren Antrag vom 29.01.2019 auf der Basis der Unterlagen in Anhang 1 geprüft.

Die Ethik-Kommission erhebt keine Einwände gegen die Durchführung der Studie.

Allgemeine Hinweise:
Auch bei einer positiven Beurteilung des Vorhabens durch die Ethik-Kommission der Medizinischen Fakultät der Friedrich-Alexander-Universität Erlangen-Nürnberg verbleibt die ärztliche und juristische Verantwortung für die Durchführung des Projekts uneingeschränkt bei Ihnen und Ihren Mitarbeitern/innen. Sollten sich zu diesem Projekt ethisch relevante Nachträge ergeben, bitte ich Sie, diese der Ethik-Kommission unverzüglich zusammen mit einer Bewertung der Nutzen-Risiko-Relation bekannt zu geben. Änderungen in den Dokumenten sind zum Zweck einer beschleunigten Bearbeitung deutlich zu kennzeichnen. Das Ende der Studie ist anzuzeigen und das Ergebnis zu gegebener Zeit in angemessener Form der Ethik-Kommission mitzuteilen.

Die Gültigkeit des Votums der Ethik-Kommission ist an die im Antrag angegebene Laufzeit der Studie gebunden. Wird keine Laufzeit angegeben, ist die Gültigkeit des Votums auf fünf Jahre begrenzt.

Mit freundlichen Grüßen

Prof. Dr. med. Renke Maas
Vorsitzender der Ethik-Kommission

Anhang 1
Antrag vom 29.01.2019
Anschreiben Ethikkommission.pdf
Berufsrechtlicher Antrag - 29.01.2019.pdf
Datenschutzrechtliche Einwilligungserklaerung.pdf
Einwilligungserklaerung.pdf
Probandeninformation.pdf
Studienprotokoll.pdf
curriculumVitae_2018-08-26_Dissertation_Jan-Christoph.pdf
Lebenslauf Philipp Buechner.pdf
2019-01-28_Zustimmung-Antrag-Ehtikkommission-cBioPortal-Stakeholderanalyse_Prof-Prokosch_
UNTERSCHRIEBEN-SCAN.pdf
Fragebogen_Themenpriorisierung.pdf
Fragebogen_Themenpriorisierung_Einladung.pdf
Interview_1_cBioPortal_Alternativen.pdf
Interview_1_cBioPortal_Vorstellung.pdf
Interview_1_Einladung.pdf
Interview_2_Einladung.pdf
Interview_2_Fragebogen.pdf

Anhang 2 - an der Beratung beteiligte EK-Mitglieder
Prof. Dr. Kerstin Amann, Frauenbeauftragte der Medizinischen Fakultät, Fachgebiet Pathologie, Leiterin der Nephropathologischen Abteilung
VR-OLG Dietmar Klieber, Vorsitzender Richter am OLG Nürnberg a.D.
Prof. Dr. Dieter Harms, Fachgebiet Kinderheilkunde, ehem. Leitender Oberarzt an der Kinder- und Jugendklinik
Prof. Dr. Renke Maas, Vorsitzender der Ethik-Kommission, Fachgebiet Klinische Pharmakologie und Klinische Toxikologie, Institut für Experimentelle und Klinische Pharmakologie und Toxikologie
Prof. Dr. Hajo Hamer, Fachgebiet Neurologie, Leiter des Epilepsiezentrums Erlangen
Prof. Dr. med. Robert Grützmann, Fachgebiet Allgemein- und Viszeralchirurgie, Direktor der Chirurgischen Klinik
