# Supplementary material for: Requirements Analysis and Specification for a Molecular Tumor Board Platform Based on cBioPortal
Source: Diagnostics (Basel). 2020 Feb 10;10(2):93. doi: 10.3390/diagnostics10020093 (PMC7167859; doi:10.3390/diagnostics10020093)
Supplement: Supplementary file 1 [file diagnostics-10-00093-s001.zip › Supplementary File 7_Requirements surveyed during first round of interviews.pdf]

Supplementary File 7: Requirements surveyed during first round of interviews.

We conducted the interviews in German. This is a translation of the unabridged requirements and their potential options we identified during the first round of interviews.

Background colors:

- blue: group of related requirements
- green / yellow: no further meaning; visual separation of requirements associated with same group of requirements

Requirement		Description of requirement
**General requirements**		Non-functional requirements
	**Access to external sources (e.g. knowledgebases)**	(description is provided by individual options)
	• Option 1: online	External knowledge data is loaded live from the corresponding external services when the page in question is viewed in cBioPortal. Data protection regulations must be fulfilled.
	• Option 2: offline / locally hosted	External knowledge data is cached and hosted locally in the clinical environment. Data should be updated on a regular basis.
	**Import file format**	What file format should be used for import of annotated data into cBioPortal?
	• Option 1: FASTq	Import data into cBioPortal using FASTq file format.

Requirement		Description of requirement
	• Option 2: BCL	Import data into cBioPortal using BCL file format.
	• Option 3: vcf	Import data into cBioPortal using vcf file format.
	• Option 4: annotated text file	Import data into cBioPortal using (annotated) text file format.
	**User and rights management**	Improved user and rights management in cBioPortal. It should be traceable at any time who made which changes and when.
**Documentation of therapy recommendation**		If the MTB gives a therapy recommendation, it should be possible to submit it to cBioPortal.
	**Type of data collection**	(description is provided by individual options)
	• Option 1: free text	Completely free text recording of the data similar to the writing of a doctor's letter.
	• Option 2: structured	Completely structured collection of data. This could also be advantageous for later automatic evaluations.
	**Integration into hospital information system**	(description is provided by individual options)
	• Option 1: cBioPortal pushes data to HIS	cBioPortal pushes data to HIS. Problem: Hospitals use different systems, so access to the appropriate interface has to be adapted for each location.
	• Option 2: HIS pulls data from cBioPortal	cBioPortal provides an API and therefore the HIS can pull the data on a regulary basis.

Requirement		Description of requirement
	**Access to therapy recommendation**	(description is provided by individual options)
	• Option 1: Access allowed for partner sites	All MIRACUM partner sites can access the therapy recommendations of each site.
	• Option 2: Access only allowed for local site	The therapy recommendation can be accessed only by local staff.
	**Content of therapy recommendation**	(description is provided by individual options)
	• Option 1: Follow-up data	Providing follow-up data about a patient. Was a therapy recommendation actually followed and what was the outcome of this decision?
	• Option 2: Clinical data at the time of registration for the MTB	In order to be able to place the therapy recommendation into the correct context, it is also necessary to present relevant information such as patient basic data (age, gender, current performance status (ECOG), etc.), tumour identity, tumour spread, previous therapies/therapy progressions, for each therapy: success of the therapy, i.e. response: PR/CR/Progress, etc. Some of these data are already specified in the patient registration for the MTB and could be integrated into the therapy recommendation field.
	• Option 3: Experimental	Mark a recommendation as "experimental therapy". This could also be achieved by providing an evidence level.
	• Option 4: Participation in study	Is participation in a study necessary for the therapy?
	• Option 5: References in literature	Reference to literature to justify the therapy recommendation.

Requirement		Description of requirement
	**Selection of mutations as being "relevant for therapy recommendation"**	Marking or classification of the mutation(s) that are considered relevant for the therapy decision or on the basis of which the therapy recommendation is made. This allows the therapy recommendation to be linked transparently to the mutation data and used for later retrospective evaluations of therapy recommendations/results (e.g. by searching for a marked dominant mutation or mutation combinations for which all therapy decisions are then displayed).
	• Option 1: Checkbox for selection of multiple mutations	Simple (dichotomous) decision as to whether a mutation is relevant or not. No further annotations, classifications or pathogenicity classifications.
	• Option 2: Checkbox for selection of multiple mutations with optional free text field	Dichotomous decision as to whether a mutation is relevant or not. Free text field for further annotations to the mutations selected as relevant.
	• Option 3: Checkbox (radio button) for selection of a single mutation	Selection of only a single mutation; multiple selection is not allowed.
	• Option 4: Pathogenicity classification	Automatically mark mutations as relevant for therapy recommendation based on pathogenicity classification (see below “Pathogenicity of mutations”).
	• Option 5: Classification "predictive", "diagnostic", "confirmatory"	Classification of each mutation with the properties "predictive", "diagnostic" and "confirmatory"; multiple selection explicitly permitted.
	**Search function for similar patients**	Search function to manually search for "comparable" patients (the respective definition of comparability is incumbent on the subjective definition of the respective seeker / acting physician).
	• Option 1: Based on a single mutation	Search for patients with a single, specific mutation.

Requirement		Description of requirement
	• Option 2: Based on a mutation pattern	Search for patients with a specific pattern of mutations.
	• Option 2a: Coverage ratio = 100%	Search for patients whose mutations match a mutation pattern to 100%.
	• Option 2b: Coverage ratio <= 100%	Search for patients whose mutations match a mutation pattern to 100%. or less (e.g. at least 70%; ratio should be parameterizable)
	• Option 2: based on gene a mutation relates to	Search for patients with a specific mutated gene
	• Option 3: based on tumour entity	Search for patients based on a specific tumour entity
	**Generation of text for therapy recommendation**	If a therapy recommendation is recorded in a structured way, it should be possible to automatically generate a continuous text (similar to a doctor's letter) on it.
	**Selection of mutations as justification for recommendation**	For each component of the recommended therapy a set of one or more (previously selected: see T2-A5) mutation(s) should be selectable as justification for the recommendation.
**Features to support decision making regarding a therapy recommendation**		(description is provided by individual options)
	**Pathway analysis**	Feature to analyse the pathways affected by a specific mutation in order to identify possible targets for therapy besides the actual mutated gene.

Requirement		Description of requirement
	• Option 1: Pathway-Map	Visualization of the pathways affected by a specific mutation by a map. Possible databases may be GeneOntology, STRING-DB, GSEA Broad Institute, "Consensus Path DB", "Reactome", MSG-DB, "Msig DB, Hallmarks of Cancer" etc.
	• Option 2: color marking of mutations in the same pathway	Marking of all mutations affecting a specific pathway by color.
	**Tumor Mutational Burden (TMB)**	(description is provided by individual options)
	• Option 1: displaying the value in the context of the corresponding tumor entity	The TMB may be placed in the context of the corresponding tumor entity the patients suffers from.
	• Option 2: displaying the value in the context of the corresponding tumor entity and other possible entities	The TMB may be placed in the context of the corresponding tumor entity the patients suffers from. If the TMB seems to be atypical, other suitable tumor entities should be suggested.
**Mutation occurences across MIRACUM partner sites**		Feature to gain an overview of how often a mutation has already been registered at MIRACUM partner sites and/or in international institutions. If necessary, contact may be made with these institutions and, if necessary, their therapy recommendations (including feedback on therapy success) may be requested, discussed and taken into account for one's own therapy recommendation.
	**Beacon Network**	Integration of services like "Beacon Network" (https://beacon-network.org/) to see how often a specific mutation was identified at international level. This may help to identify artifacts generated by the sequencer.

**Requirement**		**Description of requirement**
	**MIRACUM occurrences**	Display how often a particular mutation has already been identified at the local hospital and/or in the entire MIRACUM network. In comparison to the Beacon network, the focus here is on the MIRACUM consortium and a option to contact partner sites that based an earlier therapy recommendation on this mutation.
	• Option 1: display of occurrences including contact data	Besides displaying the occurrences of a specific mutation contact data to request the therapy recommendation from the partner site should be provided.
	• Option 2: display of occurrences including therapy recommendation of remote partner site	Besides displaying the occurrences of a specific mutation, the therapy recommendation given by the corresponding MTB of the partner site should be provided.
	• Option 3: display of occurrences by tumor entity	Subdivide occurrences by tumor entity. Example: "Site A: entity a: 2x, entity b: 1x; Site B: entity a: 1x, entity c: 2x".
	• Option 4: display of occurrences regardless of therapy relevance	The occurrence of a specific mutation should be displayed regardless if its existence also contributed to the therapy recommendation.
	• Option 5: display of occurrences only if mutation was relevant for therapy	The occurrence of a specific mutation should only be displayed if its existence also contributed to the therapy recommendation.
	• Option 6: integration of occurrences in mutation table	Integrate the display of occurrences into mutation table in patient view (e.g. via popup dialog).
	• Option 7: search for occurrences independently	Feature to search for occurrences independently of a patient case (e.g. to answer research questions).

Requirement		Description of requirement
	**Forum for discussions**	Development and establishment of a forum to discuss individual mutations. This allows each site to comment on a mutation and to exchange experiences. For each mutation in the mutation table in cBioPortal there should be a hyperlink to the corresponding thread in the forum.
**Pathogenicity of mutations**		Klassifikation einer Mutation (über Drop-Down je Mutation), um dessen Relevanz für die Therapieentscheidung feststellen/festlegen zu können. Je nach Klassifizierung würde diese Mutation dann z.B. mit in einen automatischen Report übernommen werden.
	**Classification A-D for each mutation**	A: highly pathogenic; B: potentially pathogenic; C: unknown significance; D: likely benign
	• Option 1: manual classification	Feature to classify each mutation manually by the user
	• Option 2: semi-automatic classification	Feature to automatically classify each mutation and possibility to edit the classification afterwards
	• Option 3: automatic classification	Feature to automatically classify each mutation without the possivility to edit the classification afterwards
	**Classification 1-5 for each mutation**	5: pathogenic; 4: likely pathogenic; 3: uncertain significance; 2: likely benign; 1: benign
	• Option 1: manual classification	Feature to classify each mutation manually by the user
	• Option 2: semi-automatic classification	Feature to automatically classify each mutation and possibility to edit the classification afterwards
	• Option 3: automatic classification	Feature to automatically classify each mutation without the possibility to edit the classification afterwards

Requirement		Description of requirement
	**Filter for mutation table**	Feature to apply filters to mutation table right in cBioPortal (instead of / additional to MIRACUM-Pipe)
	• Option 1: display filter status	Indicate whether filters have been applied or not and if so, indicate which user applied the filter.
	• Option 2: custom (predefinable) filter-sets	Users should be able to create filter-sets and reuse them in other patient cases. For example, a filter may limit the displayed mutations to those that exceed a certain coverage and other criteria. This filter settings may be saved in cBioPortal with a description (e.g. "Filter XY") and reused in subsequent analysis of samples from the same or even other patients.
	• Option 3: persistent filter	Filters should be applicable temporarily and persistently to a sample's mutations.
	• Option 4: list only relevant mutations	cBioPortal should only list relevant mutations. In this context, the meaning of relevance has not been defined more precisely (e.g. list only mutations that are (likely) pathogenic).
	• Option 5: list all mutations	cBioPortal should always list all called mutations. This means, cBioPortal does not apply any filters (this is done by MIRACUM-Pipe prior import to cBioPortal).
**Annotations**		(description is provided by individual options)
	**MyCancerGenome**	Integration of MyCancerGenome (https://www.mycancergenome.org/)
	**CIViC**	Integration of CIViC (https://civicdb.org/) or implementation of an alternative database
	• Option 1: data cured by MIRACUM partners	MIRACUM (and this Use Case 3) should establish its own database, which resembles the CIViC database and may only be cured by consortium members.

Requirement		Description of requirement
	• Option 2: link to publicly available data	For each mutation in the mutation table in cBioPortal a hyperlink to the corresponding CIViC entry should be provided.
	**JAX-CKB**	Integration of JAX-CKB (https://ckb.jax.org/)
	**Minor Allele Frequencies**	(description is provided by individual options)
	• Option 1: ClinVar	Integration of ClinVar (https://www.ncbi.nlm.nih.gov/clinvar/)
	• Option 2: dbSNP	Integration of dbSNP (https://www.ncbi.nlm.nih.gov/projects/SNP/)
	• Option 3: ExAC / gnomAD (neuere Version von ExAC)	Integration of ExAC / gnomAD (http://exac.broadinstitute.org/ / https://gnomad.broadinstitute.org/)
	**Quality scores**	Integration of quality scores
	• Option 1: phred quality scores	Display of phred quality scores to quantify accuracy of base calling by the sequencer
	• Option 2: variant allele frequency	Display of VAF for each mutation
	• Option 3: coverage	Display of coverage for each mutation
	**Cohort representation**	Currently the frequency of a mutation in a cohort is displayed in cBioPortal. This cohort is formed from all samples assigned to a study. It has to be clarified whether other cohorts have to be formed, too.

Requirement		Description of requirement
	• Option 1: cohort by entity	Cohort: All samples from all patients who were also assigned to the current patient's tumor entity
	• Option 2: local cohort	Cohort: All samples from all patients at the local hospital
	• Option 3: global cohort	Cohort: Samples from multiple (not yet specified) international / global studies
**CNV (Copy Number Variation)**		(description is provided by individual options)
	**Visualization**	(description is provided by individual options)
	• Option 1: graphical; like in SOPHiA GENETICS	Visualization of CNV data like in SOPHiA GENETICS (https://www.sophiagenetics.com/home.html).
	• Option 2: graphical; not like in SOPHiA GENETICS	Not described in detail.
	• Option 3: simple tabular	Tabular visualization like in cBioPortal.
	**Grouping**	(description is provided by individual options)
	• Option 1: not grouped / per gene	Display CNV data ungrouped / per gene / like in cBioPortal.
	• Option 2: per chromosome	Group CNV data by chromosome and display total amplifications / deletions.
	• Option 3: per genome	Group CNV data by genome and display total amplifications / deletions.

Requirement		Description of requirement
**Visualization of methylation**		Feature to visualize methylation data.
**Meta data about sample**		(description is provided by individual options)
	• Option 1: highlight germline mutations	Explicitly highlight (e.g. by placing a symbole) germline mutations in mutation table.
	• Option 2: display type of sampling	E.g. fresh-frozen or formalin-fixed paraffin-embedded samples
**Mark mutations with therapy options**		Mark mutations in mutation table based on a whitelist with mutations for which targeted therapy is available.
**Display expression of mutated gene**		For each mutation display status of expression based on RNA-Seq data.
**Generation of PDF report**		cBioPortal should provide a revision-proof PDF report with relevant data from all annotation databases that led to a therapy recommendation. This report may then be passed to the MTB's client (e.g. the treating physician) and presents the therapy recommendation to them in a transparent way.
	**Include only relevant mutations**	Only relevant mutations should be included in the report.
	• Option 1: manual selection	The relevant mutations should be selected manually by the user.
	• Option 2: automatic selection	The relevant mutations should be selected automatically by cBioPortal, e.g. based on pathogenicity classification.
	**Include data from relevant annotation databases**	(description is provided by individual options)

**Requirement**		Description of requirement
	• Option 1: manual selection	The databases to be included in the report is specified manually by the user. For example, for mutation A data from databases X and Y should be included and for mutation B only data from X.
	• Option 2: automatic selection	For each mutation in the report data from all databases should be included automatically by cBioPortal.
	**Information covered by report**	(description is provided by individual options)
	• Option 1: just data from annotation databases	The report should only include the (relevant) mutations and corresponding data from annotation databases. The report should not include the therapy recommendation itself.
	• Option 2: data from annotation databases and therapy recommendation	The report should include the (relevant) mutations and corresponding data from annotation databases. The report should also include the therapy recommendation itself.
	**Feature to sort occurrences of individual mutations in report**	For each mutation included in the report, the occurrence of data from different annotation databases in the report should be sortable by the user.
**Details about sequencing**		cBioPortal should provide information of sequencing (WGS vs .WES vs. panel, name and version of panel used).
**Feature to support presentation in MTB meeting**		Feature to support case presentation in the meeting of the MTB by generating slides with relevant data.
**Clinical data about patient**		Integration of clinical data in cBioPortal (e.g. age, sex, tumor entity, TNM staging, topographic and morphological classification of the tumor entity by the International Classification of Diseases for Oncology)
	**Integration in cBioPortal**	General decision if clinical data should be integrated into cBioPortal (or stored exclusively in another part of the HIS).

Requirement		Description of requirement
	• Option 1: no usage in cBioPortal	cBioPortal should not contain any clinical data.
	• Option 2: usage in cBioPortal	cBioPortal should contain clinical data which may be redundant to those stored in the HIS.
**Search for suitable studies**		Since some drugs are available only in preclinical studies, a feature to easily search for suitable clinical trials is necessary (e.g. https://clinicaltrials.gov). This may also help to find arguments for reimbursement by health insurance companies for the off-label use of a drug.
	**Source**	https://clinicaltrials.gov could serve as a source for this search functionality.
	**Radius**	(Geographical) radius to be covered by the search.
	• Option 1: Germany only	Only list studies taking place in Germany.
	• Option 2: worldwide	List all studies worldwide.
	**Search parameters**	(description is provided by individual options)
	• Option 1: drug(s)	User should be able to include certain drugs in the search query.
	• Option 2: mutation(s)	User should be able to include certain mutations in the search query.
	• Option 3: tumor entity	User should be able to include the patient's tumor entity in the search query.
**General requirements OncoKB**		Non-functional requirements (OncoKB)

Requirement		Description of requirement
	**Drug recommendation basis**	(description is provided by individual options)
	• Option 1: mutation	For each mutation in the patient's sample recommended and suitable drugs should be listed (like it is already implemented in cBioPortal).
	• Option 2: tumor entity	All drugs explicitly approved for this tumor entity should be listed. This should be independent of whether or not a corresponding mutation (target of the drug) could be identified in the patient's sample.
**Approval status**		Improvement of OncoKB's drug recommendation by adding details about approval status.
	**Requirements to the display itself**	(description is provided by individual options)
	• Option 1: display without details about approved tumor entities	Display approval status of a recommended drug without details about the explicitly approved tumor entities (e.g. "Drug A was approved").
	• Option 2: display with details about approved tumor entities	Display approval status of a recommended drug with details about the explicitly approved tumor entities (e.g. "Drug A was approved for AML and biliary tract cancer").
	• Option 3: display regardless of level of evidence	Display approval status of a recommended drug regardless of its level of evidence.
	• Option 4: display only if certain level of evidence is exceeded	Display approval status of a recommended drug only if a certain level of evidence is exceeded.
	**Approving authority**	Display approving authority (e.g. "Durg A was approved by EMA / FDA / ...").

Requirement		Description of requirement
**Mutations causing resistance**		(description is provided by individual options)
	• Option 1: display in OncoKB popup	Display a potential resistance next to the corresponding drug recommendation in OncoKB popup in cBioPortal.
	• Option 2: display symbol indicating existing resistance in mutation table next to corresponding mutation	For each mutation in mutation table display a symbol if it is causing a resistance against a drug.
	• Option 3: display symbol with hyperlink to details	For each mutation in mutation table display a symbol if it is causing a resistance against a drug. This symbol should serve as a hyperlink to an overview of all mutations causing resistance against this specific drug.
**Availability of a certain drug in Germany**		(description is provided by individual options)
	**Level of detail**	(description is provided by individual options)
	• Option 1: simple yes / no	Indicate that a certain drug is available in Germany by simply displaying "yes" or "no".
	• Option 2: name of preparation and manufacturer	Indicate that a certain drug is available in Germany with details about the name of the preparation and the manufacturer.
