# Supplementary material for: Requirements Analysis and Specification for a Molecular Tumor Board Platform Based on cBioPortal
Source: Diagnostics (Basel). 2020 Feb 10;10(2):93. doi: 10.3390/diagnostics10020093 (PMC7167859; doi:10.3390/diagnostics10020093)
Supplement: Supplementary file 1 [file diagnostics-10-00093-s001.zip › Supplementary File 9_Details about all consolidated requirements.pdf]

Supplementary File 9: Details about all consolidated requirements.

Note: For all screenshot mockups see Supplementary File 8.

1 Clinical data

In general, it is already possible to store clinical data of a patient in cBioPortal. However, the MIRACUM consortium has not yet reached an agreement on a unified data set, which is why we specifically asked for the attributes that the participants considered to be relevant during both interview phases. In addition to the more general data such as the age and sex of the patient, descriptive characteristics and classifications of diseases were also mentioned. These include TNM staging or the topographic and morphological classification of the tumor entity by the International Classification of Diseases for Oncology (ICD-O).

For further attributes, interviewees referenced to an announcement published by the German Federal Ministry of Health, which, among others, integrates further attributes for the description of histological findings or already conducted therapy attempts [1].

The timestamp of these records is considered to be an essential metadatum (e.g., time of TNM classification) and is also necessary for visualizing them in the already implemented timeline feature of cBioPortal. According to the interviewees, this possibility for a time-based recapitulation is an important instrument to obtain an overview of the current case.

Further, a common tumor entity ontology should be established upon all MIRACUM sites. In addition to the ICD encodings mentioned above, which are currently used in most of our partner sites, it is also an option to name the entities according to OncoTree [2] which was unknown to most of the interviewees. The sites, who treat patients in the domain of neuro-oncology, currently use, except for entities or metastases that are not located in the CNS, a specialist classification developed by the World Health Organization in 2007 [3].

2 Sample metadata

A further requirement is the display of additional metadata about sample information that has been used for analysis. The required data are:

- localization and time of the sampling
- type of sampling (e.g., fine-needle aspiration biopsy)
- distinction between fresh-frozen and formalin-fixed paraffin-embedded samples

Furthermore, details of the sequencing itself should be available. The following metadata was mentioned for this purpose:

- scope of sequencing (e.g., gene panel or whole-exome sequencing)
- name and version of both the used panel and kit
- hyperlink to the corresponding product-specific website of the manufacturer

All these metadata are essential not only for the evaluation of the current patient case but also for retrospective analysis in the context of studies or the search for similar patients. If someone would like to compare two samples (e.g. from two different patients) with each other, it is necessary to know

which genes were covered by the respective panels used. If they do not cover the same genes and may only have a small or no intersection at all, the results may not be comparable. The same applies to the comparison of different samples of a single patient.

3 Scores

The MIRACUM-Pipe [4] annotates variants with multiple scores, but not all of them can be displayed by cBioPortal. The interviewees specified the visualization of them as a requirement, whereas the Condel score was explicitly named. This score helps classifying mutations as deleterious or neutral and outperforms the individual tools, it integrates [5], including SIFT [6], PolyPhen-2 [7] and MutationAssessor [8] that are already integrated individually in cBioPortal.

Interviewees also demanded to display a phred quality score quantifying the accuracy of base calling by the sequencer [9]. All would prefer the implementation of the manufacturer of a base caller as opposed to a manufacturer-independent calculation.

The participants considered the visualization of the Copy Number Alterations (CNV) data in cBioPortal as sufficient (nine out of nine sites and no sites abstained from voting). However, as an improvement they requested to also display the exact count of copies of the amplicon. Two sites also requested to display the total count of Copy Number Alterations per chromosome (the remaining seven sites did not and no sites abstained from voting).

The Tumor Mutational Burden seemed to be of special interest in respect of therapy options. The interviewees required the display of the corresponding value for each sample. Ideally, this score should be placed in the context of the tumor entity and graphically displayed using a boxplot (see Figure A1). The TMB is particularly relevant when immunotherapy of certain tumor entities is a therapeutic option [10–13] and it is "(…) also most likely to be adopted as predictive biomarker (…)" [14]. Eight out of nine sites voted for a boxplot representation of the TMB while only one site preferred the numeric only option (no site abstained from voting).

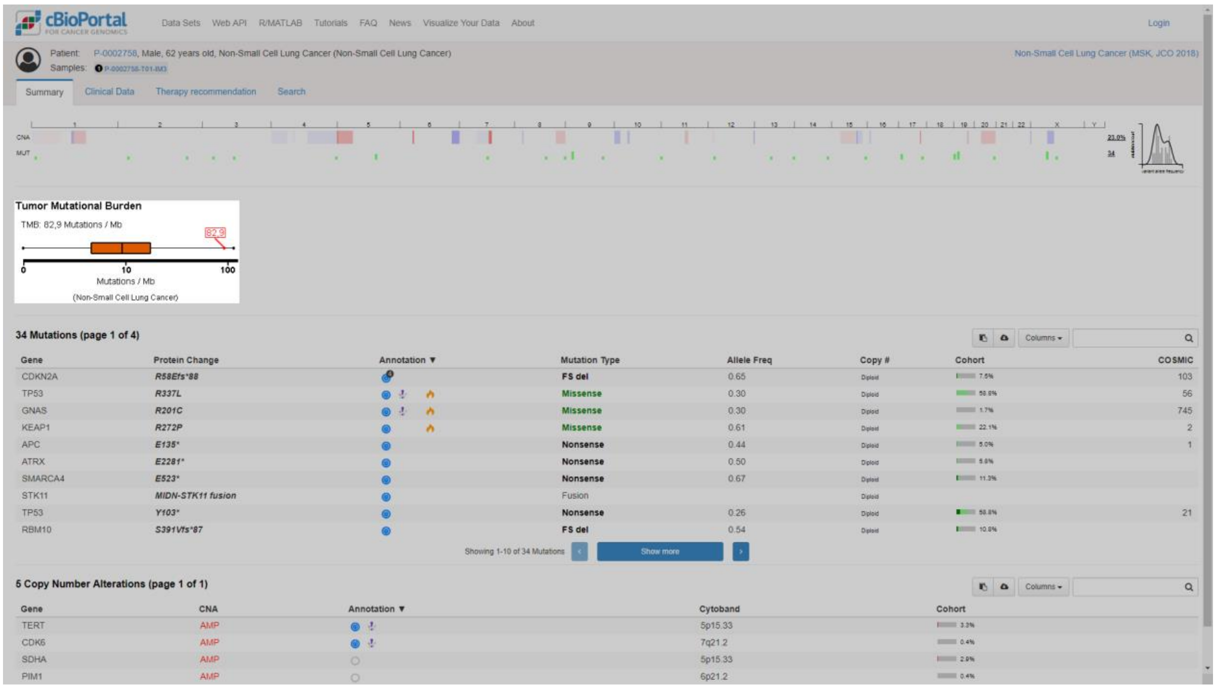

Figure A1. Screenshot mockup with visualization of Tumor Mutation Burden using a boxplot. Example data adopted from MSKCC's publicly available cBioPortal instance [15].

4 Integration of further annotation databases for clinical interpretation

The participants requested the integration of further annotation databases into cBioPortal, particularly, JAX-CKB [16] aroused the most interest. The data showed on the screenshot mockup (see Figure A2) was sufficient according to the sites already experienced with this database.

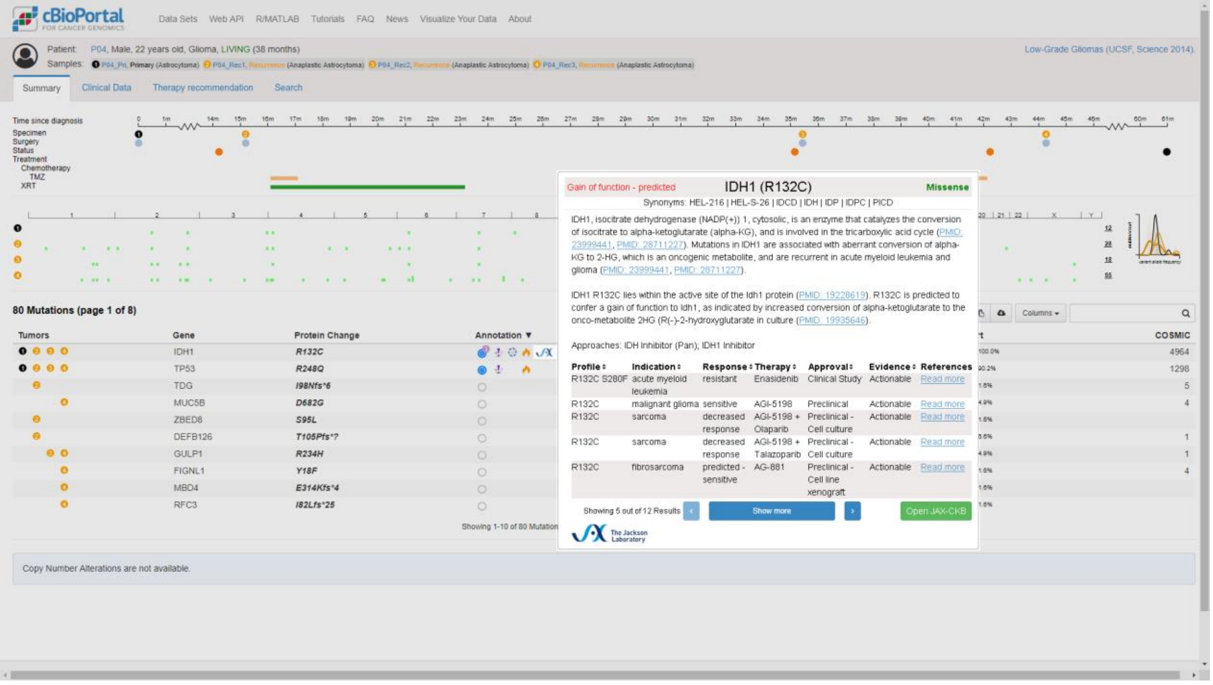

Figure A2. Screenshot mockup with visualization of data provided by JAX-CKB. Example data adopted from MSKCC's publicly available cBioPortal instance [15] and JAX-CKB knowledgebase [16].

Since many different databases have to be consolidated to develop a therapy recommendation [17], an integration of ClinVar [18] was also suggested.

The current rudimentary integration of My Cancer Genome [19], consisting only of hyperlinks belonging to a mutation, was considered acceptable by six sites and does not require any modification (two sites requested a more comprehensive integration and one site abstained from voting).

Together with the databases already integrated into cBioPortal, such as OncoKB or CIViC [20], these extensions can create an even more powerful tool, which can further improve and accelerate the preparation of an MTB by bypassing time-consuming manual searches.

5 Minor Allele Frequencies

To determine whether a mutation is rare, interviewees need to consult databases in which the frequency of mutations in a particular population is listed. In the interviews, the Genome Aggregation Database (gnomAD; see Figure A3) [21], the Exome Aggregation Consortium (ExAC) [22] and the Single Nucleotide Polymorphism Database (dbSNP) [23] were listed as the databases used at our partner sites. gnomAD and dbSNP seemed to be equally important; gnomAD's predecessor version ExAC [21] less.

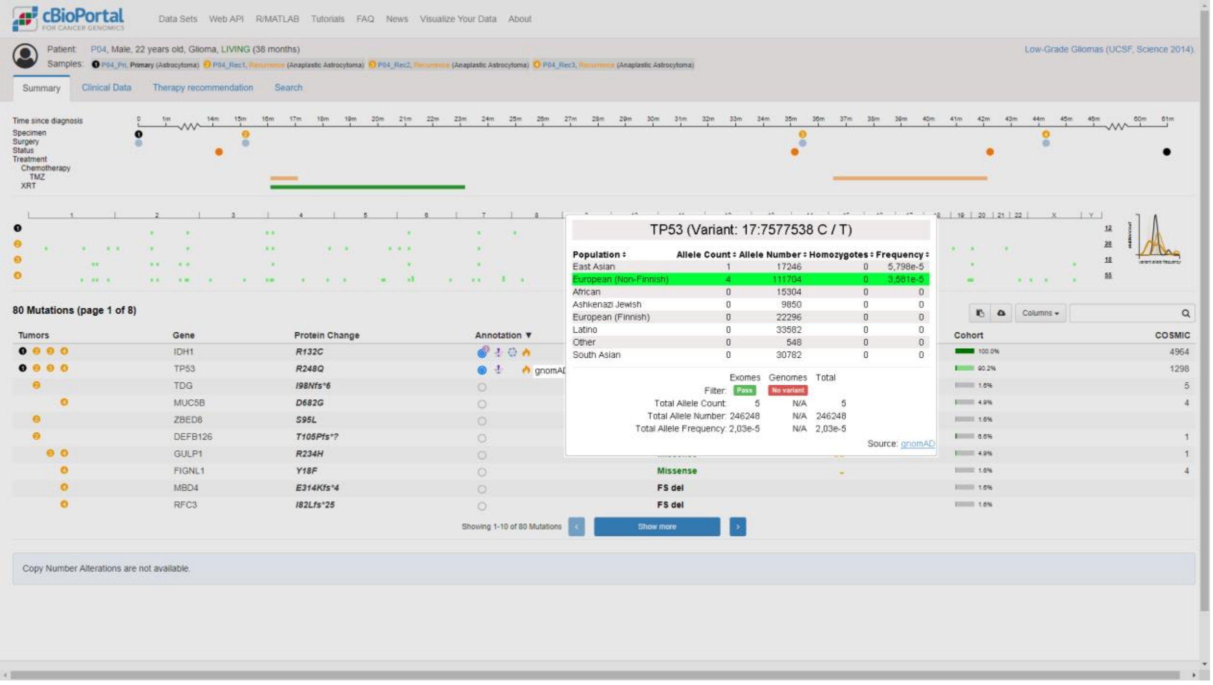

Figure A3. Screenshot mockup with visualization of data provided by gnomAD. Example data adopted from MSKCC's publicly available cBioPortal instance [15]and gnomAD [21].

It was important to the participants that for every mutation the frequencies for all populations are displayed, even if according to them in Germany the data of the population "European (non-Finnish)" are most often used. These records, for example, may be interesting and informative in pediatric oncology, if the parents of a child originate from different populations. For the interviewees, it does not make sense to display only the mean values of these remaining populations.

Starting by integrating gnomAD into cBioPortal seems to be a good idea, because reference links to dbSNP are provided in this database, too. Thus, the information of dbSNP can also be accessible at one single click.

6 Cohort representation

The participants requested the display of a mutation's frequency of occurrence in a cohort. This cohort should be a combination of the data of as many international studies as possible and be limited to the corresponding tumor entity of the patient. cBioPortal already offers a similar function which, however, is based on the corresponding (single) study cohort to which the patient was once assigned. The result is then displayed both numerically and graphically by means of a small vertical bar. This feature also indicates how often the respective gene is altered in the cohort at all.

Currently, the functionality requested above can only be achieved indirectly in cBioPortal by creating a study query that searches for the mutation in arbitrary studies - assuming those are available in the local cBioPortal instance. However, this requires additional interaction by the user and cannot be displayed directly in the mutation table of the currently viewed patient.

A possible source for the cohort data could be the studies already available within the public instance of cBioPortal, which already contains a large number of samples sorted by many different entities.

Consortium-internal or local cohorts are considered less important as the cohort size seems not to be sufficient for the foreseeable future. Same applies for entity-independent cohorts.

Nevertheless, some sites requested a local cohort to identify possible artifacts of the panel used. This cohort should contain all samples regardless of the underlying tumor entity. They report that it is possible that a (faulty) panel may always detect a particular mutation to be existent (false-positive). This mutation would then occur in every sample of the local cohort and this would be conspicuous, for example, in more rare mutations.

7 Mutation occurrences across MIRACUM partner sites

The interviewees asked about a way of improving the exchange of experience for identified mutations between the partner clinics. For this purpose, an infrastructure for reporting found mutations was proposed. Each cBioPortal instance should report a mutation finding automatically if users consider it to be relevant for the treatment of a particular tumor entity (see 13 Therapy relevant mutation). If a partner site discovers the same mutation in a later patient case, the total and entity-specific count of findings in the network should be displayed and the reporting site's contact details, such as e-mail address or telephone number, should be provided (see Figure A4). Eight sites requested a distinction of the count of findings based on the underlaying tumor entity (one site requested the display of the total count only and no sites abstained from voting).

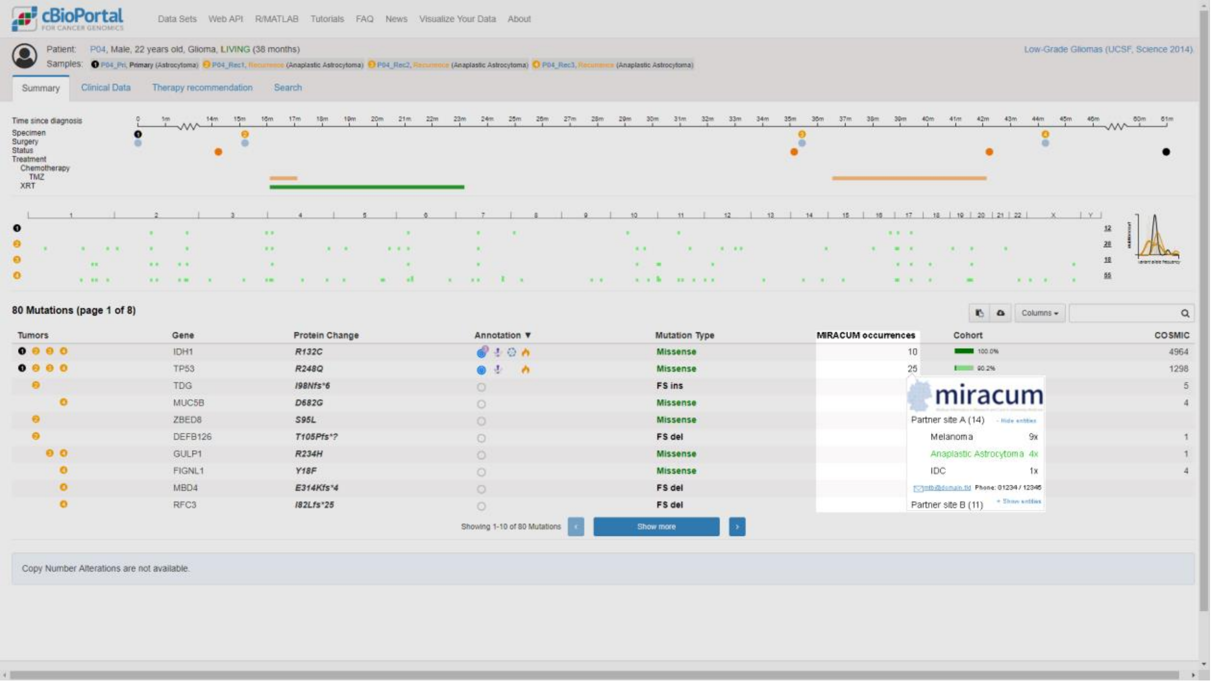

Figure A4. Screenshot mockup demonstrating a popup with information about mutations found at partner sites. Example data adopted from MSKCC's publicly available cBioPortal instance [15].

A superior goal of the German Medical Informatics Initiative, which also MIRACUM belongs to, is the networking of clinical data. For this reason, most of the sites also demanded that this network should provide access to those data so they are able to familiarize with a case before getting in contact. Five sites voted for MIRACUM-wide access of patient details while three sites only requested contact details to be displayed (one site abstained from voting).

Less often interviewees requested a possibility to also report all remaining mutations that have not been marked as relevant (see 13 Therapy relevant mutation). More specifically, this was requested by a single site only; three sites requested a distinction between relevant and not relevant mutations but demanded all mutations to be listed. The remaining five sites requested only relevant mutations to be listed (no site abstained from voting).

The integration of databases tracking international occurrences of mutations was requested by only three sites. From the remaining sites five did not see a benefit in such a feature and one site abstained from voting.

8 Pathway analysis and mapping

According to the interviewees, it may happen that a therapy recommendation is supposed to intervene apart from an existing mutation. For this, it is necessary to identify and analyze the different pathways of a mutation in order to find such a target and, for example, inhibit it. While it is still common at our partner sites with (smaller) gene panels to know the targeted genes and their interactions and pathways in detail, this seems to be impossible in terms of WES or WGS.

Therefore, cBioPortal should automatically name the corresponding pathways of each mutation and ideally also visualize them (see Figure A5). For the latter, a service such as GeneCards (https://www.genecards.org) may be used and the corresponding mutation and its pathways may refer to the corresponding site via a hyperlink. While seven sites may be satisfied by mentioning the pathways in the mutation table (see Figure A5), the two remaining sites requested comprehensive maps for visualization (no site abstained from voting).

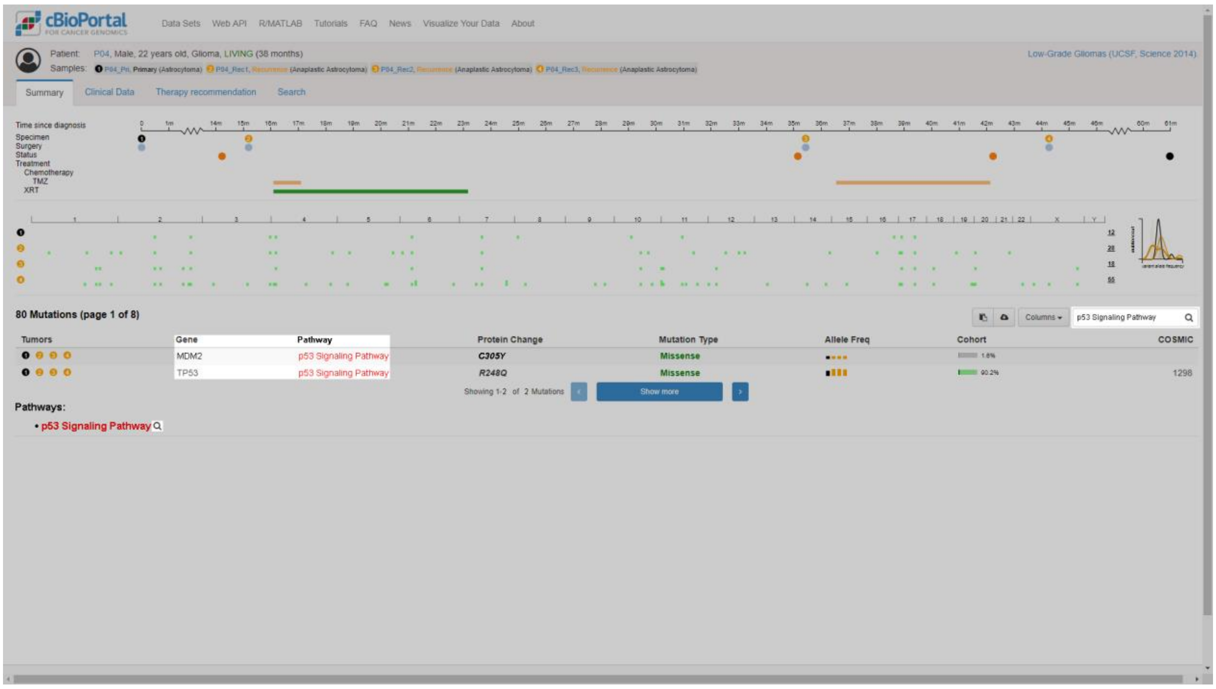

Figure A5. Screenshot mockup demonstrating visualization of pathways. Example data adopted from MSKCC's publicly available cBioPortal instance [15].

9 Drug information

As OncoKB currently only provides mutation-specific information about drug approvals by the U.S. Food and Drug Administration (FDA), corresponding information from its European equivalent, the European Medicines Agency (EMA), and ideally the German Federal Institute for Drugs and Medical Devices (BfArM) has to be added for use in Germany (see Figure A6).

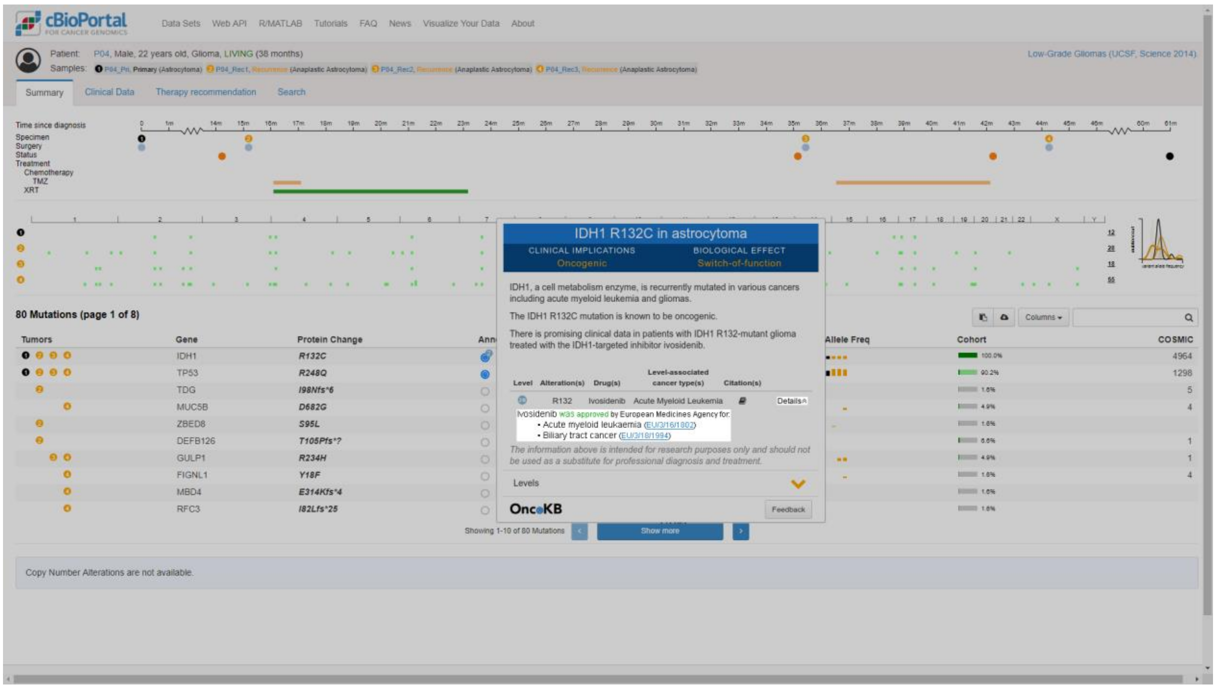

Figure A6. Screenshot mockup demonstrating visualization of approval status of a drug based on tumor entity. Example data adopted from MSKCC's publicly available cBioPortal instance [15] and OncoKB [24].

This allows to quickly check whether a certain therapy is actually approved in Germany, since this may differ between the two authorities mentioned above. But even if the number of users of the BfArM information is limited to a national level, at least the approval by the EMA is also of international, European interest [25].

In addition, two sites wanted OncoKB to only propose a drug if the therapy meets a certain (not yet determined) level of evidence, while from the remaining sites three wanted to remain independent and four abstained from voting; a clear trend is not discernible, but since OncoKB also lists the respective level of evidence the former variant can be archived by simply ignoring those drugs not complying with the level of evidence.

On the other hand, there is more agreement on the demand for the distinction of approvals by the entity, because some drugs are only approved for the treatment of certain entities or mutations (four sites requested such a distinction while two sites did not request this information and three sites abstained from voting).

In the first round of interviews, some sites demanded cBioPortal to list all approved drugs for the current patient's tumor entity independently from the mutations identified in the sample(s). In the second round of interviews five sites voted against this feature while the remaining four sites abstained from voting.

10 Visualization of methylation

So far, only some of the MIRACUM sites have included DNA methylation analysis in their daily MTB routines. Therefore, no consensus could be reached on how these data could be visualized in cBioPortal. As well as the listing of the data in a table, the visualization of the affected pathways in a heat map or a bar plot was suggested. The illustration in a network map was also mentioned as a

possibility. Ultimately, the type of presentation depends on the questions to be answered, on which no consensus has yet been reached in the consortium.

11 Parameterizable query in search engines

The sites mentioned that they use search engines on the internet in case of questions. In such a situation they search for the corresponding mutation (e.g., “G12D”) or additionally for the name of the gene (e.g., “G12D KRAS”).

In order to save time, the interviewees demanded that a hyperlink to a search engine is provided for each variant in the mutation table, which automatically adopts the search term and presents the results.

It would be advantageous to make the search at least instance-specific, but better user-specific, parameterizable. For this, the automatically generated link including the search term should be adjustable (see example above). In order to remain flexible in the choice of the search engine provider, the URL could also be adjustable using placeholders, which are then replaced by the search terms. The latter, however, was not explicitly requested, as the sites base their searches on Google only.

12 Mutation classification

The MIRACUM-Pipe [4] automatically annotates all called variants with a classification describing their pathogenicity. Therefore, the “(…) specific standard terminology: ‘pathogenic’, ‘likely pathogenic’, ‘uncertain significance’, ‘likely benign’, and ‘benign’ (…)” [26] is applied (four sites explicitly requested the display of this data in cBioPortal while the remaining five sites abstained from voting). Since this automatic assessment is based on databases that may contain incorrect data, a way to correct the decision manually was also requested (seven out of nine sites voted for this semi-automatic assessment while one site voted for a manual only assessment and one site abstained from voting). If this becomes necessary, the manual change must be clearly distinguishable from the automatic assignments, for example by marking it with an asterisk (see Figure A7).

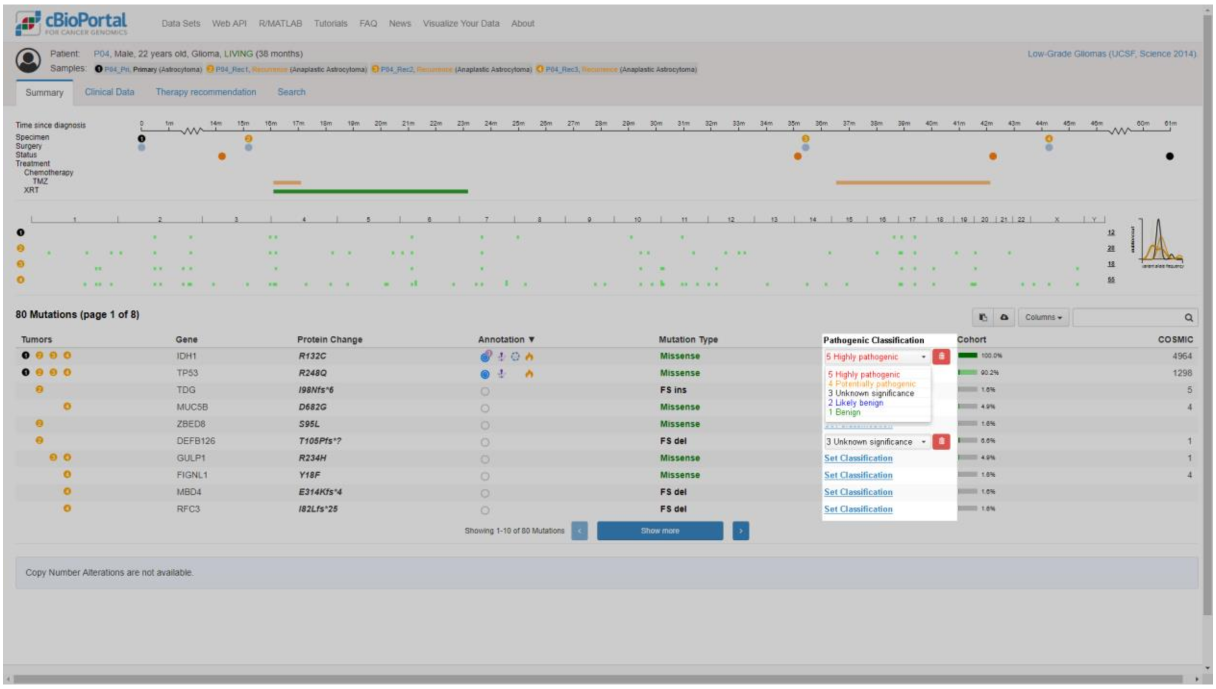

Figure A7. Screenshot mockup demonstrating visualization and editing of pathogenic classification. Example data adopted from MSKCC's publicly available cBioPortal instance [15].

13 Therapy relevant mutation

According to our partner sites, the user should be able to mark a mutation as relevant in order to document the recommendation of a targeted therapy later on (see 18 Therapy recommendation). For this purpose, the mutation table should be extended to provide a checkbox for each mutation. This also allows the selection of multiple mutations even across different genes (all nine sites demanded the selection of multiple mutations and no site abstained from voting).

Furthermore, the introduction of a small free text field for each mutation is considered to be an additional aid (see Figure A8) by six sites (the remaining three sites did not request a text field and no site abstained from voting). The purpose of this field, though, varies from site to site. Some would use it to make a short note between the diagnosticians, others to briefly justify their decision.

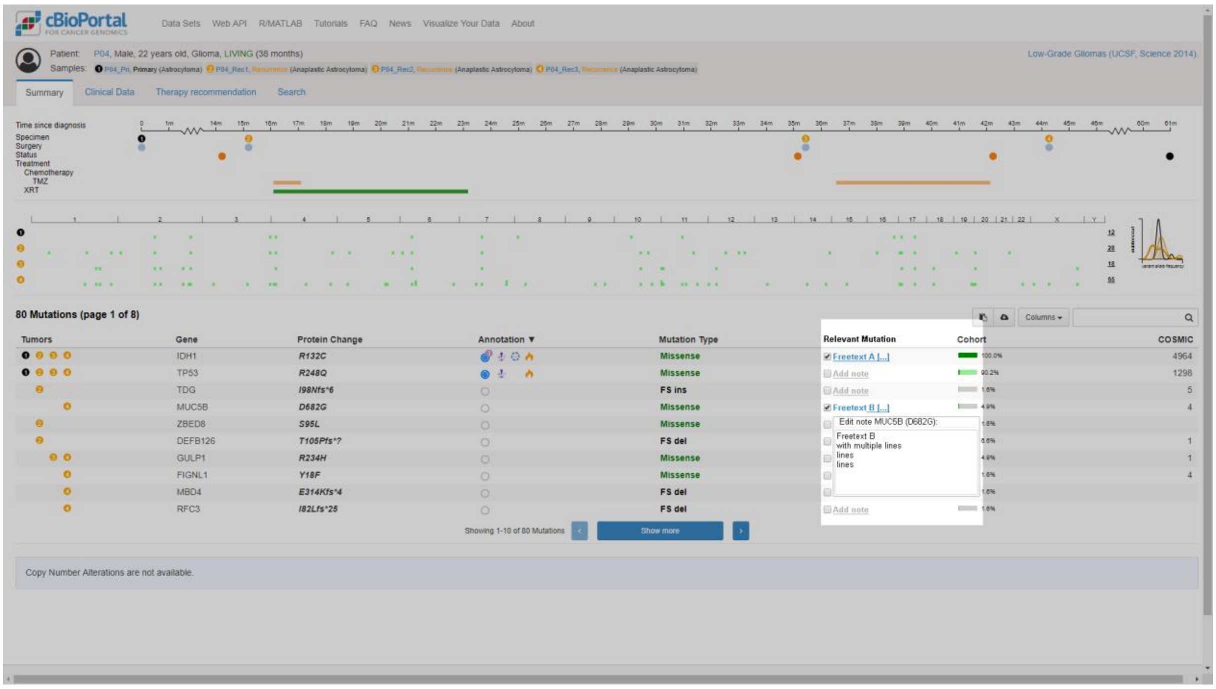

Figure A8. Screenshot mockup demonstrating determination of relevant mutations. Example data adopted from MSKCC's publicly available cBioPortal instance [15].

One step further, there should also be an option to mark a mutation as not relevant explicitly. However, this was only requested by one site.

14 Additional flags for mutations

The interviewees requested a way to manually flag mutations with the three characteristics "predictive", "diagnostic" and "functional" in cBioPortal. It should also be possible to assign these three flags in any combination.

Compared to the pathogenicity classification (see 12 Mutation classification), this feature's importance seems to be lower.

As a further development stage, an automatic assignment based on a pre-consolidated whitelist would be conceivable.

15 Search tool for similar patients

Having a look at a previous, similar patient can help in decision making. This requires a search functionality that lists all the cases with identical mutations previously flagged as relevant; ideally also across all partner sites (five sites requested a MIRACUM-wide search for similar patients while one site demanded a local search only and three sites abstained from voting).

This feature should not only list cases in which the pattern of the relevant mutations corresponds exactly to that of the current patient but should also enable the searcher to parameterize a match rate in the query (for example, at least two out of three mutations). Furthermore, it should also be possible to search for alterations that are not exactly the same but are located on the same gene. An optional limitation to the corresponding tumor entity can further refine the search. Finally, all these parameters should be automatically pre-filled in with the patient's data.

The matching cases found shall be presented in a table. Our mockup (see Figure A9) includes the display of patient identifying data (e.g., an identification number), which links to the corresponding patient view in cBioPortal at the same time. In addition, all relevant mutations, follow-up data (see 16 Follow-up data) and lifetime analysis could be integrated to provide a quick overview of all entries found, which the interviewees considered to be beneficial.

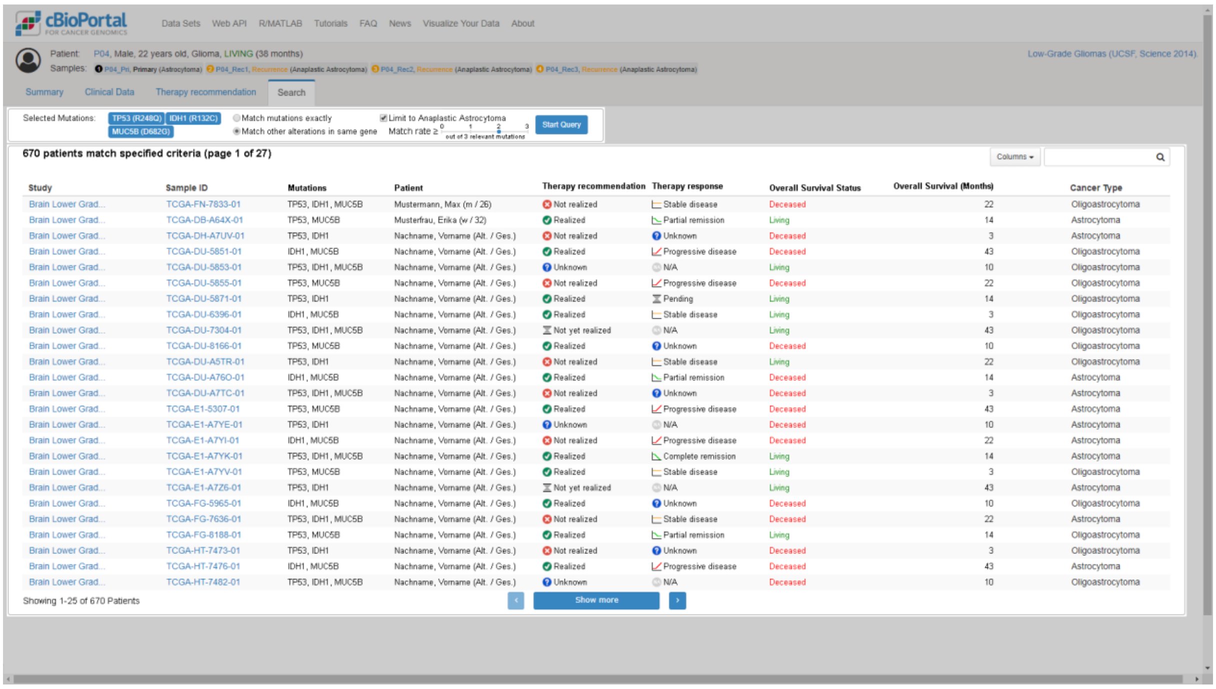

Figure A9. Screenshot mockup demonstrating a function to search for similar patients. Example data adopted from MSKCC's publicly available cBioPortal instance [15].

In addition to linking to the corresponding patient views in cBioPortal for more detailed examination, the interviewees demanded to be able to download the generated PDF reports (see 19 PDF-Report) of all found cases at once.

16 Follow-up data

The participants requested a possibility to record the further course after the MTB has made a therapy recommendation. Therefore, the current status of the therapy according to the recommendation and the effects should be documented (see Table A1, Table A2 and Figure A10). In addition, a text field for short notes should be implemented. For example, this could be used to explain briefly why the therapy was interrupted prematurely.

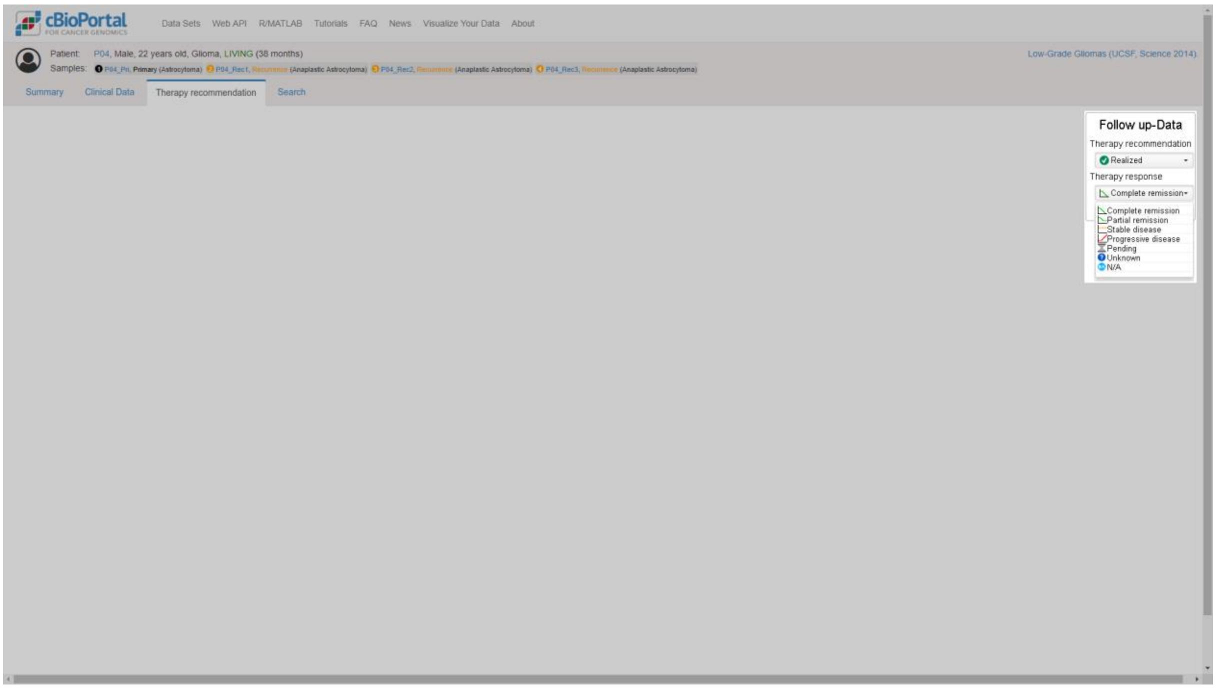

Figure A10. Screenshot mockup demonstrating determination of follow-up data. Example data adopted from MSKCC's publicly available cBioPortal instance [15].

This feedback is of great importance for the work of the MTB. The participants expect improved traceability of already closed cases, and thus a positive effect on future therapy recommendations - not least because new lessons can be learned from each case. Nevertheless, at our partner sites, it is not clear who is responsible for maintaining this data. According to the interviewees, it is not common for those who are responsible for the therapy to give feedback according to these criteria or even report the further development of this case to the Molecular Tumor Board.

Table A1. Status of therapy attempt according to recommendation.

Status	Description
**"Not yet realized"**	The clinicians have decided to realize the therapy according to the recommendation but have not yet started it.
**"Ongoing"**	The therapy according to the recommendation is currently in progress.
**"Completed"**	The therapy according to the recommendation was completed (regardless of the outcome).
**"Premature interruption"**	The therapy according to the recommendation was interrupted after start (e.g., because the patient did not tolerate it).
**"Not realized"**	The therapy has never been started, for whatever reason. For example, if clinicians favored a different therapy.
**"Unknown"**	No feedback could be evaluated.

Table A2. Status/progression of disease.

Status	Description

**“Complete remission”**	The disease was completely cured.
**“Partial remission”**	The disease was partially cured (e.g., decreased tumor volume).
**“Stable disease”**	The state neither improved nor worsened.
**“Progressive disease”**	The disease continues to progress.
**“Pending”**	At the moment, no clear trend is discernible.
**“Unknown”**	No feedback could be evaluated.

17 Search tool for suitable studies

In order to facilitate the search for suitable studies for the current case, such functionality should be implemented in cBioPortal. All sites mentioned a search engine (clinicaltrials.gov [27]) maintained by the U.S. National Library of Medicine as being used as the only platform for this purpose. The search parameters such as the corresponding genes of the mutations marked as relevant or the tumor entity should be included in the query. The interviewees see the inclusion of the age and gender as well as the country in the query as somewhat less relevant, but by no means as obsolete. In order to be able to adapt the request, all parameters should be optional. The above-mentioned affected genes should also be individually selectable (see Figure A11).

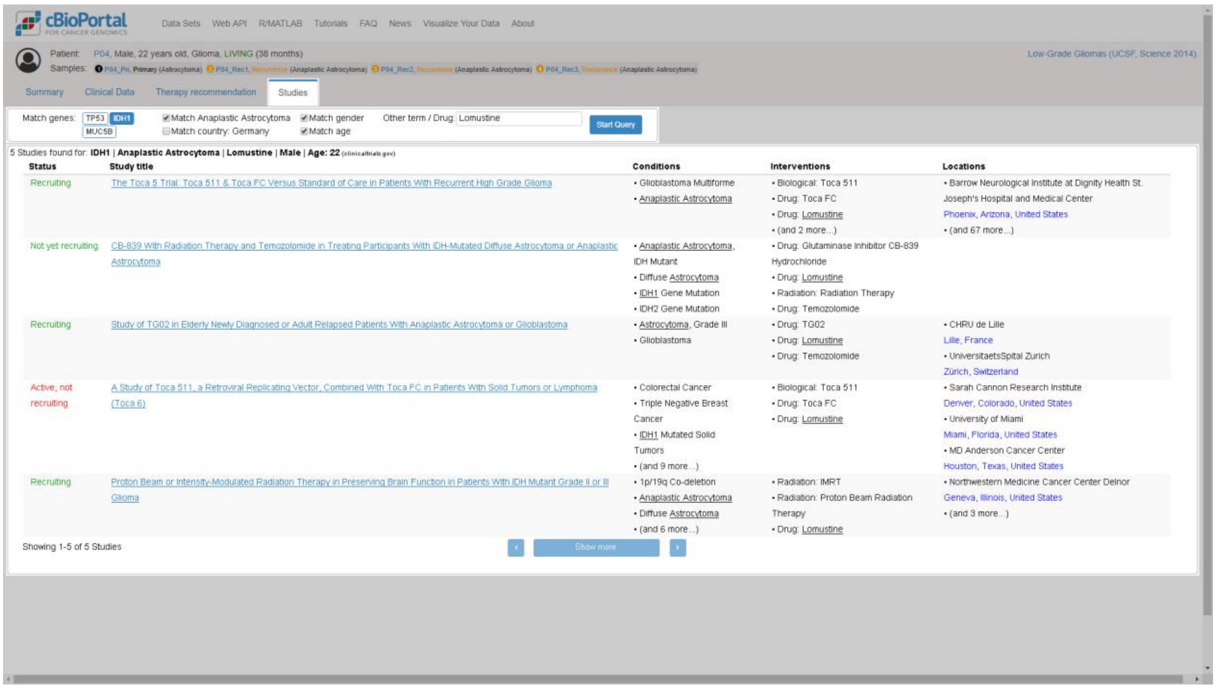

Figure A11. Screenshot mockup demonstrating integration of clinicaltrials.gov. Example data adopted from MSKCC’s publicly available cBioPortal instance [15] and clinicaltrials.gov [27].

In general, being included in a study can pave the way for off-label therapies. According to the interviewees, including a patient in a study is the only option for reimbursement of costs when the clinician cannot prove causality enough to health insurance companies [28]. This is especially important for patients who have already received all standard therapies without a satisfactory outcome, in other words, those discussed in an MTB.

A problem could be the way in which tenders are presented on the above platform. Often only general criteria (such as "in solid tumors") are mentioned in the overview. From this, it may be difficult to deduce whether the current case could fit into the study or not. Natural Language Processing could offer a remedy for this, but this is far beyond the scope of this MIRACUM Use Case 3.

18 Therapy recommendation

The sites requested a way to document the consolidated therapy recommendation of the MTB within cBioPortal. Such a recommendation consists of one or more therapeutic components whose order must be modifiable by the user (for example: "Component A" should be listed before "Component B"). Apart from the actual description of the component (e.g. the name of a drug), no further recommendations regarding the intake will be made as this is at the discretion of the attending physician. However, free text notes will be used for adding more information about a component.

The components must be individually linked to at least one of the mutations, which have been marked as relevant previously (see 13 Therapy relevant mutation). The only exception is when the recommendation is made due to the Tumor Mutational Burden (see 3 Scores). A checkbox should be available to document this, which also deactivates this restriction. Also, the absence of resistances against a component should be explicitly documentable.

Each component must be justifiable by references (including a link and a description with, for example, the title, authors and year of publication). In addition, an evidence level is assigned to each individual component and additionally to all components as an entirety. While the former often results from the respective publications, the overall evidence level is a discretionary decision of the MTB. Finally, the date on which the therapy recommendation was agreed by the MTB should be recorded, too.

In addition, it was demanded that the dataset of the recommendation can be locked and thus only in justified exceptional circumstances modifications are allowed. In this case, the modification must also be clearly indicated.

As an optional feature, the sites requested the documentation of cost reimbursements by the statutory health insurers. This is relevant retrospectively, as it allows a better estimation of the chance of compensation in future, similar cases.

For all of this, eight sites opted for a semi-structured data recording. This means, wherever all possible values of an attribute are known during implementation (e.g., level of evidence), the user input should be structured. However, for example, the notes or the explicit documentation of the absence of a resistance must be recorded in a free text since there may exist almost infinite characteristic attributes (see Figure A12). In contrast, only one site preferred a structured way only (no site abstained from voting).

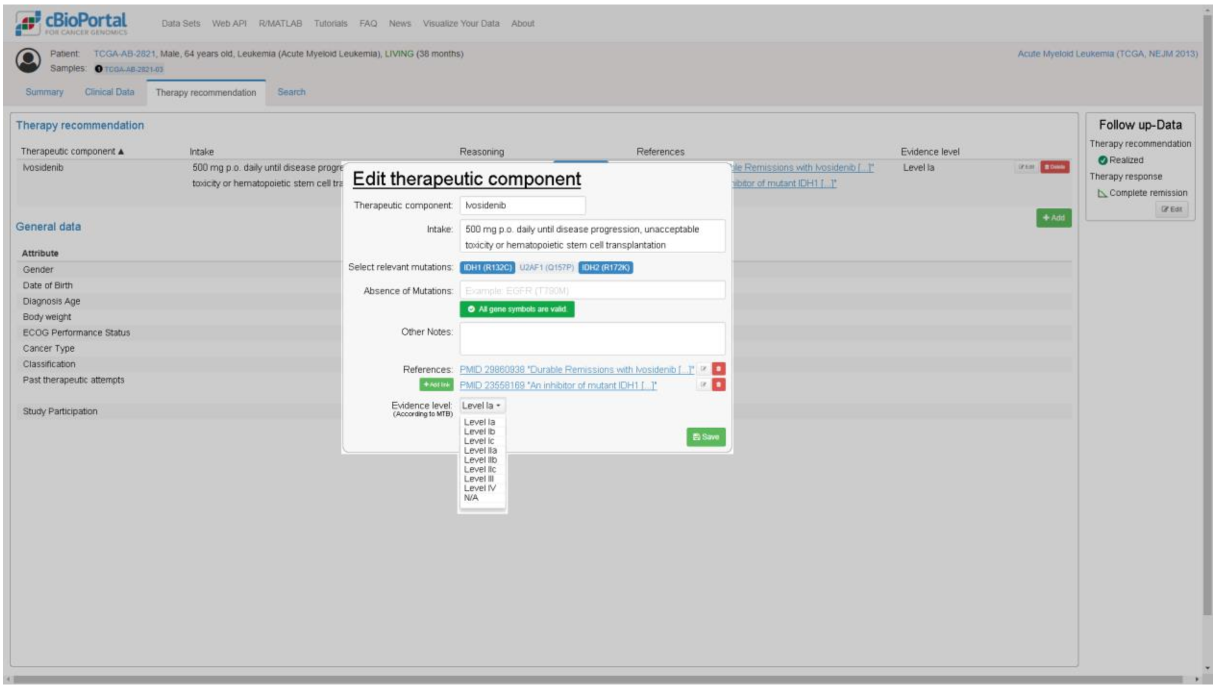

Figure A12. Screenshot mockup demonstrating semi-structured input of therapy recommendation. Example data adopted from MSKCC's publicly available cBioPortal instance [15].

19 PDF-Report

The MIRACUM-Pipe [4] also generates a PDF report with all information available at the time of analysis before the data gets imported into cBioPortal. However, this comprises only information in advance of the analysis by the experts in the MTB. Therefore, it is necessary to be able to export all data stored and manipulated in cBioPortal that led to the final therapy recommendation and to archive those in a revision-proof manner. The interviewees suggested using PDF for this purpose, too.

This report should give an overview of the relevant mutations (affected gene, position, affected amino acids, and proteins) selected previously (four sites requested those to be included automatically instead of selecting them again for the PDF report and the remaining five sites abstained from voting). Also, corresponding data from the annotation databases integrated into cBioPortal should be recorded to be able to comprehend the therapy recommendation even after years when the databases may have been altered. The databases should be selectable for each mutation separately, so that irrelevant or not consulted databases can be excluded (two sites voted for this option while one site preferred to automatically include all entries matching a mutation from all available databases; six sites abstained from voting). Furthermore, all changes made to the data since the import into cBioPortal have to be documented too, including changes to automatically applied classifications (e.g., see 12 Mutation classification) and of course the therapy recommendation (see 18 Therapy recommendation). Four sites requested the therapy recommendation to be included in the report (the remaining five sites abstained from voting).

Since most sites have opted for pseudonymization of all patient data in cBioPortal (see 24 Privacy and pseudonymization), no personal data will be included in this report. Instead, a unique patient ID assigned by a hospital information system should be used for identification.

As an optional feature, the interviewees requested a function to change the order of databases in the report for each relevant mutation individually. So, for example, the corresponding JAX-CKB entry for a

mutation may be more important than the OncoKB entry but for another mutation it maybe vice versa. On the other hand, they do not consider text module generation for creating a continuous text, similar to a doctor's letter, to be relevant.

This report is then passed on to the attending physicians who are responsible for accepting or declining the recommended therapy. The document is also archived at the respective hospitals and thus made available for future investigations.

20 Presentation tool

At the moment, PowerPoint slides with the relevant information are created manually to present the patients at the Molecular Tumor Board meeting [29]. However, there is disagreement on how this process can be supported automatically. Two sites demanded the generation of PowerPoint slides with not further specified default contents. In contrast, one site wanted to use cBioPortal directly with the option to hide unnecessary elements during the presentation. To one site both options are practicable and another site voted against a presentation tool at all. Four sites abstained from voting.

21 Integration into hospital information system

No consolidated statement can be made about the type of the integration of cBioPortal into the system landscape of the respective university hospitals, as too little information is given by the sites (all sites abstained from voting).

During the first round of interviews, it was suggested that the corresponding patient case could be opened in cBioPortal directly from the clinical workstation system via a hyperlink in order to save time. An enhancement to this approach could be the integration of cBioPortal in the corresponding system with the help of IFrames or something similar. This would allow the user to view the data right in cBioPortal and for example directly download a PDF report. Integration into the various system landscapes would also probably be easier to manage.

Optimally, however, the data recorded by the users in cBioPortal gets exported to the corresponding systems. On the one hand, cBioPortal (acting as the client) itself could access a corresponding Application Programming Interface (API) and thus push the data (such as the PDF report) to the clinical workplace system (acting as the server). But since the individual clinics use different systems, the connection to the corresponding APIs would have to be programmed specifically for each site.

On the other hand, a more generic approach would be to pull the changed data from cBioPortal (acting as the server). This could be performed by the clinical workstation system (acting as the client), triggered manually by the user or automatically on a regular basis. For this, an API has to be provided by cBioPortal.

An API should also be used to automate the process of data import into cBioPortal (i.e. clinical data and the annotated variants from our MIRACUM-Pipe [4]). According to the interviewees, this could save a lot of valuable time.

22 Type of access to online services

Regarding the type of integration of the various online services (see, for example, 5 or **Fehler! Verweisquelle konnte nicht gefunden werden.**), the sites demanded to access them online. This implies

that the annotation data is always retrieved on-demand, so these are always up to date. However, this also means that in the event of downtime of such an external service, the information cannot be retrieved. Four sites voted for this option and the remaining five sites abstained from voting.

A compromise solution for this can be a combination of online and offline access. Therefore, the data gets cached on the initial import of patient data into cBioPortal or each time an annotation is viewed on demand. This means whenever all services are working correctly, the latest data is retrieved, but during downtime, the cached data can be displayed as an interim solution.

Of course, if the patient data record is imported into cBioPortal during downtime of an external service, incomplete annotation data could also occur if no information has yet been cached. If, however, the corresponding services go online again and the caching is resumed, they would at least be available in the event of future downtimes.

No matter which variant is implemented, the local conditions of each site must be taken into account with regard to the isolation of the internal clinic network, which may deny communication with external parties. In the most restrictive case (any communication with external servers is denied for the cBioPortal instance), a complete offline data use with manual import of the data records may be considered. However, this should be avoided as far as possible due to the increased maintenance effort.

Table A3. Comparison of online and offline access to external services.

<table>
<tr><th>Access to external services</th><th>online</th><th>offline</th><th>combination</th></tr>
<tr><td>Information during uptime</td><td>latest</td><td rowspan="2">depending on last synchronization</td><td>latest</td></tr>
<tr><td>Information during downtime or other error</td><td>none</td><td>last cached</td></tr>
</table>

23 User and rights management

Several sites demanded the implementation of a comprehensive permission system in cBioPortal. This far-reaching subject area could only be dealt with rudimentarily in the interviews. The main requirements related to a system for authentication, authorization and traceability of changes.

The first two are intended to ensure that the system can grant certain rights to different features to a user whose identity has been verified. For example, it would be conceivable that one user may access the data read-only, while another user may also be allowed to modify data (e.g., to mark a mutation as relevant, see 13 Therapy relevant mutation). It would also be conceivable to restrict access to certain patients for some users.

Traceability is required in order to be able to determine which user made which changes to the data records and when. This is also a prerequisite for locking the therapy recommendation as soon as it has been finalized and for indicating any later changes in exceptional circumstances (see 18 Therapy recommendation). Authentication and Authorization are also mandatory for this purpose.

24 Privacy and pseudonymization

The sites that responded to this question at all did not see any need to store identifying data in cBioPortal. It is therefore sufficient to link the data with unique patient IDs and omit instantly identifying data such as the patient’s name and address (three sites voted for this option while one site requested identifying data to be stored in cBioPortal and five abstained from voting). Furthermore,

the strict General Data Protection Regulation (GDPR) [30] that applies in the European Union and its German supplement, the Federal Data Protection Act (BDSG) [31], must be fulfilled in their currently valid version.

25 References

1. German Federal Ministry of Health. Updated standardized oncological basic data set of the Consortium of German Tumor Centers e.V. (ADT) and the Society of Epidemiological Cancer Registries in Germany e.V. (GEKID) ("Aktualisierter einheitlicher onkologischer Basisdatensatz der Arbeitsgemeinschaft Deutscher Tumorzentren e.V. (ADT) und der Gesellschaft der epidemiologischen Krebsregister in Deutschland e.V. (GEKID)"). Available online: https://www.bundesanzeiger.de/ (accessed on 9 March 2019).
2. OncoTree. Available online: https://github.com/cBioPortal/oncotree (accessed on 19 March 2019).
3. Louis, D.N.; Ohgaki, H.; Wiestler, O.D.; Cavenee, W.K.; Burger, P.C.; Jouvet, A.; Scheithauer, B.W.; Kleihues, P. The 2007 WHO classification of tumours of the central nervous system. *Acta Neuropathol.* **2007**, *114*, 97–109, doi:10.1007/s00401-007-0243-4.
4. Metzger, P.; et al. MIRACUM-Pipe (AG-Boerries/MIRACUM-Pipe repository). Available online: https://github.com/AG-Boerries/MIRACUM-Pipe (accessed on 11 August 2019).
5. González-Pérez, A.; López-Bigas, N. Improving the assessment of the outcome of nonsynonymous SNVs with a consensus deleteriousness score, Condel. *Am. J. Hum. Genet.* **2011**, *88*, 440–449, doi:10.1016/j.ajhg.2011.03.004.
6. Kumar, P.; Henikoff, S.; Ng, P.C. Predicting the effects of coding non-synonymous variants on protein function using the SIFT algorithm. *Nat. Protoc.* **2009**, *4*, 1073–1081, doi:10.1038/nprot.2009.86.
7. Adzhubei, I.A.; Schmidt, S.; Peshkin, L.; Ramensky, V.E.; Gerasimova, A.; Bork, P.; Kondrashov, A.S.; Sunyaev, S.R. A method and server for predicting damaging missense mutations. *Nat. Methods* **2010**, *7*, 248–249, doi:10.1038/nmeth0410-248.
8. Reva, B.; Antipin, Y.; Sander, C. Predicting the functional impact of protein mutations: application to cancer genomics. *Nucleic Acids Res.* **2011**, *39*, e118:1-e118:14, doi:10.1093/nar/gkr407.
9. Ewing, B.; Green, P. Base-calling of automated sequencer traces using phred. II. Error probabilities. *Genome Res.* **1998**, *8*, 186–194.
10. Goodman, A.M.; Kato, S.; Bazhenova, L.; Patel, S.P.; Frampton, G.M.; Miller, V.; Stephens, P.J.; Daniels, G.A.; Kurzrock, R. Tumor Mutational Burden as an Independent Predictor of Response to Immunotherapy in Diverse Cancers. *Mol. Cancer Ther.* **2017**, *16*, 2598–2608, doi:10.1158/1535-7163.MCT-17-0386.
11. Heeke, S.; Hofman, P. Tumor mutational burden assessment as a predictive biomarker for immunotherapy in lung cancer patients: getting ready for prime-time or not? *Transl. Lung Cancer Res.* **2018**, *7*, 631–638, doi:10.21037/tlcr.2018.08.04.
12. Campesato, L.F.; Barroso-Sousa, R.; Jimenez, L.; Correa, B.R.; Sabbaga, J.; Hoff, P.M.; Reis, L.F.L.; Galante, P.A.F.; Camargo, A.A. Comprehensive cancer-gene panels can be used to estimate

mutational load and predict clinical benefit to PD-1 blockade in clinical practice. *Oncotarget* **2015**, *6*, 34221–34227, doi:10.18632/oncotarget.5950.

13. Yarchoan, M.; Hopkins, A.; Jaffee, E.M. Tumor Mutational Burden and Response Rate to PD-1 Inhibition. *N. Engl. J. Med.* **2017**, *377*, 2500–2501, doi:10.1056/NEJMc1713444.

14. Zhang, M.; Yang, J.; Hua, W.; Li, Z.; Xu, Z.; Qian, Q. Monitoring checkpoint inhibitors: predictive biomarkers in immunotherapy. *Front. Med.* **2019**, *13*, 32–44, doi:10.1007/s11684-018-0678-0.

15. Cerami, E.; Gao, J.; Dogrusoz, U.; Gross, B.E.; Sumer, S.O.; Aksoy, B.A.; Jacobsen, A.; Byrne, C.J.; Heuer, M.L.; Larsson, E.; et al. The cBio cancer genomics portal: an open platform for exploring multidimensional cancer genomics data. *Cancer Discov.* **2012**, *2*, 401–404, doi:10.1158/2159-8290.CD-12-0095.

16. Patterson, S.E.; Liu, R.; Statz, C.M.; Durkin, D.; Lakshminarayana, A.; Mockus, S.M. The clinical trial landscape in oncology and connectivity of somatic mutational profiles to targeted therapies. *Hum. Genomics* **2016**, *10*, 4:1–4:13, doi:10.1186/s40246-016-0061-7.

17. van der Velden, D.L.; van Herpen, C.M.L.; van Laarhoven, H.W.M.; Smit, E.F.; Groen, H.J.M.; Willems, S.M.; Nederlof, P.M.; Langenberg, M.H.G.; Cuppen, E.; Sleijfer, S.; et al. Molecular Tumor Boards: current practice and future needs. *Ann. Oncol.* **2017**, *28*, 3070–3075, doi:10.1093/annonc/mdx528.

18. Landrum, M.J.; Lee, J.M.; Riley, G.R.; Jang, W.; Rubinstein, W.S.; Church, D.M.; Maglott, D.R. ClinVar: public archive of relationships among sequence variation and human phenotype. *Nucleic Acids Res.* **2014**, *42*, D980-D985, doi:10.1093/nar/gkt1113.

19. Levy, M.A.; Lovly, C.M.; Horn, L.; Naser, R.; Pao, W. My Cancer Genome: Web-based clinical decision support for genome-directed lung cancer treatment. *JCO* **2011**, *29, doi:*10.1200/jco.2011.29.15_suppl.7576.

20. Griffith, M.; Spies, N.C.; Krysiak, K.; McMichael, J.F.; Coffman, A.C.; Danos, A.M.; Ainscough, B.J.; Ramirez, C.A.; Rieke, D.T.; Kujan, L.; et al. CIViC is a community knowledgebase for expert crowdsourcing the clinical interpretation of variants in cancer. *Nat. Genet.* **2017**, *49*, 170–174, doi:10.1038/ng.3774.

21. Karczewski, K.J.; Francioli, L.C.; Tiao, G.; Cummings, B.B.; Alföldi, J.; Wang, Q.; Collins, R.L.; Laricchia, K.M.; Ganna, A.; Birnbaum, D.P.; et al. Variation across 141,456 human exomes and genomes reveals the spectrum of loss-of-function intolerance across human protein-coding genes **2019, doi:**10.1101/531210.

22. Lek, M.; Karczewski, K.J.; Minikel, E.V.; Samocha, K.E.; Banks, E.; Fennell, T.; O'Donnell-Luria, A.H.; Ware, J.S.; Hill, A.J.; Cummings, B.B.; et al. Analysis of protein-coding genetic variation in 60,706 humans. *Nature* **2016**, *536*, 285–291, doi:10.1038/nature19057.

23. Sherry, S.T.; Ward, M.-H.; Kholodov, M.; Baker, J.; Phan, L.; Smigielski, E.M.; Sirotkin, K. dbSNP: the NCBI database of genetic variation. *Nucleic Acids Res.* **2001**, *29*, 308–311, doi:10.1093/nar/29.1.308.

24. Chakravarty, D.; Gao, J.; Phillips, S.M.; Kundra, R.; Zhang, H.; Wang, J.; Rudolph, J.E.; Yaeger, R.; Soumerai, T.; Nissan, M.H.; et al. OncoKB: A Precision Oncology Knowledge Base. *JCO Precision Oncology* **2017**, *2017, doi:*10.1200/PO.17.00011.

25. Zeukeng, M.-J.; Seoane-Vazquez, E.; Bonnabry, P. A comparison of new drugs approved by the FDA, the EMA, and Swissmedic: an assessment of the international harmonization of drugs. *Eur. J. Clin. Pharmacol.* **2018**, *74*, 811–818, doi:10.1007/s00228-018-2431-7.

26. Richards, S.; Aziz, N.; Bale, S.; Bick, D.; Das, S.; Gastier-Foster, J.; Grody, W.W.; Hegde, M.; Lyon, E.; Spector, E.; et al. Standards and guidelines for the interpretation of sequence variants: a joint consensus recommendation of the American College of Medical Genetics and Genomics and the Association for Molecular Pathology. *Genet. Med.* **2015**, *17*, 405–424, doi:10.1038/gim.2015.30.

27. U.S. National Library of Medicine. ClinicalTrials.gov. Available online: https://clinicaltrials.gov/ (accessed on 10 August 2019).

28. Perera-Bel, J.; Hutter, B.; Heining, C.; Bleckmann, A.; Fröhlich, M.; Fröhling, S.; Glimm, H.; Brors, B.; Beißbarth, T. From somatic variants towards precision oncology: Evidence-driven reporting of treatment options in molecular tumor boards. *Genome Med.* **2018**, *10*, 18:1-18:15, doi:10.1186/s13073-018-0529-2.

29. Hinderer, M.; Boerries, M.; Haller, F.; Wagner, S.; Sollfrank, S.; Acker, T.; Prokosch, H.-U.; Christoph, J. Supporting Molecular Tumor Boards in Molecular-Guided Decision-Making - The Current Status of Five German University Hospitals. *Stud. Health Technol. Inform.* **2017**, *236*, 48–54, doi:10.3233/978-1-61499-759-7-48.

30. *REGULATION (EU) 2016/679 OF THE EUROPEAN PARLIAMENT AND OF THE COUNCIL - of 27 April 2016 - on the protection of natural persons with regard to the processing of personal data and on the free movement of such data, and repealing Directive 95/46/EC (General Data Protection Regulation)*. *GDPR*, 2016.

31. German Federal Ministry of Justice and Consumer Protection. German Federal Data Protection Act ("Bundesdatenschutzgesetz / BDSG"). Available online: https://www.gesetze-im-internet.de/englisch_bdsg/englisch_bdsg.pdf (accessed on 4 April 2019).
